# Identification of key interactions of benzimidazole resistance-associated amino acid mutations in *Ascaris* β-tubulins by molecular docking simulations

**DOI:** 10.1038/s41598-022-16765-4

**Published:** 2022-08-12

**Authors:** Ben P. Jones, Arnoud H. M. van Vliet, E. James LaCourse, Martha Betson

**Affiliations:** 1 Department of Comparative Biomedical Sciences, School of Veterinary Medicine, Faculty of Health and Medical Sciences, University of Surrey, Guildford, GU2 7AL UK; 2 Department of Tropical Disease Biology, Liverpool School of Tropical Medicine, Liverpool, L3 5QA UK

**Keywords:** Computational biology and bioinformatics, Molecular biology

## Abstract

*Ascaris* species are soil-transmitted helminths that infect humans and livestock mainly in low and middle-income countries. Benzimidazole (BZ) class drugs have predominated for many years in the treatment of *Ascaris* infections, but persistent use of BZs has already led to widespread resistance in other nematodes, and treatment failure is emerging for *Ascaris*. Benzimidazoles act by binding to β-tubulin proteins and destabilising microtubules. Three mutations in the β-tubulin protein family are associated with BZ resistance. Seven shared β-tubulin isotypes were identified in *Ascaris lumbricoides* and *A. suum* genomes. Benzimidazoles were predicted to bind to all β-tubulin isotypes using in silico docking, demonstrating that the selectivity of BZs to interact with one or two β-tubulin isotypes is likely the result of isotype expression levels affecting the frequency of interaction. *Ascaris* β-tubulin isotype A clusters with helminth β-tubulins previously shown to interact with BZ. Molecular dynamics simulations using β-tubulin isotype A highlighted the key role of amino acid E198 in BZ-β-tubulin interactions. Simulations indicated that mutations at amino acids E198A and F200Y alter binding of BZ, whereas there was no obvious effect of the F167Y mutation. In conclusion, the key interactions vital for BZ binding with β-tubulins have been identified and show how mutations can lead to resistance in nematodes.

## Introduction

The large intestinal roundworm *Ascaris lumbricoides* infects humans and causes ascariasis. *Ascaris lumbricoides* is a parasitic nematode that resides in the small intestine of its host and can persist there for up to 2 years^1^. Ascariasis is often asymptomatic, but in regions of high *A. lumbricoides* prevalence there can be significant effects on host wellbeing, with chronic ascariasis leading to reduced cognitive ability and stunted growth due to malnutrition^2^. The migrating larvae may also cause pulmonary ascariasis which results in asthma-like symptoms, whilst high worm burdens can lead to more serious pathologies such as organ blockages, which can result in death^3,4^. As of 2019 there was an estimated 446,000 people infected with *A. lumbricoides* worldwide with an estimated loss of 754,000 disability adjusted life years (DALYs)^5^. Most of these infections occur in rural and poor urban areas of low- and middle-income countries, where hygiene and sanitation infrastructure can be of a lower standard than in higher income areas, and therefore people are more exposed to infection. *Ascaris suum* is a closely related roundworm of pigs, although it can also be zoonotic^6,7^. *Ascaris suum* has a wider geographical distribution than *A. lumbricoides* and is one of the most prevalent intestinal parasites of pigs worldwide^8,9^. *Ascaris suum* infection can lead to production losses from reduced growth rates, altered muscle composition and the condemnation of livers due to fibrotic lesions known as milk spots^10,11^.

There are only a small number of drugs available to treat ascariasis, which include the benzimidazoles (BZ), macrocyclic lactones and levamisole^12^. Overreliance on these drugs has led to the potential for drug resistance. Mass drug administration (MDA) of BZ anthelmintics, such as albendazole and mebendazole, in endemic regions is the strategy for control and elimination of a number of helminth diseases in humans, including ascariasis. The most recent 2021 World Health Organization roadmap for neglected tropical diseases has targeted the elimination of ascariasis as a public health problem in 96 countries by reaching 75% coverage of MDA in targeted populations^13^. Whilst repeated treatment in endemic communities may be able to reduce parasite burdens, it does not prevent reinfection, and it is well-established that pressure applied by MDA can lead to the evolution of drug resistance^14^. Benzimidazole resistance has been detected in many intestinal parasites of both veterinary and human importance, and the first signs of reduced susceptibility in *Ascaris* have been reported^15–24^. To date, BZ resistance has been linked to mutations in β-tubulin proteins, more specifically at amino acids 167, 198 or 200, based on the *Haemonchus contortus* β-tubulin reference sequence (accession number: AAA29170.1). Nematodes usually encode multiple β-tubulin isotypes but not all are expressed equally, with some being life-stage or cell-type specific^25^. One of the highly expressed isotypes, β-tubulin isotype 1, is commonly linked to resistance in parasitic nematodes^16,18–21,26–29^. Little is known about the contribution of other β-tubulin isotypes to drug interactions and resistance. Based on evidence from *Caenorhabditis elegans*, it is likely that most of these isotypes are redundant or have specialised roles within specific cells or at certain developmental stages^30^. So far, no work has been done to characterise the roles of the β-tubulins in ascarids or other common STHs and therefore the role they play in drug mechanisms and the development of BZ resistance is still unknown.

One of the biggest hindrances to answering these questions is the ability to culture the full lifecycle of these parasites in vitro, as well as the ethical considerations and costs associated with studying parasites in animal models. In silico approaches could help to solve these problems by predicting the differences seen between proteins and how they interact with drugs. In silico docking is a technique that uses computational software to try and mimic biological systems and monitor molecular interactions. A common use is to model protein–ligand docking to theoretically assess the ability of a ligand to bind within the active sites of a protein and to develop novel drugs^31^. In silico docking has been performed using the β-tubulins of several helminths including *H. contortus*, *Trichinella spiralis* and filarial nematodes^32–35^. These studies have highlighted the changes in protein conformation that occur when resistance mutations are present and how that affects drug interactions. To date these methods have not been applied to *Ascaris*, nor has any study looked into the differences that may be seen between the individual β-tubulin isotypes within a genus or species.

The aims of this study were to investigate the interactions between commonly used BZ drugs and *Ascaris* β-tubulins, and identify what changes occur when mutations are present. The first objective was to predict whether BZ binding in *Ascaris* was similar to that of other helminths. The second objective was to compare the binding of these drugs in each of the β-tubulin isotypes present in *Ascaris*. The final objective was to perform further experiments in proteins that contain the common resistance-associated mutations, to gain an insight into changes that lead to resistance.

## Results

### Identification of *Ascaris* β-tubulin isotypes

Twenty-one β-tubulin sequences were retrieved from NCBI, which were reduced to six after removal of partial and duplicated sequences. BLAST searches against the three *Ascaris* genomes in Wormbase-Parasite identified a total of 122 matches, 51 of which were β-tubulins based on nomenclature and identity to reference sequence, with the remainder α-tubulins. Of the 51 β-tubulins, after duplicates had been removed, there were 8 sequences remaining from the *A. lumbricoides* genome (GCA_000951055.1), seven from one *A. suum* genome (GCA_000298755.1) and six from a second *A. suum* genome (GCA_000187025.3). To extend the search for more distantly related tubulins, the Exonerate program predicted the presence of several additional tubulin sequences in the three *Ascaris* genomes. A search of the Conserved Domain Database revealed that most were α-tubulins, but a new β-tubulin sequence was identified for *A. suum* (E′) and a more complete sequence for *A. lumbricoides* B′ sequence was identified and added to the sequences used for phylogenetic analysis (Supplementary Table S1). The isotype G identified from *A. suum* (GCA_000187025.3) was found to be split into two consecutive genes in the genome annotation, although manual alignment of these two genes with isotype G from *A. lumbricoides* confirmed that these two genes represented two halves of the full gene with an incorrect stop codon predicted at the end of an exon (at position 169–171 of the cDNA). Therefore, these two consecutive genes were concatenated and used as the *A. suum* isotype G gene for all further work (Supplementary Fig. S1). The protein to gene alignment undertaken with the Exonerate program on the two newly available genomes (*A. lumbricoides* GCA_015227635.1 and *A. suum* GCA_013433145.1) found sequences for all isotypes, with the exception of isotype G in *A. lumbricoides*. These sequences were added to the existing data and a phylogeny was created which included β-tubulins from other Ascaridomorpha species. A full list of sequences used can found in Supplementary Tables 1 and 2.

**Figure 1:**
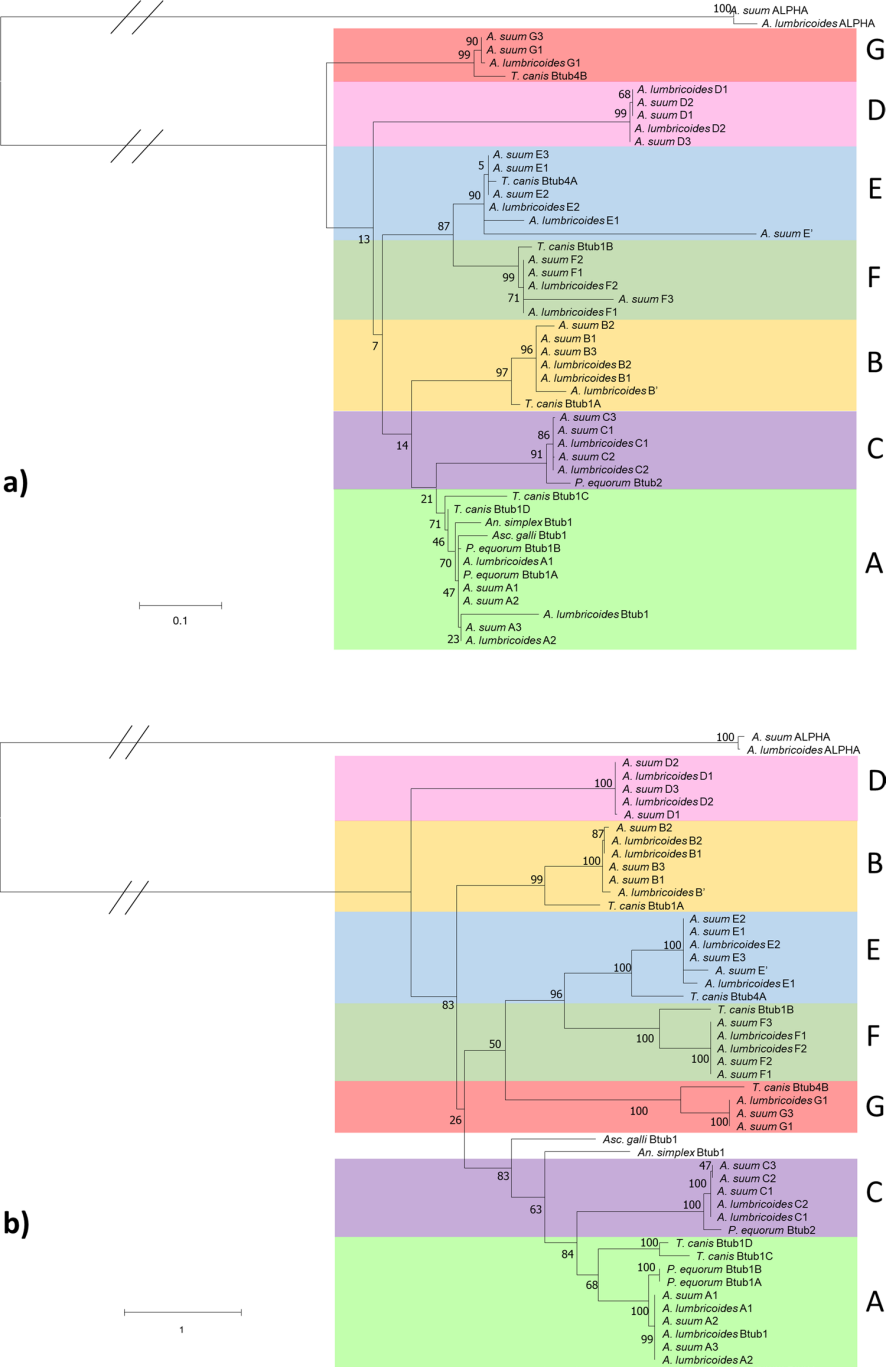
Phylogenetic reconstruction of Ascaridomorpha β-tubulins. Phylogenies show the relationship between each isotype from the *Ascaris* genomes as well as previously published Ascaridomorpha species β-tubulins. (**a**) Shows the phylogeny reconstructed using the peptide sequences under the assumptions of the JTT + G model. (**b**) Shows the nucleotide phylogeny reconstructed under the K2 + G + I model. Both phylogenies underwent 1000 bootstraps. Bootstrap values are shown at each node. Sequences collected for members of the Ascaridomorpha have retained the nomenclature given in the database (e.g., *Toxocara canis* β-tub4B). The species included were *Anisakis simplex*, *Ascaridia galli*, *Parascaris equorum* and *Toxocara canis*. For the *Ascaris* sequences identified from the genomes each sample is named by species, isotype and then genome number (e.g., *Ascaris suum* C3 is the isotype C sequence from *A. suum* genome 3).

The phylogenetic tree showed a clear separation into definitive isotypes that appear to have diverged early in the evolution of the Ascaridomorpha infraorder (Fig. 1). When phylogenetic trees for the amino acid and nucleotide sequences were compared, the structuring of the isotype clades were not consistent, although similar relationships were observed between sequences within clades. *Ascaris suum* isotype F3 had one truncated exon and so did not fit into the group as well as other sequences. *Ascaris suum* isotype E′ was also seen to be divergent from the rest of the isotype E group, and as this sequence was only found in one genome it was not designated its own isotype. The effects of these sequence variations were seen more clearly in the phylogenetic tree based on amino acid sequences.

Only the seven isotypes that had homologues in both *Ascaris* species were used in further analysis. Isotype A clustered with sequences from other species, such as *Parascaris*, that have been previously linked with BZ interaction through gene expression studies, and is the isotype which is currently used in diagnostic tests for BZ resistance in *Ascaris*^15,16,22,36,37^. For this reason, isotype A was used as the focus of molecular docking simulations. Interestingly the isotype previously designated as isotype-1 in *A. suum* did not fall within isotype A, but instead was found to be isotype C, suggesting the past labelling of this sequence as isotype-1 was incorrect^20^.

### In silico docking shows similar binding for all β-tubulin isotypes

**Figure 2:**
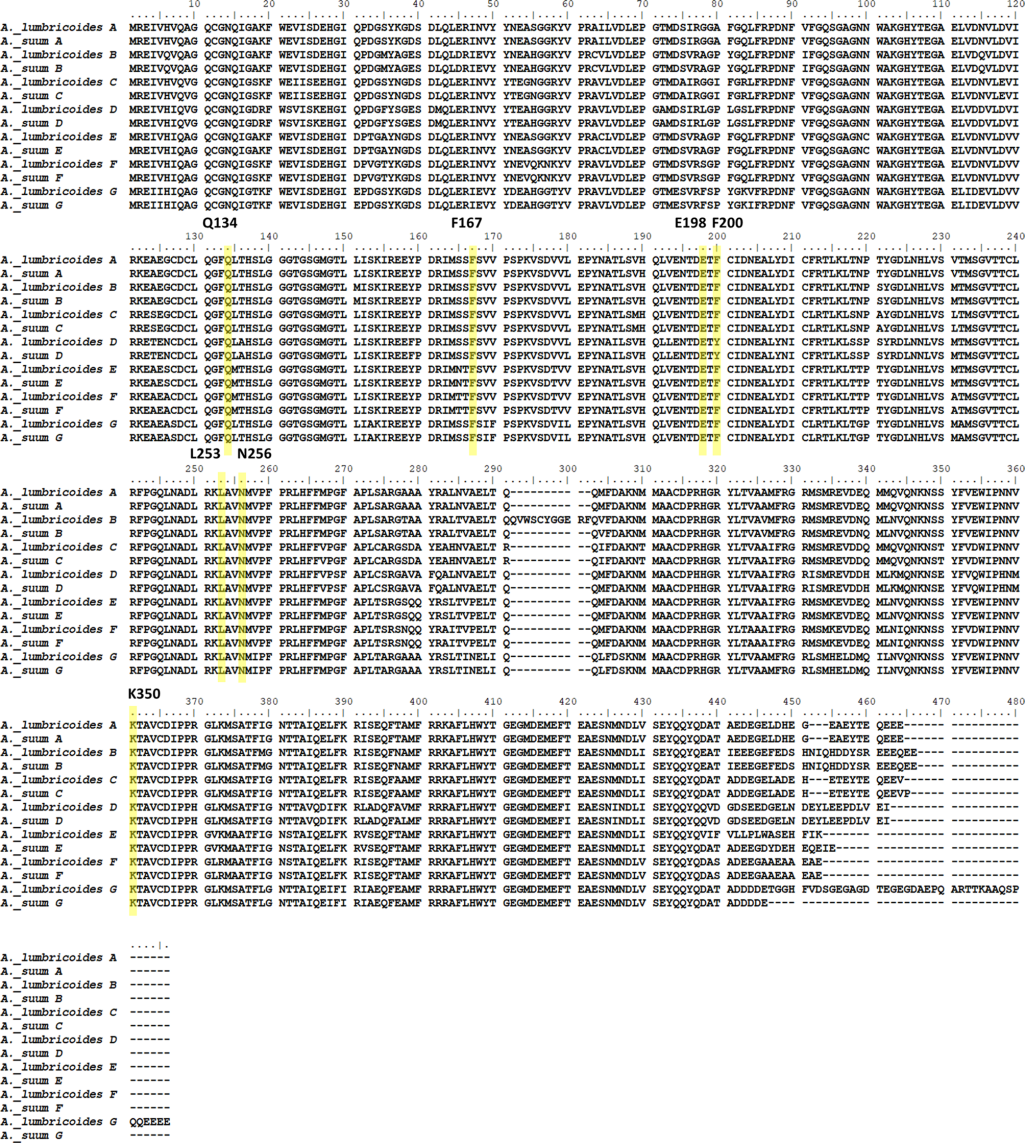
Representative amino acid alignment of *Ascaris* β-tubulins. Alignments for each *Ascaris* β-tubulin isotype used in docking simulations. The common resistance associated amino acids (F167, E198 and F200Y) and the amino acids that were found to interact with BZs (Q134, L253, N256 and K350) and may be of some importance are highlighted in yellow.

**Figure 3:**
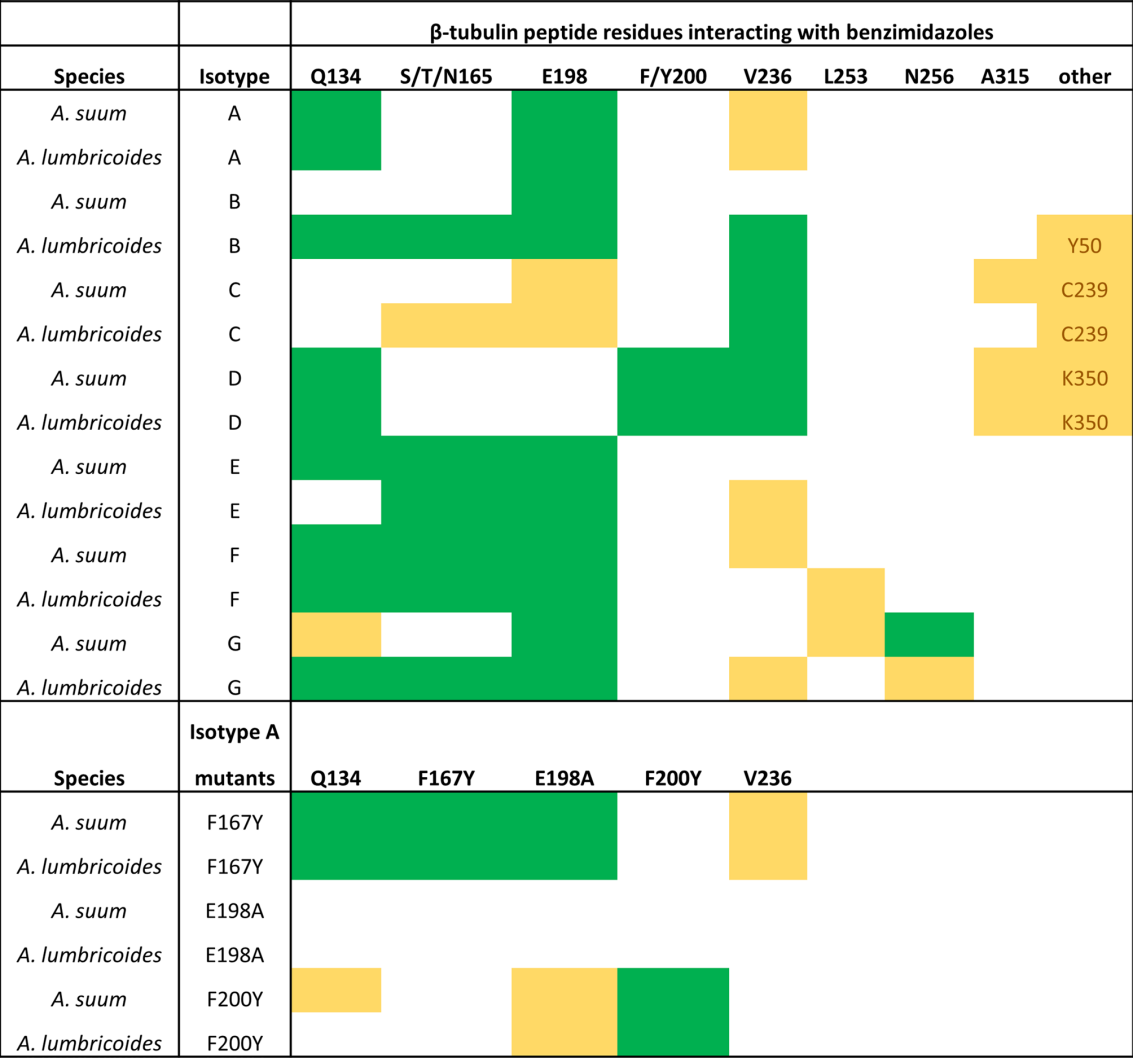
Ligand docking amino acid binding frequencies. The amino acids in *Ascaris lumbricoides* and *Ascaris suum* that form bonds with benzimidazole drugs in the ligand docking simulations are shown. Green represents an amino acid that interacts with more than one drug and yellow represents an interaction seen only once.

The quality checks of minimised homology models show acceptable results; with Z-scores within the expected range in ProSA based on comparison with all structures in PDB database. Verify3D scores showed over 80% of the structures passed the threshold for the 3D structure matching the 1D predictions and no bond angles were found to be in disallowed regions with PROCHECK (Supplementary Figs. 2–21, Supplementary Table 3). In silico ligand docking simulations were performed on the seven β-tubulin isotypes shared by both *Ascaris* species. An alignment of each isotype highlighting some active site amino acids can be seen in Fig. 2. Five BZ drugs were docked into the common BZ binding pocket of each isotype and simulations showed a consistent trend between species, drug and isotype. However, the 3D structures and the 2D maps were not always in complete agreement when labelling hydrogen bonds (H-bonds). Hydrogen bond formation between BZs and amino acids Q134, E198 and V236 were the most common interactions and were consistently seen in all isotypes. Amino acid A315 and the amino acids at position 165 were also seen numerous times in docking poses. In the majority of cases amino acid 165 was a serine (S), although in isotypes E and F, amino acid 165 was asparagine (N) and threonine (T) respectively. These changes did not cause a change to the overall amino acid properties as all three amino acids are polar (neutral) hydrophobic amino acids, and similar interaction were observed between the drugs and all three amino acids. Other amino acids interacted with the BZs in some isotypes, and although these were not consistently seen, they could be of some importance and would require further investigation (Fig. 3).

Isotype D had tyrosine (Y) at position 200 and this formed bonds with glutamate (E) at position 198. Isotype D was the only isotype to naturally contain tyrosine at position 200 which has been linked to resistance when seen in other β-tubulins^21,27^. Recent work in *Parascaris* has shown that having tyrosine as the wildtype amino acid in this β-tubulin isotype is not restricted to *Ascaris* only^38^.

It was only in isotype D and the mutated F200Y models that binding was seen between the drugs and amino acid 200. In the mutated F167Y protein models, the mutation of phenylalanine (F) to tyrosine resulted in extra bonds being formed with the drugs in most cases. In the mutated E198A models no bonds were formed with E198A in any drug model. The models with the F200Y mutation showed a bond between the mutated F200Y amino acid and E198. The full details of the binding of each drug to each individual isotype are provided in Supplementary Figs. S22–S41.

### Molecular dynamics simulations highlight BZ resistance mechanisms

Molecular dynamics simulations calculate the pressure and heat energies that are likely found within a physiological system and apply these to the protein–drug structure to mimic natural systems over a period of time to find the optimum binding poses. These simulations show how protein–drug interactions fluctuate over a period of time and give an indication of how these molecules may react in a physiological system. As the molecular docking simulations showed no difference between species or isotype, molecular dynamics simulations were performed only on *A. suum* isotype A. Simulations showed no major changes from the initial ligand docking.

**Figure 4:**
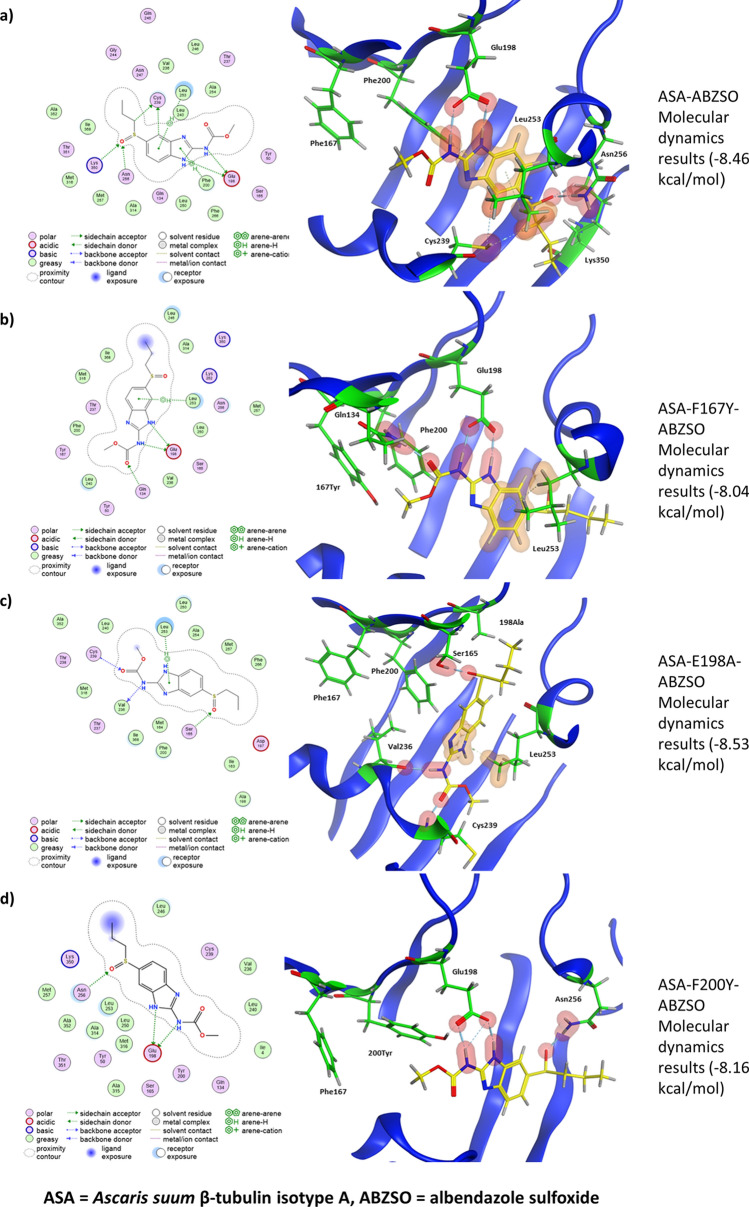
2D and 3D representations of the 100 ns molecular dynamics simulations of *Ascaris suum* isotype A wildtype and mutant models with ABZSO. The figure shows the protein–ligand interaction made between each model. In 2D models (left) bonds formed with residues are depicted with dashed lines with the specific type of bond being described in the legend. All residues shown in 2D models without bonds are predicted to interact via van der Waals forces. In the 3D models (right) the protein structure is shown in ribbon format (blue) with only binding residues or the resistance associated residue highlighted shown in full (green). Hydrogen bonds (H-bonds) between protein and ligand are highlighted in red and arene bonds are highlighted in amber. The drug ABZSO is shown in yellow. Binding affinity shown to the right of each model. (**a**) In the wildtype model H-bonds can be seen between the drug and E198, C239, N256 and K350. Arene bonds are also formed with F200 and L253. (**b**) In the F167Y mutated model H-bonds can be seen between the drug, Q134 and E198. An arene bonds is also seen with L253. (**c**) In the E198A mutated model H-bonds can be seen between the drug, S165, V236 and C239. An arene bonds is also seen with L253. It is noticeable that this is the only model that does not have bonds with residue 198. (**d**) In the F200Y mutated model H-bonds can be seen between the drug, E198 and N256. In the F200Y model so that the bond between E198 and 200Y is made visible.

**Figure 5:**
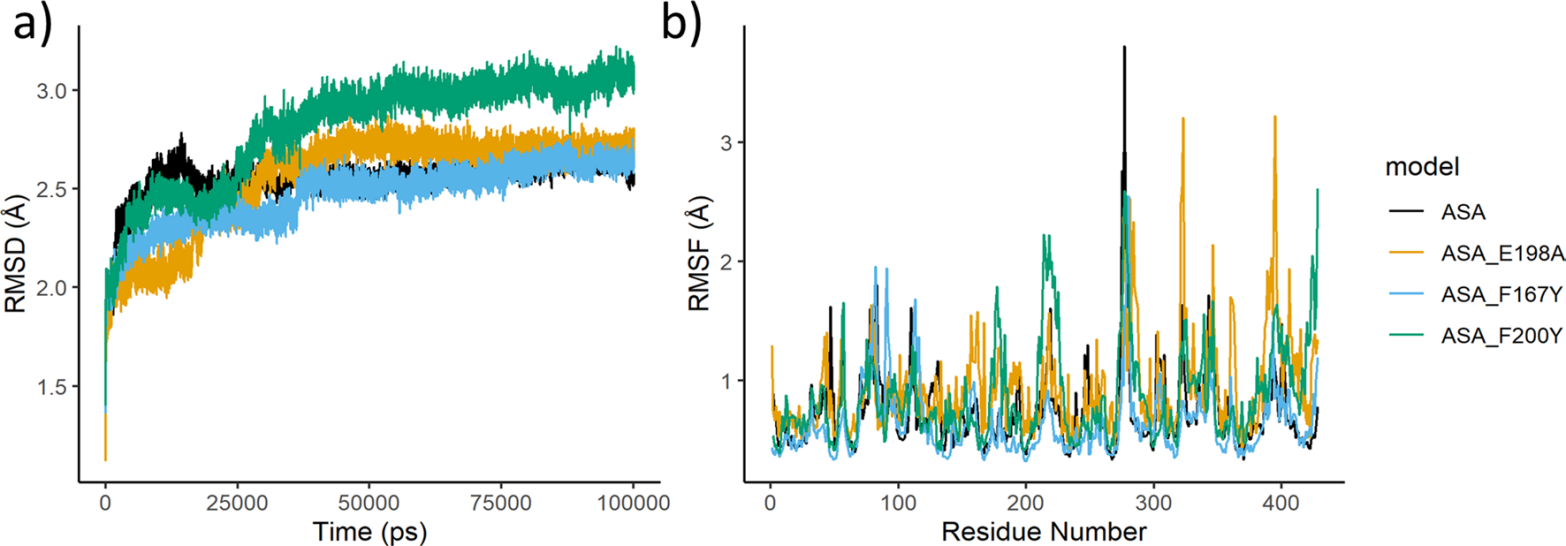
Root-mean-square deviation (RMSD) and root-mean-square fluctuation (RMSF) analysis of molecular dynamics simulations. The graphs show (**a**) the RMSD and (**b**) the RMSF of each of the structures throughout the molecular dynamics simulations. The RMSD shows that after the initial burn-in the structures are equilibrated and stable, as indicated by the consistency of the RMSD values. The RMSF values show how much each amino acid fluctuates through the simulations and highlights those that fluctuate the most. The wild-type model is shown in black, the F167Y model is shown in orange, the E198A model is shown in blue and the F200Y model is in green.

**Figure 6:**
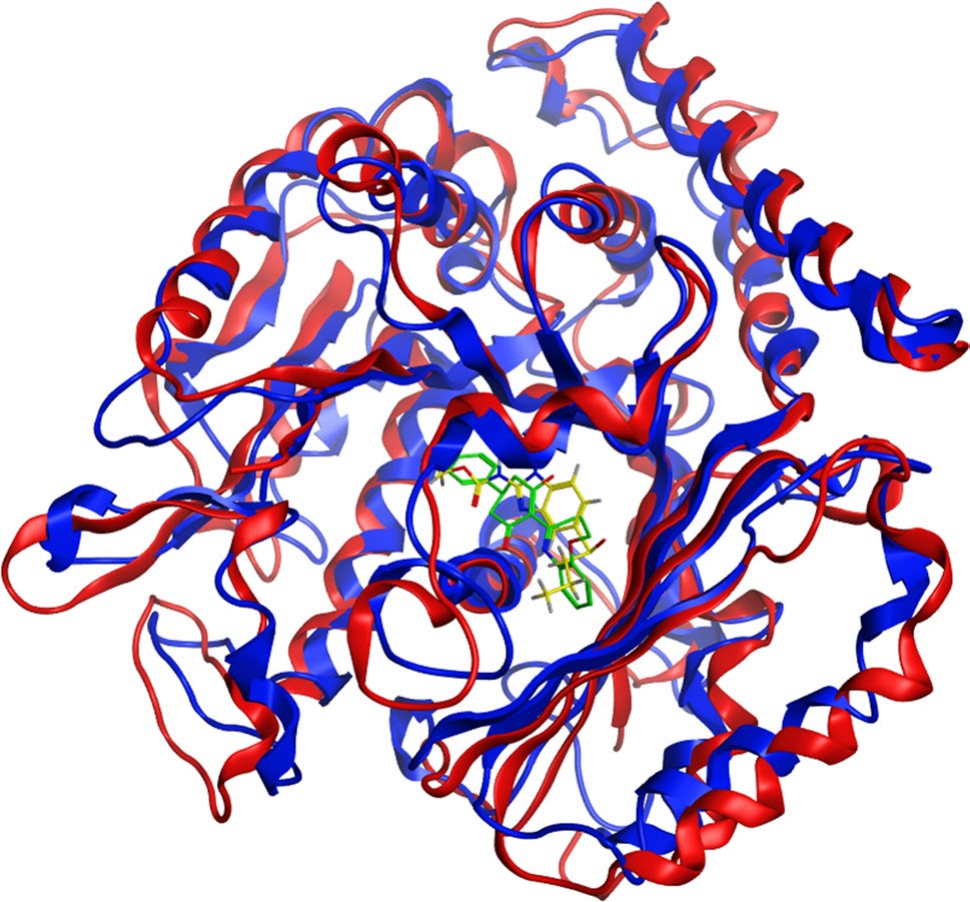
Superposition comparison of reference crystal structure and *Ascaris suum* homology model. The reference crystal structure used to create homology models (6FKJ) is shown in red and the representative *A. suum* isotype A structure which showed the greatest binding affinity from the molecular dynamics simulations is shown in blue. The bound ligands are also shown with TUB075 from the reference structure in green and ABZSO from our *A. suum* simulations in yellow. The overall RMSD between the structures is 2.52 Å.

The molecular dynamics simulations showed that the key E198 interaction seen in the docking studies remained and had high binding affinity (Fig. 4a, Table1). In the mutated F167Y model the strong bond with E198 is still seen but there are overall less interactions compared to the wildtype. A H-bond is formed with Q134 which was previously hypothesised to interact with the mutated F167Y amino acid, however no interaction between these amino acids was seen (Fig. 4b, Table 1). The E198A model was the only model that showed no interaction with residue 198 and resulted in a loss of the strong bond that this residue normally provides. There were some interactions seen between the protein and ligand in this model however none were of the magnitude that is seen with E198 (Fig. 4c, Table 1). Finally, for the F200Y simulation the strong interaction with E198 was restored and only one other interaction was seen with N256. There was also a self-binding interaction seen between E198 and the mutated F200Y residue in these models which could affect drug binding (Fig. 4d, Table 1). Root-mean-square deviation analysis (RMSD) over each step of the molecular dynamics simulations showed that all simulations had reached equilibrium (Fig. 5a). Root-mean-square fluctuation (RMSF) of each amino acid residue over the whole simulation time showed that most residues were stable and had only small levels of fluctuation (Fig. 5b). Superposition of the reference crystal structure (6FKJ) and the *A. suum* isotype A structure showed how similar the two structures were, with an RMSD of 2.52 Å (Fig. 6).

**Table 1:** Interactions between albendazole sulfoxide and specific amino acids in *Ascaris suum* β-tubulin proteins. The proteins used in these analyses were *Ascaris suum* β-tubulin ASA, the three mutated ASA proteins. The table shows the type of bonds formed (H—hydrogen bond, A—arene bond), the amino acid the bond is formed with, and the drug used. The energy of the bonds between the amino acid and drug are given in kcal/mol, the distance between the bonded atoms is given in Angstroms (Å) and the number of bonds formed between the amino acid and the drug is shown (frequency). The overall drug binding affinity is given in kcal/mol. ASA = *Ascaris suum* β-tubulin isotype A, ABZSO = albendazole sulfoxide.

Protein–drug model	Type	Amino acid	Drug	Energy (kcal/mol)	Distance (Å)	Frequency	Affinity (kcal/mol)
ASA-ABZSO	H	Glu198	ABZ0	− 17.8	2.76	2	− 8.46
	A	Phe200	ABZ0	− 0.8	3.72	1	
	H	Cys239	ABZ0	− 1.4	3.65	2	
	A	Leu253	ABZ0	− 0.5	4.32	1	
	H	Asn256	ABZ0	− 5.6	2.86	1	
	H	Lys350	ABZ0	− 1.9	3.21	1	
ASA-F167Y-ABZSO	H	Gln134	ABZ0	− 2.9	2.81	1	− 8.04
	H	Glu198	ABZ0	− 18	2.76	2	
	A	Leu253	ABZ0	− 0.7	4.38	1	
ASA-E198A-ABZSO	H	Ser165	ABZ0	− 3.6	2.68	1	− 8.53
	H	Val236	ABZ0	− 6.9	2.8	1	
	H	Cys239	ABZ0	− 4.4	2.86	1	
	A	Leu253	ABZ0	− 1.1	3.75	1	
ASA-F200Y-ABZSO	H	Glu198	ABZ0	− 17.5	3.02	3	− 8.16
	H	Asn256	ABZ0	− 5.6	2.81	1

Further comparisons of the effect of mutations on *A. suum* isotype A were seen in the PremPLI results which showed that the F167Y, E198A and F200Y mutations lead to a reduced binding affinity with ΔΔG of 0.47, 0.8, 0.57 kcal/mol respectively.

## Discussion

The widespread resistance to BZs in ruminant nematodes such as *H. contortus* has illustrated the effects that resistance can have on both animal health and economic returns^39^. We have not yet seen widespread resistance to BZs in *Ascaris* in either humans or pigs, although, with increasing drug pressure to reach the 2030 World Health Organisation targets, limited studies on drug efficacy in either humans or pigs, and limited alternative treatments, a better understanding of the mechanisms leading towards BZ resistance in *Ascaris* is urgently required. This work identified seven β-tubulin isotypes shared by both *Ascaris* species considered here, and compared, in silico*,* BZ interactions between them. Previous modelling attempts have found that the BZ binding site is inaccessible when the protein is in its closed conformation^32^. A structure that had a colchicine binding site inhibitor within the binding region was used as a template for our models to ensure that the binding site remained accessable^40^.

We observed that all β-tubulin isotypes are predicted to interact with BZs in a similar manner, except for one isotype that contains a resistance-associated amino acid at position 200 in its wildtype protein. In silico ligand docking and molecular dynamics simulations highlighted E198 as a key amino acid in BZ-binding, with E198A mutations leading to weaker protein–drug interaction. We also found that the common resistance associated F200Y mutation acts indirectly by binding to E198 and reducing drug stability within the binding pocket.

By utilising multiple databases, we were able to identify seven β-tubulin isotypes from both *A. suum* and *A. lumbricoides*. Phylogenetic analysis showed that *Ascaris* β-tubulin isotypes were shared with other Ascaridomorpha species, and it is isotype A that is used as a marker of BZ resistance and is usually referred to as isotype-1^15,16,22,36,37^. The identification of isotype A as the main group involved in BZ interaction allowed in silico work to focus on this isotype. Concurrent work in *Ascaris* by Roose et al.^41^ also found these same β-tubulin isotypes in both species and identified isotype A as the isotype used in previous surveillance studies. Isotype A was shown to be the most highly expressed β-tubulin isotype across various life-stages and tissue types and therefore one of the main isotypes likely to be involved in BZ interaction^41^. Whilst the expression levels of the β-tubulin isotypes in *Ascaris* have been determined using quantitative reverse transcription PCR on different life-stages of *Ascaris* and also by using data from transcriptome analysis, the contribution of these isotypes to drug mechanisms of action have not yet been defined^41^. Our work has shown that the drug interaction with these isotypes does not differ on the whole, with the exception of isotype D. Therefore, it likely that the contribution of each isotype to drug-binding is relative to the expression level during the different stages of the *Ascaris* life-cycle.

The most common binding amino acid predicted from molecular dynamics simulations was E198. Several other amino acids interacted with the BZs, although these were not consistent. Most of these interactions had weak binding affinity, although K350 was shown to form stronger bonds and could be of potential importance. It has been assumed that E198 is the key binding amino acid for BZs, and indeed the key role E198 has in BZ binding and the self-binding interaction between amino acid E198 and F200Y in the mutated models was observed^33,34^.

The molecular dynamics simulations yielded very similar interactions to previous β-tubulin crystal structures containing other colchicine binding site inhibitors that act in the same way as the BZs studied here^40,42^. All structures show bonds with E198 and both crystal structures show a close proximity to C239 which has been predicted to form a water mediated bond^40^. Our results suggest that E198 is the key amino acid in β-tubulin for BZ binding in *Ascaris,* as interactions were seen in every model except for the mutated E198A structure. Bonds with E198 also showed the strongest binding affinity; at least three times as strong as any other amino acid interaction in most cases. In models that contained the E198A mutation, the change led to a loss of interaction at this important site. In F200Y simulations, the self-binding between E198 and F200Y was observed, which could lead to the blocking or destabilising of interactions between BZs and E198, resulting in resistance to BZs. In F167Y models there was no clear change, and this lack of any clear negative effect may explain why the F167Y mutation has been found in field isolates of *A. lumbricoides* without any effect on drug susceptibility^23^. It has been hypothesised that the F167Y mutation leads to self-binding with amino acids that close off the binding pocket and prevent the drugs from entering^34^. In our work no such self-binding could be seen between the tyrosine at position 167 and any other amino acids. The PremPLI analysis similarly showed all three mutations to have a negative effect on binding affinity (by virtue of the positive ΔΔG values), and again it was the E198A mutation that showed the greatest change.

Previous studies have found that water mediated bonds between the ligands and β-tubulin may play an important role in drug binding^43,44^. These water mediated bonds have been associated with amino acid C239, with evidence for the exact interaction varying from being a water mediated bond between C239 and the ligand, a water mediated bond between the ligand and amino acids C239 and G235 and a direct H-bond between the drug and C239^34,40,42–44^. Whether direct or water mediated, interactions between ligands and C239 could play an important role in drug binding in addition to the core E198 interactions in β-tubulins. In this study we did not include surrounding water molecules so it is possible that the interactions with C239 may have been missed, however in our 100 ns simulations a H-bond was seen directly between ABZSO and C239 in the wildtype and mutated E198A models.

It has been noted that the carbonyl side chain of β-tubulin E198 is likely to be found in its protonated (neutral) form and that this may have an important role in the formation of H-bonds and interacting with water molecules^43^. Our simulations were run using the deprotonated (ionic) form of E198. This had no clear effect on the ability of E198 to form H-bonds with the BZ in the results presented here, with strong bonds seen in all but the E198A mutated model. However, as previously mentioned, the simulations run here lack surrounding water molecules which may have an effect on the system and could be a point for further analysis. Benzimidazole resistance is common for *H. contortus* and other clade V nematodes but is yet to become a common problem for *Ascaris*. In all the searches for drug resistance in *Ascaris* to date only the three common resistance associated mutations, F167Y, E198A and F200Y have been investigated, which means the contributions of other mutations that may affect the BZ susceptibility will be missed. There are reports of ascarid helminths displaying reduced susceptibility to BZ, but do not contain these classical mutations, and hence there is a possibility that there may be other mechanisms or mutations involved in BZ resistance^15,38^. In this study several other amino acids were identified as possible candidates, such as C239, N256 and K350 (see Fig. 3 and Table 1 for full list of interacting amino acids), that may play an important role in drug binding and may lead to BZ resistance if mutations occur.

In conclusion, we have identified the full repertoire of β-tubulin genes from *A. lumbricoides* and *A. suum* and have shown that whilst almost all have the potential to interact with BZs, there is one isotype, isotype A, that is likely key to BZ binding. By identifying the importance of isotype A, our findings will allow future studies to refine and focus their approach to studying the effects of BZs in non-clade V nematodes and monitor resistance development. Our results show that E198 is a vital amino acid for BZ binding of β-tubulins in *Ascaris*, as has been seen for other helminths species; and the E198A and F200Y mutations both take effect by disrupting this key anchor point.

## Methods

Five *Ascaris* genomes: two for *A. lumbricoides* (GCA_000951055.1 and GCA_015227635.1) and three for *A. suum* (GCA_000298755.1, GCA_000187025.3 and GCA_013433145.1) were analysed to identify potential β-tubulin isotypes. Based on previous literature it was found that one *A. lumbricoides* β-tubulin gene had been characterised and deposited in the National Center for Biotechnology Information (NCBI) along with 21 β-tubulin sequences from *A. suum*^20,22,45^. These sequences were retrieved from the database and the *A. suum* sequences were aligned with each other to remove the partial sequences that were duplicates of the longer sequences. The β-tubulin gene from *A. lumbricoides* (EU814697.1) retrieved from NCBI was used to carry out BLAST^46^ searches against the three available *Ascaris* genomes in WormBase-Parasite (GCA_000187025.3, GCA_000298755.1 and GCA_000951055.1)^47,48^. To ensure that no β-tubulin genes had been missed, the paralogues of each gene were checked, and the search term “tubulin beta” was used for each annotated genome. An α-tubulin sequence for each species was also retrieved to be used as the outgroup in further analysis.

Exonerate v2.2.0^49^ protein2genome was used to identify any β-tubulin genes within the *Ascaris* genomes that had not been detected by BLAST or in the genome annotation. Each isotype retrieved from the database search was run against all three genomes with the best 10 results being saved from each test. This number of tests were saved as we found up to eight potential isotypes from the database searches, and this allowed for the potential of at least two further sequences to be identified. Any new sequence found by Exonerate was tested in the Conserved Domain Database^50^ to check that the sequence was a β-tubulin gene and then any new sequences predicted to be β-tubulins were added to the β-tubulin dataset. The two newest genomes (*A. lumbricoides* GCA_015227635.1 and *A. suum* GCA_013433145.1) had not been fully annotated and so Exonerate protein2genome was used to identify β-tubulin genes.

After all the sequences, both nucleotide and peptide, had been collected, they were aligned with tubulin sequences from other Ascaridomorpha using the MUSCLE server (available at: https://www.ebi.ac.uk/Tools/msa/muscle/ [Accessed 09 December 2020])^51^ and a maximum likelihood phylogeny was created with MEGA version X^52^, using the JTT + G model for the amino acid sequences and the K2 + G + I model for the nucleotide sequences. Each phylogeny was bootstrapped 1000 times. Genomes are numbered in the phylogenies as follows: *A. lumbricoides* 1 (GCA_000951055.1); *A. lumbricoides* 2 (GCA_015227635.1); *A. suum* 1 (GCA_000298755.1); *A. suum* 2 (GCA_000187025.3) and *A. suum* 3 (GCA_013433145.1). The peptide sequences of these genes were used to create homology models.

### Homology models

Homology models were created for all β-tubulin isotypes of *A. lumbricoides* and *A. suum* using SWISS-MODEL server (available at: https://swissmodel.expasy.org/ [Accessed 22 February 2021])^53,54^. The β-tubulin crystal structure 6fkj was used as the reference structure. This structure was chosen as it is an experimentally determined crystal structure containing multiple α/β-tubulin dimers with a ligand bound in the colchicine binding site in a similar way as predicted previously for BZs. The ligand bound to this structure is a cyclohexanedione derivative called TUB075 used as a tubulin targeting, antiproliferation cancer drug^40^. Sequences for β-tubulin isotype A were edited to provide sequences with the common BZ resistance associated mutations (F167Y, E198A and F200Y)^17,24^. These were again submitted to SWISS-MODEL to create homology models. This isotype was used as it was this isotype that had been identified as being highly expressed in previous studies of Ascaridomorpha^37^.

### Quality checks

Homology models were submitted to multiple servers for quality checks to confirm the validity of structures created in SWISS-MODEL and help predict any potentially erroneous sites. ProSA-web (Protein Structure analysis) server (available at: https://prosa.services.came.sbg.ac.at/prosa.php [Accessed 15 March 2021])^55,56^ assesses protein model quality. Verify3D^57,58^ compares the 3D structure of the model to the 1D peptide sequence. PROCHECK v3.5^59^ analyses the structural geometry of the protein structures using Ramachandran plots. Both Verify3D and PROCHECK are part of the UCLA SAVES v6.0 server (available at: https://saves.mbi.ucla.edu/ [Accessed 15 March 2021]).

### Energy minimisation

Structures were minimised using the YASARA energy minimization server (available at: http://www.yasara.org/minimizationserver.htm [Accessed 25 February 2021])^60^. This server uses the YASARA forcefield to optimise the positions of atoms and reduce interatomic energies. After all structures were minimised quality checks were performed again.

### In silico ligand docking

3D ligand structure files for commonly used BZ drugs were downloaded from PubChem^61^ in SDF format. The drugs used include three of the most commonly used BZ, albendazole (ABZ), mebendazole (MBZ) and fenbendazole (FBZ), as well as albendazole sulfoxide (ABZSO) and oxfendazole (OXBZ), which are the active metabolites of ABZ and FBZ respectively. These were converted into pdb format using Pymol v2.3.4^62^. Pdb structures of ligands were uploaded to Autodock tools v1.5.6^63,64^. The number of allowable rotatable bonds was set to maximum, and structures were saved in pdbqt format suitable for docking simulations. Protein models were uploaded to Autodock tools to be prepared for docking simulations. Water was deleted from the protein structures; polar hydrogens were added, and structures were saved in pdbqt format. The docking grid was centred on amino acid 200 of the protein as this is the primary amino acid believed to be associated with BZ resistance. Grid spacings were set to 1 Angstrom (Å) and box size was set to 24 Å for x, y and z sizes. This grid box encased all three resistance-associated amino acids within a small pocket of the protein and the co-ordinates of the box were saved for later use.

Autodock vina v1.1.2^65^ was used to perform in silico ligand docking simulations between the β-tubulin isotypes and BZ drugs, using the grid co-ordinates and spacings to identify the target binding region and an exhaustiveness level of 8. Docking results were opened in Pymol to view the 3D structure and interactions. Polar contacts between the drug and proteins were identified and protein–ligand complexes were exported in pdb format. Protein–ligand complexes were opened in Discovery studio v20.1.0.19295^66^ to create 2D ligand interaction maps which show multiple types of interaction between the protein and ligand in a clear and easily read format.

### Molecular dynamics

Molecular dynamics simulation were carried out using Molecular Operating Environment (MOE) 2020.01^67^. The β-tubulin structures were optimised using the Protonate3D method with default settings in MOE. The site finder algorithm was then implemented to identify binding pockets within the protein. The pocket corresponding to the known binding region of BZs was selected and dummy atoms were inserted as markers for the docking. Initial docking simulations were run for ABZSO with the *A. suum* β-tubulin isotype A and its mutated models using the dummy atoms as the site of binding. The initial scoring of docking poses used the London dG method to identify the best 30 ligand poses. This was followed by final scoring of the best 10 poses using GBVI/WSA dG method. The results of the MOE docking were then used for molecular dynamics simulations using the NPA algorithm and the Amber10: EHT forcefield using default configurations. Structures were equilibrated for 100 picoseconds (ps) at 300°K before a production run of 100 ns at 300°K with a time step of 0.002 ps. Once completed, the binding energy of each interacting amino acid and the overall energy in the binding pocket is calculated.

A selection of timesteps were taken every 1 ns for the last 10 ns of the simulation. For each of the selected timesteps the ligand was constrained, and the structure was minimised to give the binding affinity of the ligand. The pose with the strongest binding affinity was then selected as the final result and 2D and 3D representations of the final model were saved. Due to the similarity between species and β-tubulin isotypes based on ligand docking simulations, only *A. suum* isotype A complexes were subject to this analysis. To assess whether simulations had reached equilibrium RMSD analysis of each pose of the molecular dynamics simulations compared to the initial structure were performed in MOE using the alpha carbon atoms. To assess the fluctuations of each amino acid residue throughout the simulations, RMSF calculations were made for the alpha carbon atoms of the structures in MOE. A comparison of the initial reference structure (6FKJ) and the representative *A. suum* isotype A pose from the dynamics simulations with the greatest affinity to ABZSO was made by superimposing the structures and measuring RMSD in MOE. As an independent assessment of the effect of mutations on *A. suum* β-tubulin isotype A the PremPLI server was used to predict the changes in binding affinity that would be caused by the three common resistance-associated mutation (available at: https://lilab.jysw.suda.edu.cn/research/PremPLI/ [Accessed 22 June 2022])^68^.

## Supplementary Information

Supplementary Information.

## Data Availability

The genomic datasets analysed during the current study are available in the Wormbase-Parasite and NCBI repositories: *A. lumbricoides* 1 (GCA_000951055.1) https://parasite.wormbase.org/Ascaris_lumbricoides_prjeb4950/Info/Index/; *A. lumbricoides* 2 (GCA_015227635.1) https://www.ncbi.nlm.nih.gov/genome/11969?genome_assembly_id=1482971; *A. suum* 1 (GCA_000298755.1) https://parasite.wormbase.org/Ascaris_suum_prjna80881/Info/Index/; *A. suum* 2 (GCA_000187025.3) https://parasite.wormbase.org/Ascaris_suum_prjna62057/Info/Index/; *A. suum* 3 (GCA_013433145.1) https://www.ncbi.nlm.nih.gov/genome/350?genome_assembly_id=925559.
