# Supplementary material for: Identification of key interactions of benzimidazole resistance-associated amino acid mutations in Ascaris β-tubulins by molecular docking simulations
Source: Sci Rep. 2022 Aug 12;12:13725. doi: 10.1038/s41598-022-16765-4 (PMC9374697; doi:10.1038/s41598-022-16765-4)
Supplement: Supplementary file 1 — Supplementary Information. [file 41598_2022_16765_MOESM1_ESM.pdf]

Identification of key interactions of benzimidazole resistance-associated amino acid mutations in *Ascaris* β-tubulins by molecular docking simulations

Ben P. Jones[1], Arnoud H.M. van Vliet[2], E. James LaCourse[3], Martha Betson[1*]

1,2. Department of Veterinary Epidemiology and Public Health[1] and Department of Pathology and Infectious Diseases[2], School of Veterinary Medicine, Faculty of Health and Medical Sciences, University of Surrey, Guildford GU2 7AL, United Kingdom

3. Department of Tropical Disease Biology, Liverpool School of Tropical Medicine, Liverpool L3 5QA, United Kingdom

*Corresponding author: Martha Betson email: m.betson@surrey.ac.uk

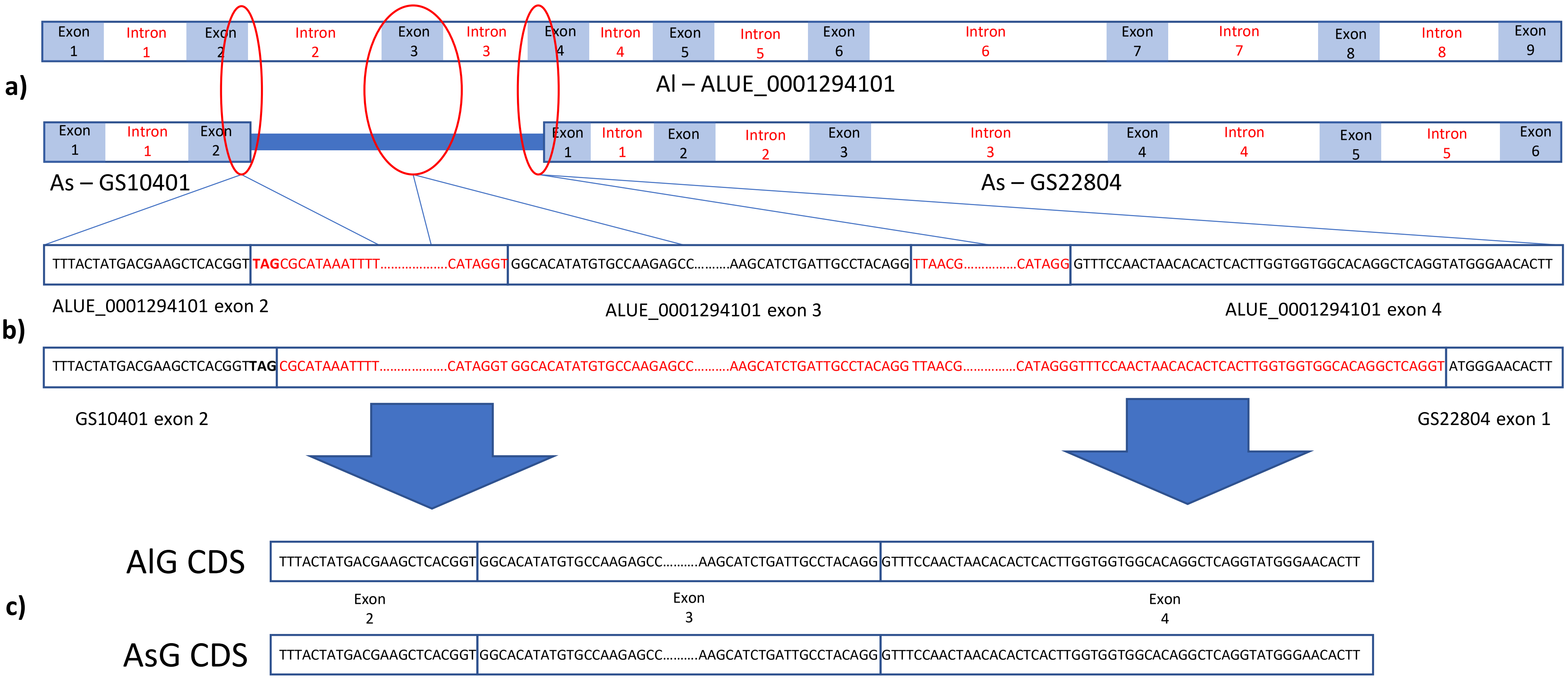

Supplementary Figure S1. Determination of *Ascaris* β-tubulin isotype G sequence. The discrepancies in the available genomes resulted in a truncated sequence for *Ascaris suum* β-tubulin isotype G. To create a complete sequence for *A. suum* β-tubulin isotype G the coding sequence from *Ascaris lumbricoides* (ALUE_0001294101) was aligned with the genomic scaffold of *A. suum* (GCA_000298755.1 scaffold 150). The alignment revealed that genomic sequences were the same in *A. suum* and *A. lumbricoides* however annotation of the genes differed. (a) shows that ALUE_0001294101 aligned to a region containing two genes in *A. suum* (GS_10401 and GS_22804) with a region of non coding sequence separating them. (b) shows and enhanced view of the two *Ascaris* genes in the region where disparities are seen with black text showing exons and red text showing exons. In *A. suum* the GS_10401 contains two exons which match the first two exons of the *A. lumbricoides* gene, however in *A. suum* a TAG stop codon is included whereas in *A. lumbricoides* this sequence is in the intron (TAG codon shown in bold). There is a region between the two genes in *A. suum* which covers the region were exon 3 in *A. lumbricoides* is. GS_22804 starts within exon 4 of *A. lumbricoides*, however it doesn't include the start of exon 4 as a small region is missed before the first ATG start codon is reached. After this point the *A. lumbricoides* and *A. suum* sequences match with each other. Full stops indicate that a length of sequence has been removed for illustration. (c) the exon and intron positions from *A. lumbricoides* were mapped to the *A. suum* scaffold and used to predict the coding sequence (CDS) for the full *A. suum* β-tubulin isotype G. AlG and AsG show the region of the CDS that has been modified from the original in *A. lumbricoides* β-tubulin isotype G and *A. suum* β-tubulin isotype G respectively.

Supplementary Table S1. β-tubulins identified in *Ascaris* genomes. Table shows genome identifiers of the β-tubulin isotypes found in the *Ascaris* genomes. Information on the scaffold/chromosome is given along with the start and stop positions of each gene and which strand the gene is found on (F- forward strand, R- reverse strand). Three genomes were accessed through Wormbase-Parasite (*Ascaris lumbricoides* (GCA_000951055.1); *Ascaris suum* (GCA_000298755.1 and GCA_000187025.3)). Two addition genomes were downloaded from NCBI (*Ascaris lumbricoides* (GCA_015227635.1) and *Ascaris suum* (GCA_013433145.1)).

Genome	Isotype	Gene identifier	Scaffold	Start	End	Strand
Ascaris lumbricoides						
GCA_000951055.1	A	ALUE_0000927201	470	57079	61212	F
	B	ALUE_0000986501	555	88104	95361	R
	B'	ALUE_0001827701	3406	8964	15029	F
	C	ALUE_0000494801	77	278876	284803	F
	D	ALUE_0001031701	629	12771	18776	F
	E	ALUE_0000949301	501	88208	98499	R
	F	ALUE_0000949201	501	63518	77036	R
	G	ALUE_0001294101	1173	40528	50229	F
Ascaris suum						
GCA_000298755.1	A	GS 23993	581	536551	556407	F
	B	GS 01240	1232	159135	165526	R
	C	GS 11145	353	1098450	1104172	F
	D	GS 13691	576	10531	16524	F
	E	GS 05353	4	82719	93772	R
	F	GS 11773	4	63146	74606	R
	G	GS 22804/GS 10401	150	392918	401515	F
Ascaris suum						
GCA_000187025.3	A	AgB02-g235	AgB02	3972969	3980469	F
	B	AgR022-g106	AgR022	1339279	1348771	F
	C	AgE31-g003	AgE31	35111	46174	F
	D	AgR043-g091	AgR043	1472802	1487383	R
	E	AgB01-g252	AgB01	3803075	3814443	R
	E'	N/A	AgE03	198483	201247	F
	F	AgB01-g251	AgB01	3782705	3795903	R
Ascaris lumbricoides						
GCA_015227635.1	A	SMSY01000002.1	ALgV5B02	3964570	3969295	F
	B	SMSY01000143.1	ALgV5R022	1332956	13392117	F
	C	SMSY01000067.1	ALgV5E31	35157	40897	F
	D	SMSY01000164.1	ALgV5R043	1467814	1464808	R
	E	SMSY01000001.1	ALgV5B01	3774732	3764809	R
	E'	SMSY01000039.1	ALgV5E03	186236	190335	F
	F	SMSY01000001.1	ALgV5B01	3755621	3744200	R
Ascaris suum						
GCA_013433145.1	A	JACCHR010000021.1	Chr X2	7870786	7875616	F
	B	JACCHR010000005.1	Chr 5	3747868	3754006	F
	C	JACCHR010000005.1	Chr 5	5413755	5424572	F
	D	JACCHR010000014.1	Chr 14	5946712	5952717	F
	E	JACCHR010000008.1	Chr 8	6785808	6774748	R
	F	JACCHR010000008.1	Chr 8	6766690	6755420	R
	G	JACCHR010000006.1	Chr 6	10203269	10211715	F

Supplementary Table S2. Helminth β-tubulins. Table shows accession numbers for the addition helminth sequences used in this study. The *Ascaris lumbricoides* β-tubulin sequence was used as the reference gene to search databases for β-tubulin isotypes. The Ascaridomorpha sequences (*Parascaris equorum, Ascaridia galli, Anisakis simplex* and *Toxocara canis*) were included in phylogenies to gain insights into the relationship of the β-tubulins isotypes in this family of helminths. *Toxocara canis* sequences were only stored under peptide sequences in NCBI database, however the nucleotide sequences were provided in the peptide accession information. The *Haemonchus contortus* sequence was used for *in silico* analysis as a comparison with previous research.

β-tubulin sequence	Accession	
	DNA	Peptide
Ascaris lumbricoides β-tub 1	EU814697.1	ACJ01792.1
Parascaris equorum β-tub 1A	KC713797.1	AGM37949.1
Parascaris equorum β-tub 1B	JN034256.1	AEJ35172.1
Parascaris equorum β-tub 2	KC713798.1	AGM37950.1
Ascaridia galli β-tub 1	KC713796.1	AGM37948.1
Anisakis simplex β-tub 1	KP326559.1	AKI85333.1
Toxocara canis β-tub 1A	>>	KHN85540.1
Toxocara canis β-tub 1B	>>	KHN79356.1
Toxocara canis β-tub 4A	>>	KHN79367.1
Toxocara canis β-tub 4B	>>	KHN85618.1
Toxocara canis β-tub 1C	>>	KHN83955.1
Toxocara canis β-tub 1D	>>	KHN85343.1
Haemonchus contortus β-tub 1	M76493.1	AAA29170.1

Supplementary Table S3. Quality checks on minimised β-tubulins homology models. Table shows results of multiple checks of structural quality of β-tubulin homology models. QMEAN and GMQE scores are predicted by SWISS-MODEL as part of the homology model creation. ProSA Z-scores were calculated by ProSA-web and are determind by calculating the energy deviation of the model compared to random conformations. The Z-score can be compared to all other proteins in the PDB database (as seen in Supplementary Figs 2-21a). All models Z-scores fell within the scores of seen in the database. Verify3D scores show the percentage of the 3D structure of the protein that fits what would be predicted based on the 1D amino acid sequence (individual amino acid and averaged scores for this analysis can be seen in Supplementary Figs 2-21d). All models passed the 80% threshold of having the 3D and 1D structure match. PROCHECK assesses the structural geometry of the protein structures using Ramachandran plots (as seen in Supplementary Figs 2-25c). PROCHECK calculates the phi-psi bond angles of each residue and compares them with a database of known observations, to indicate whether each amino acid's bonds fall within allowed regions. No models showed any bonds in disallowed regions. ALA – *Ascaris lumbricoides* β-tubulin isotype A; ASA – *Ascaris suum* β-tubulin isotype A.

Isotype	ALA	ALA_F167Y	ALA_E198A	ALA_F200Y	ALB	ALC	ALD	ALE	ALF	ALG	ASA	ASA_F167Y	ASA_E198A	ASA_F200Y	ASB	ASC	ASD	ASE	ASF	ASG
**QMEAN score**	-1.15	-1.18	-1.17	-1.1	-1.77	-0.78	-0.96	-0.72	-1.25	-1.31	-1.15	-1.18	-1.17	-1.1	-0.79	-0.78	-0.96	-0.9	-1.25	-1.2
**GMQE score**	0.96	0.96	0.96	0.96	0.74	0.93	0.88	0.98	0.98	0.82	0.96	0.96	0.96	0.96	0.75	0.93	0.88	0.98	0.98	0.84
**ProSA (Z-score)**	-9.44	-8.99	-9.36	-9.43	-9.2	-9.8	-9.39	-9.81	-9.35	-9.24	-9.44	-8.99	-9.36	-9.43	-9.61	-9.8	-9.39	-10	-9.35	-9.56
**Verify3D (%)**	99.3	97.2	99.53	99.77	99.1	98.1	90.6	96.95	98.4	96.9	99.3	97.2	99.53	99.77	97.7	98.1	90.6	100	98.4	99.3
**PROCHECK ramachandran plot**																				
**core (%)**	93.3	92.7	93.3	94.9	92.1	94.1	93.3	93	93	94	93.3	92.7	93.3	94.9	94.4	94.1	93.3	93.8	93	94.9
**allowed (%)**	6.7	7.3	6.7	5.1	7.3	5.9	6.7	7	6.7	6	6.7	7.3	6.7	5.1	5.6	5.9	6.7	6.2	6.7	5.1
**generously allowed (%)**	0	0	0	0	0.5	0	0	0	0.3	0	0	0	0	0	0	0	0	0	0.3	0
**disallowed**	0	0	0	0	0	0	0	0	0	0	0	0	0	0	0	0	0	0	0	0

ASA

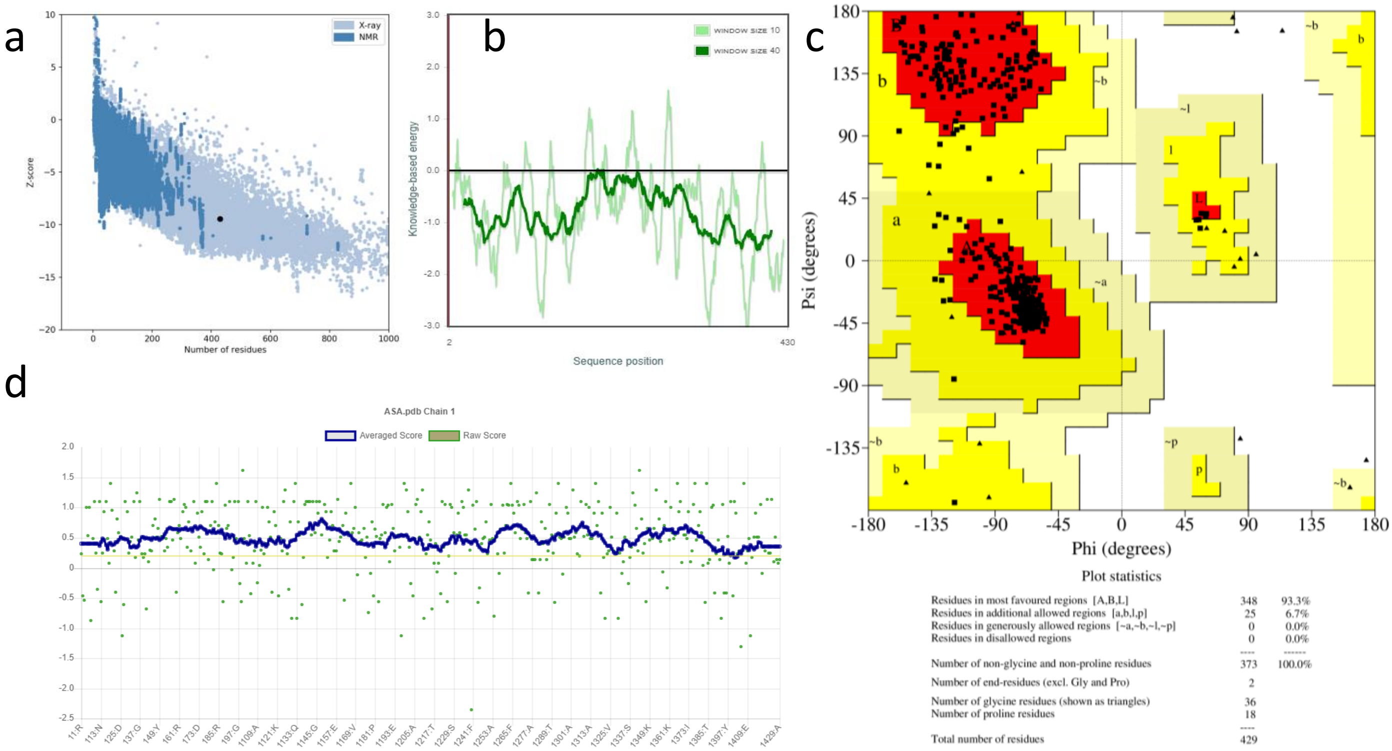

Supplementary Figure S2 – Quality checks on *Ascaris suum* isotype A minimized protein structure. a) ProSA-web global Z-score compared to all other proteins in the PDB database. The black dot shows the Z-score of the modelled protein. Proteins with Z-scores within the range of those from the PDB database show a protein with realistic structure. b) ProSA-web local model energies averaged over sliding windows of 10 and 40 residues. Positive values indicate potential erroneous regions. c) PROCHECK Ramachandran plot shows the torsion angles of each residue predicting whether they are in possible combinations. d) Verify3D results which compares the 3D structure to the amino acid sequence. Results more than 0.2 indicate that the structure of the protein matches what would be predicted from the amino acid sequence. Proteins with more than 80% of residues above the 0.2 threshold pass the criteria. All tests suggest that the minimized homology model is suitable for analysis.

ASA_F167Y

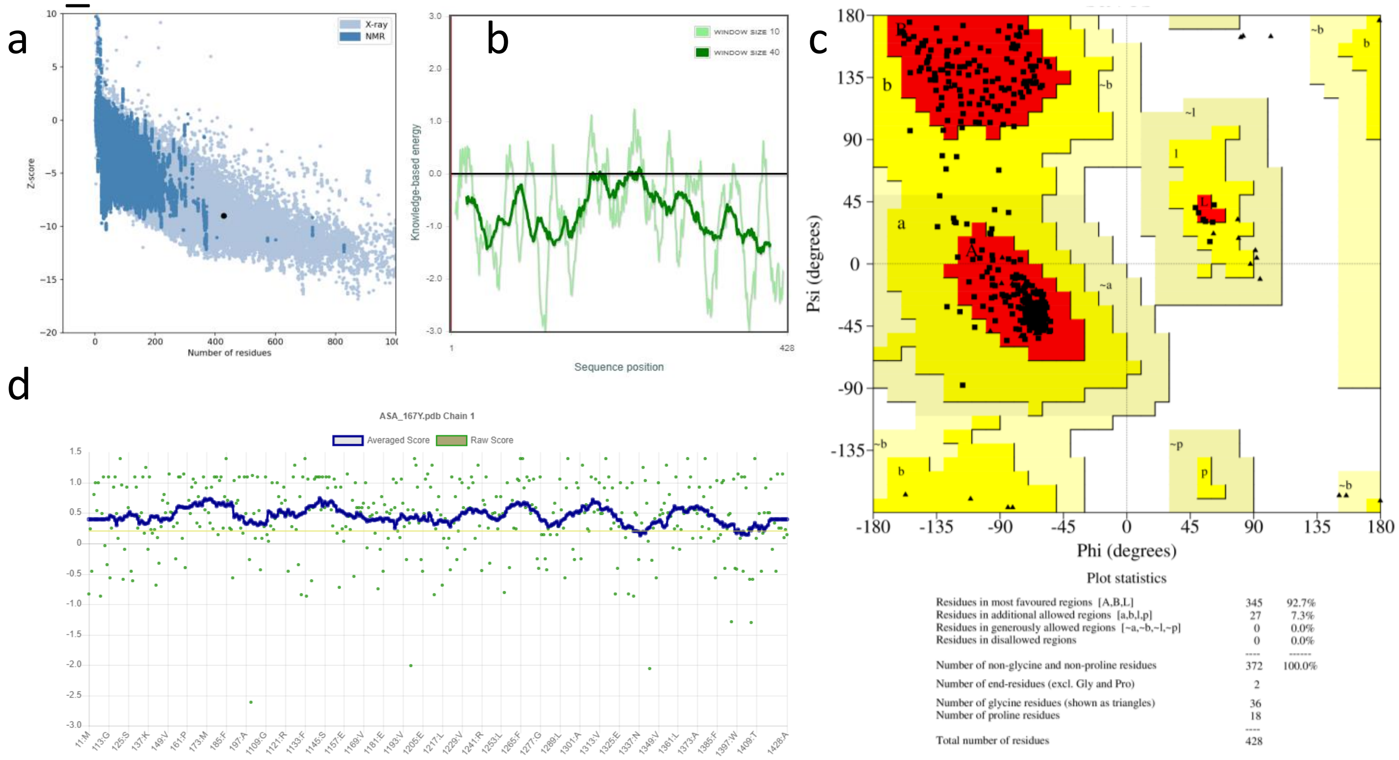

Supplementary Figure S3 – Quality checks on mutated *Ascaris suum* F167Y isotype A minimized protein structure. a) ProSA-web global Z-score compared to all other proteins in the PDB database. The black dot shows the Z-score of the modelled protein. Proteins with Z-scores within the range of those from the PDB database show a protein with realistic structure. b) ProSA-web local model energies averaged over sliding windows of 10 and 40 residues. Positive values indicate potential erroneous regions. c) PROCHECK Ramachandran plot shows the torsion angles of each residue predicting whether they are in possible combinations. d) Verify3D results which compares the 3D structure to the amino acid sequence. Results more than 0.2 indicate that the structure of the protein matches what would be predicted from the amino acid sequence. Proteins with more than 80% of residues above the 0.2 threshold pass the criteria. All tests suggest that the minimized homology model is suitable for analysis.

ASA_198

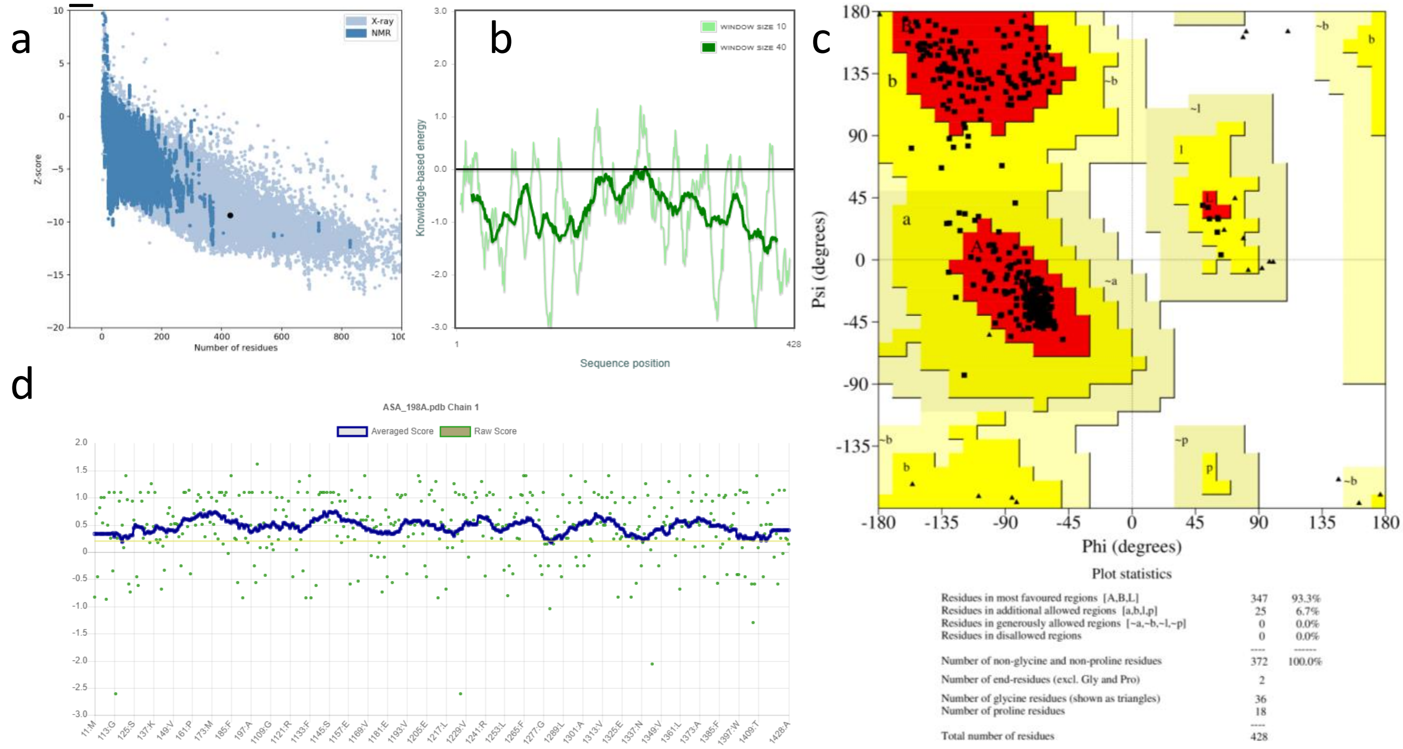

Supplementary Figure S4 – Quality checks on mutated *Ascaris suum* E198A isotype A minimized protein structure. a) ProSA-web global Z-score compared to all other proteins in the PDB database. The black dot shows the Z-score of the modelled protein. Proteins with Z-scores within the range of those from the PDB database show a protein with realistic structure. b) ProSA-web local model energies averaged over sliding windows of 10 and 40 residues. Positive values indicate potential erroneous regions. c) PROCHECK Ramachandran plot shows the torsion angles of each residue predicting whether they are in possible combinations. d) Verify3D results which compares the 3D structure to the amino acid sequence. Results more than 0.2 indicate that the structure of the protein matches what would be predicted from the amino acid sequence. Proteins with more than 80% of residues above the 0.2 threshold pass the criteria. All tests suggest that the minimized homology model is suitable for analysis.

ASA_200

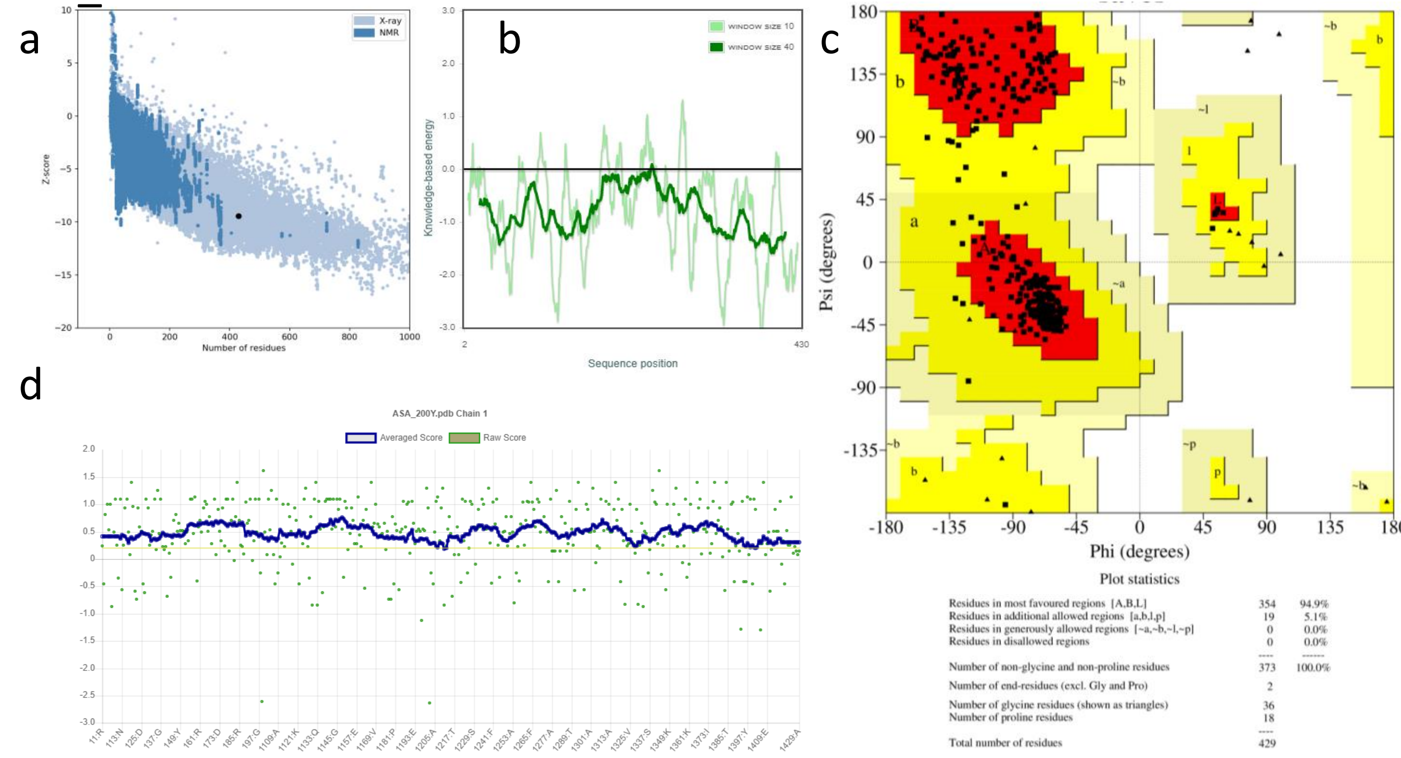

Supplementary Figure S5 – Quality checks on mutated *Ascaris suum* F200Y isotype A minimized protein structure. a) ProSA-web global Z-score compared to all other proteins in the PDB database. The black dot shows the Z-score of the modelled protein. Proteins with Z-scores within the range of those from the PDB database show a protein with realistic structure. b) ProSA-web local model energies averaged over sliding windows of 10 and 40 residues. Positive values indicate potential erroneous regions. c) PROCHECK Ramachandran plot shows the torsion angles of each residue predicting whether they are in possible combinations. d) Verify3D results which compares the 3D structure to the amino acid sequence. Results more than 0.2 indicate that the structure of the protein matches what would be predicted from the amino acid sequence. Proteins with more than 80% of residues above the 0.2 threshold pass the criteria. All tests suggest that the minimized homology model is suitable for analysis.

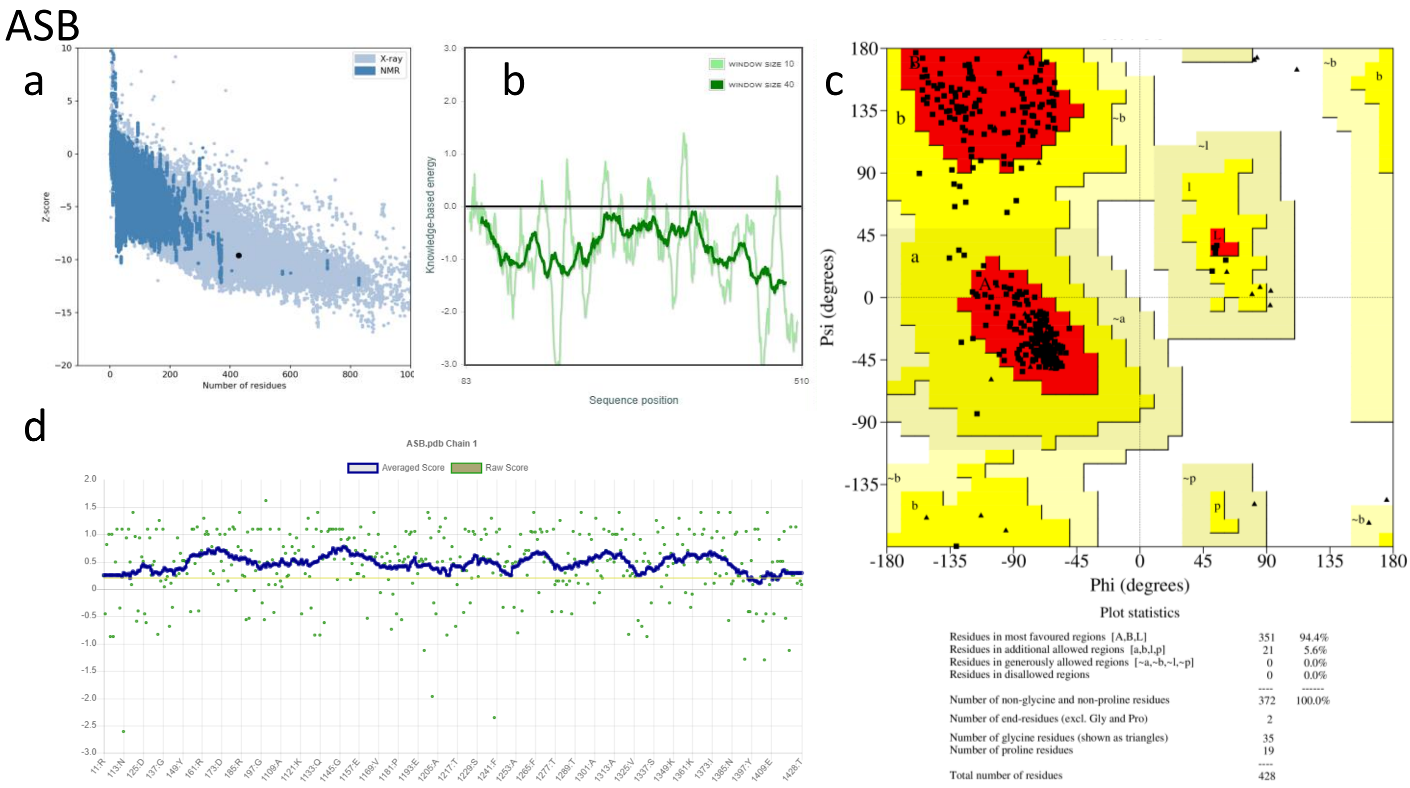

Supplementary Figure S6 – Quality checks on *Ascaris suum* isotype B minimized protein structure. a) ProSA-web global Z-score compared to all other proteins in the PDB database. The black dot shows the Z-score of the modelled protein. Proteins with Z-scores within the range of those from the PDB database show a protein with realistic structure. b) ProSA-web local model energies averaged over sliding windows of 10 and 40 residues. Positive values indicate potential erroneous regions. c) PROCHECK Ramachandran plot shows the torsion angles of each residue predicting whether they are in possible combinations. d) Verify3D results which compares the 3D structure to the amino acid sequence. Results more than 0.2 indicate that the structure of the protein matches what would be predicted from the amino acid sequence. Proteins with more than 80% of residues above the 0.2 threshold pass the criteria. All tests suggest that the minimized homology model is suitable for analysis.

ASC

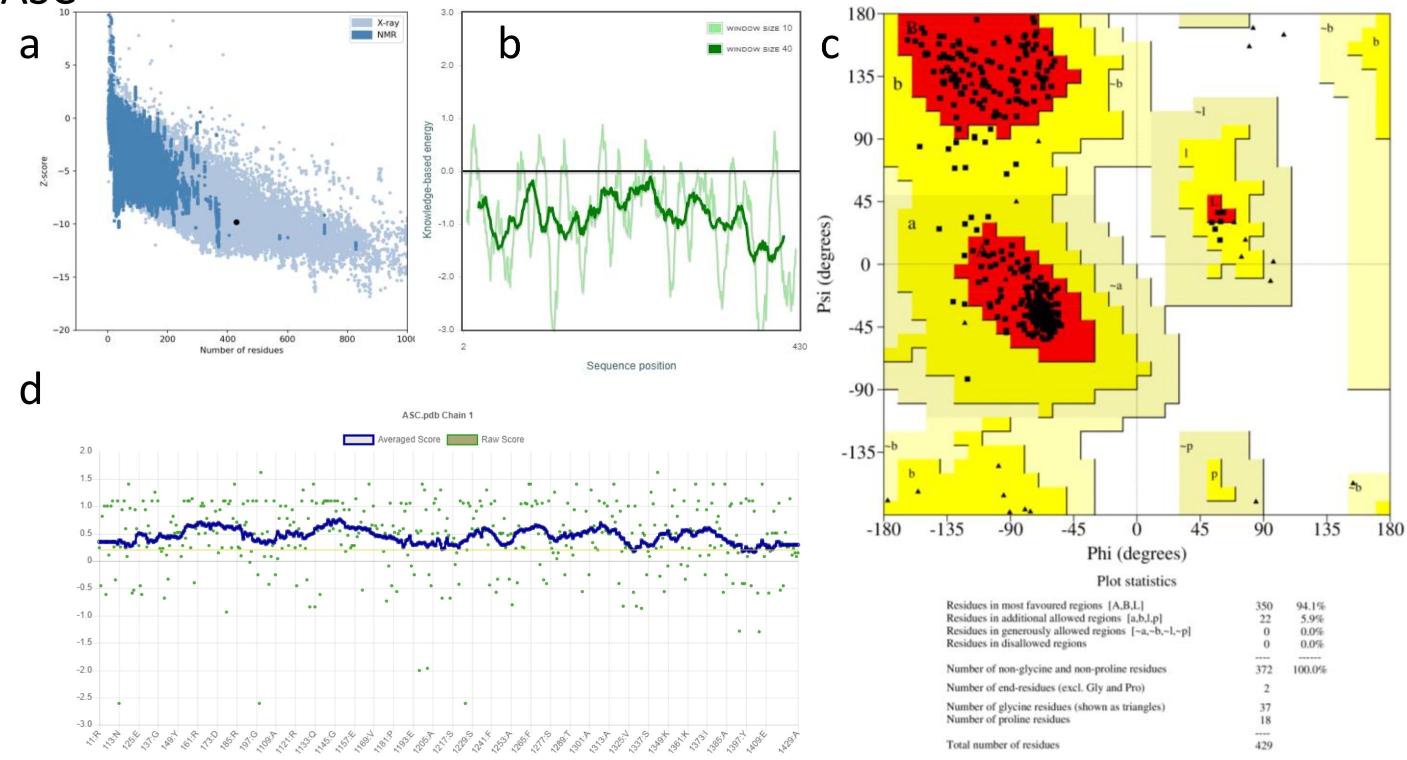

Supplementary Figure S7 – Quality checks on *Ascaris suum* isotype C minimized protein structure. a) ProSA-web global Z-score compared to all other proteins in the PDB database. The black dot shows the Z-score of the modelled protein. Proteins with Z-scores within the range of those from the PDB database show a protein with realistic structure. b) ProSA-web local model energies averaged over sliding windows of 10 and 40 residues. Positive values indicate potential erroneous regions. c) PROCHECK Ramachandran plot shows the torsion angles of each residue predicting whether they are in possible combinations. d) Verify3D results which compares the 3D structure to the amino acid sequence. Results more than 0.2 indicate that the structure of the protein matches what would be predicted from the amino acid sequence. Proteins with more than 80% of residues above the 0.2 threshold pass the criteria. All tests suggest that the minimized homology model is suitable for analysis.

ASD

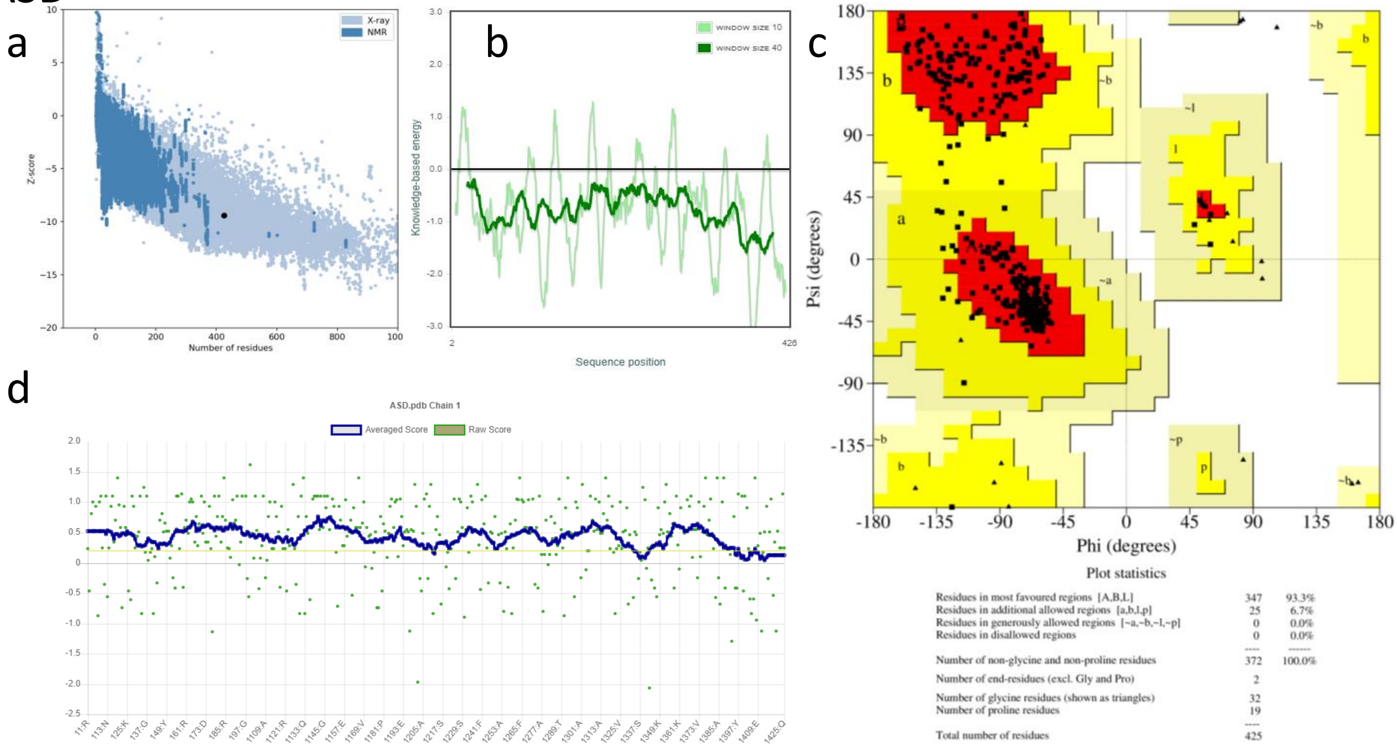

Supplementary Figure S8 – Quality checks on *Ascaris suum* isotype D minimized protein structure. a) ProSA-web global Z-score compared to all other proteins in the PDB database. The black dot shows the Z-score of the modelled protein. Proteins with Z-scores within the range of those from the PDB database show a protein with realistic structure. b) ProSA-web local model energies averaged over sliding windows of 10 and 40 residues. Positive values indicate potential erroneous regions. c) PROCHECK Ramachandran plot shows the torsion angles of each residue predicting whether they are in possible combinations. d) Verify3D results which compares the 3D structure to the amino acid sequence. Results more than 0.2 indicate that the structure of the protein matches what would be predicted from the amino acid sequence. Proteins with more than 80% of residues above the 0.2 threshold pass the criteria. All tests suggest that the minimized homology model is suitable for analysis.

ASE

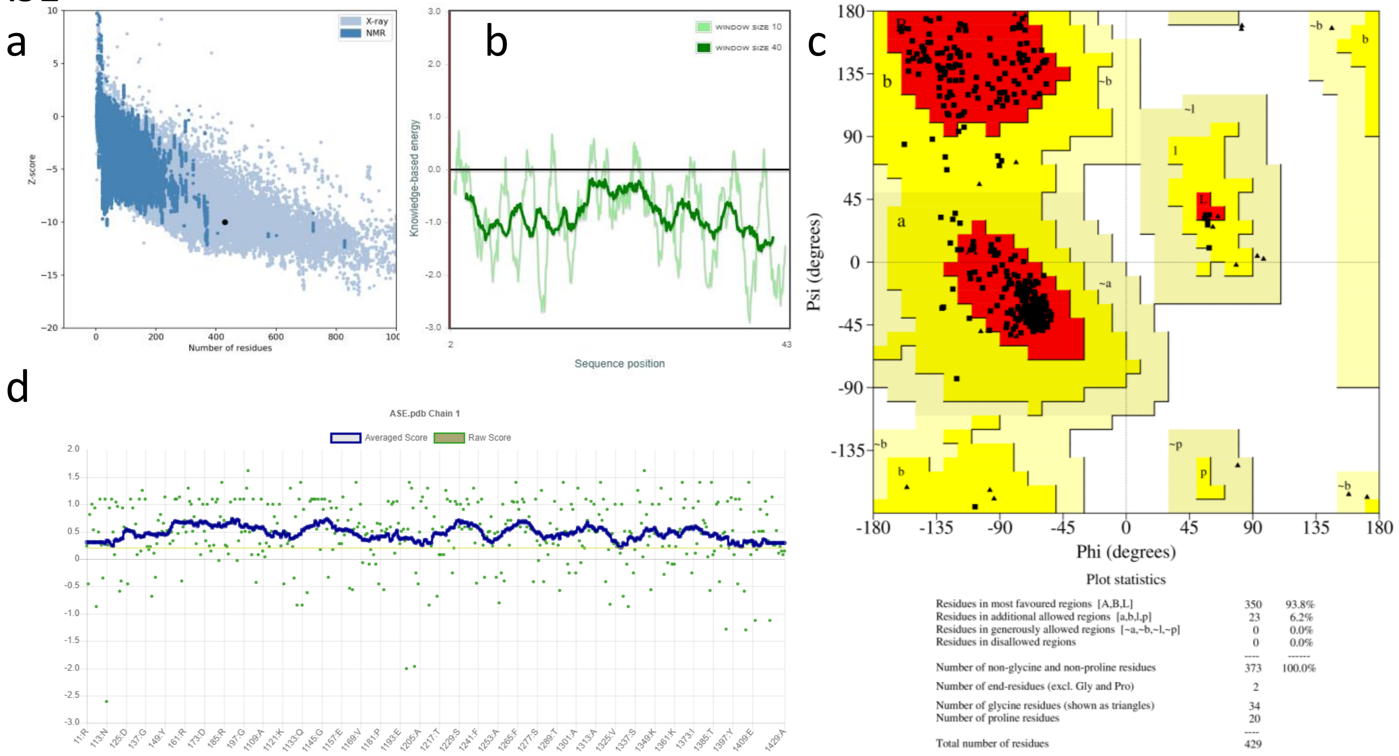

Supplementary Figure S9 – Quality checks on *Ascaris suum* isotype E minimized protein structure. a) ProSA-web global Z-score compared to all other proteins in the PDB database. The black dot shows the Z-score of the modelled protein. Proteins with Z-scores within the range of those from the PDB database show a protein with realistic structure. b) ProSA-web local model energies averaged over sliding windows of 10 and 40 residues. Positive values indicate potential erroneous regions. c) PROCHECK Ramachandran plot shows the torsion angles of each residue predicting whether they are in possible combinations. d) Verify3D results which compares the 3D structure to the amino acid sequence. Results more than 0.2 indicate that the structure of the protein matches what would be predicted from the amino acid sequence. Proteins with more than 80% of residues above the 0.2 threshold pass the criteria. All tests suggest that the minimized homology model is suitable for analysis.

ASF

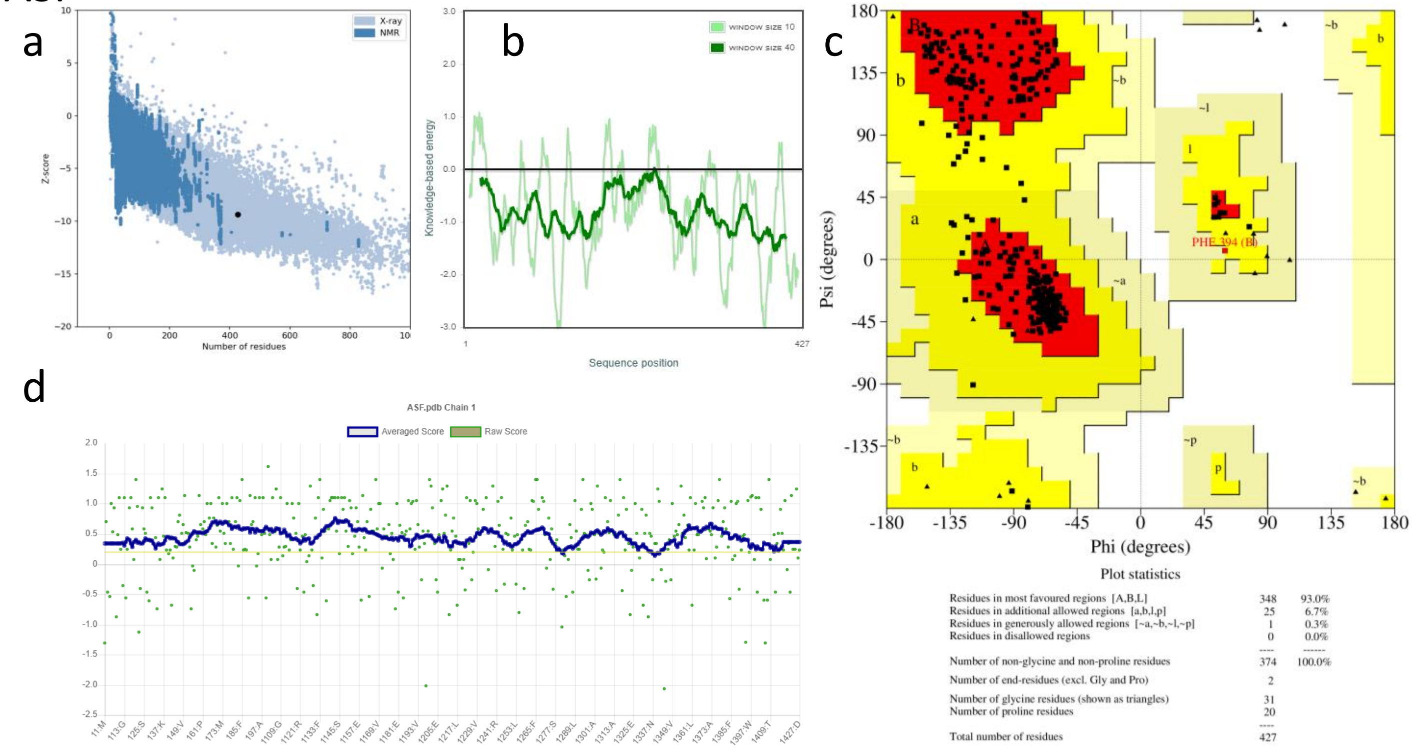

Supplementary Figure S10 – Quality checks on *Ascaris suum* isotype F minimized protein structure. a) ProSA-web global Z-score compared to all other proteins in the PDB database. The black dot shows the Z-score of the modelled protein. Proteins with Z-scores within the range of those from the PDB database show a protein with realistic structure. b) ProSA-web local model energies averaged over sliding windows of 10 and 40 residues. Positive values indicate potential erroneous regions. c) PROCHECK Ramachandran plot shows the torsion angles of each residue predicting whether they are in possible combinations. d) Verify3D results which compares the 3D structure to the amino acid sequence. Results more than 0.2 indicate that the structure of the protein matches what would be predicted from the amino acid sequence. Proteins with more than 80% of residues above the 0.2 threshold pass the criteria. All tests suggest that the minimized homology model is suitable for analysis.

ASG

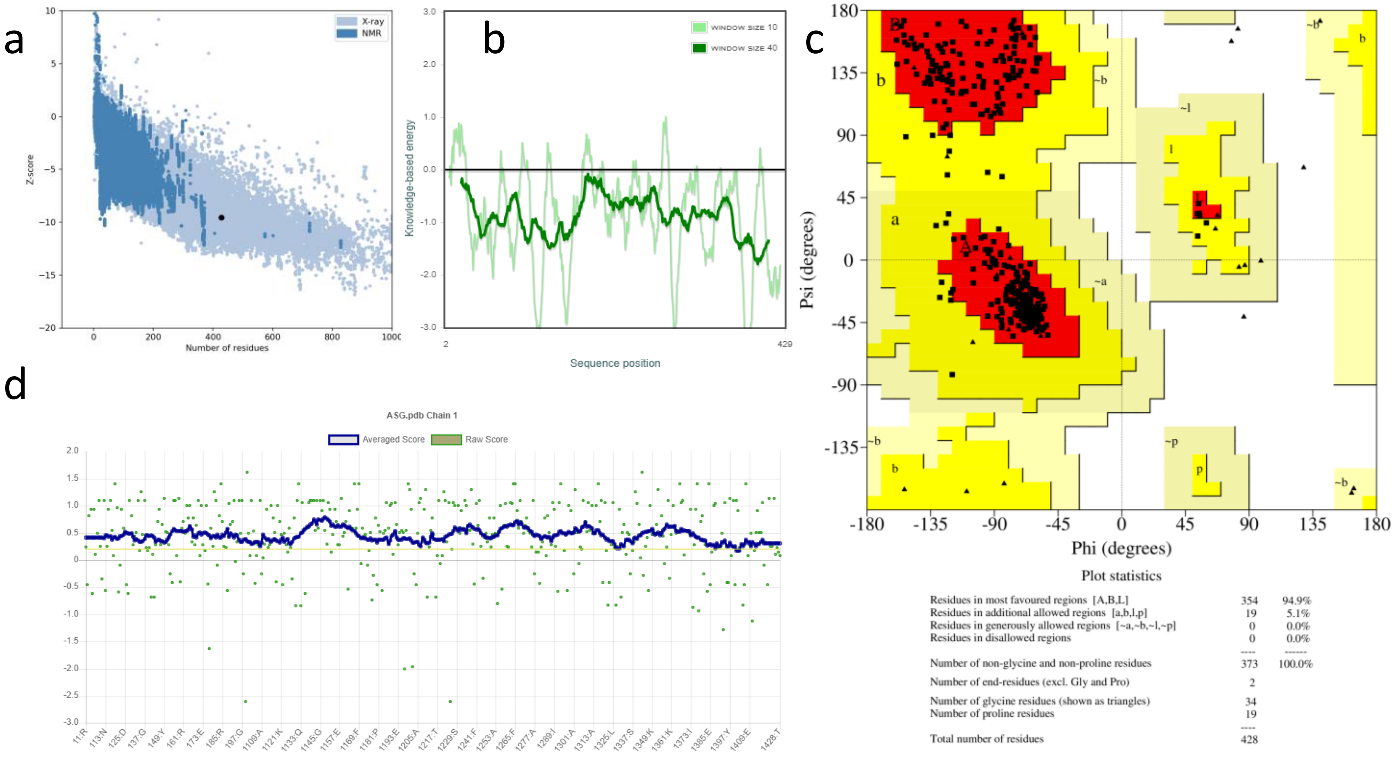

Supplementary Figure S11 – Quality checks on *Ascaris suum* isotype G minimized protein structure. a) ProSA-web global Z-score compared to all other proteins in the PDB database. The black dot shows the Z-score of the modelled protein. Proteins with Z-scores within the range of those from the PDB database show a protein with realistic structure. b) ProSA-web local model energies averaged over sliding windows of 10 and 40 residues. Positive values indicate potential erroneous regions. c) PROCHECK Ramachandran plot shows the torsion angles of each residue predicting whether they are in possible combinations. d) Verify3D results which compares the 3D structure to the amino acid sequence. Results more than 0.2 indicate that the structure of the protein matches what would be predicted from the amino acid sequence. Proteins with more than 80% of residues above the 0.2 threshold pass the criteria. All tests suggest that the minimized homology model is suitable for analysis.

ALA

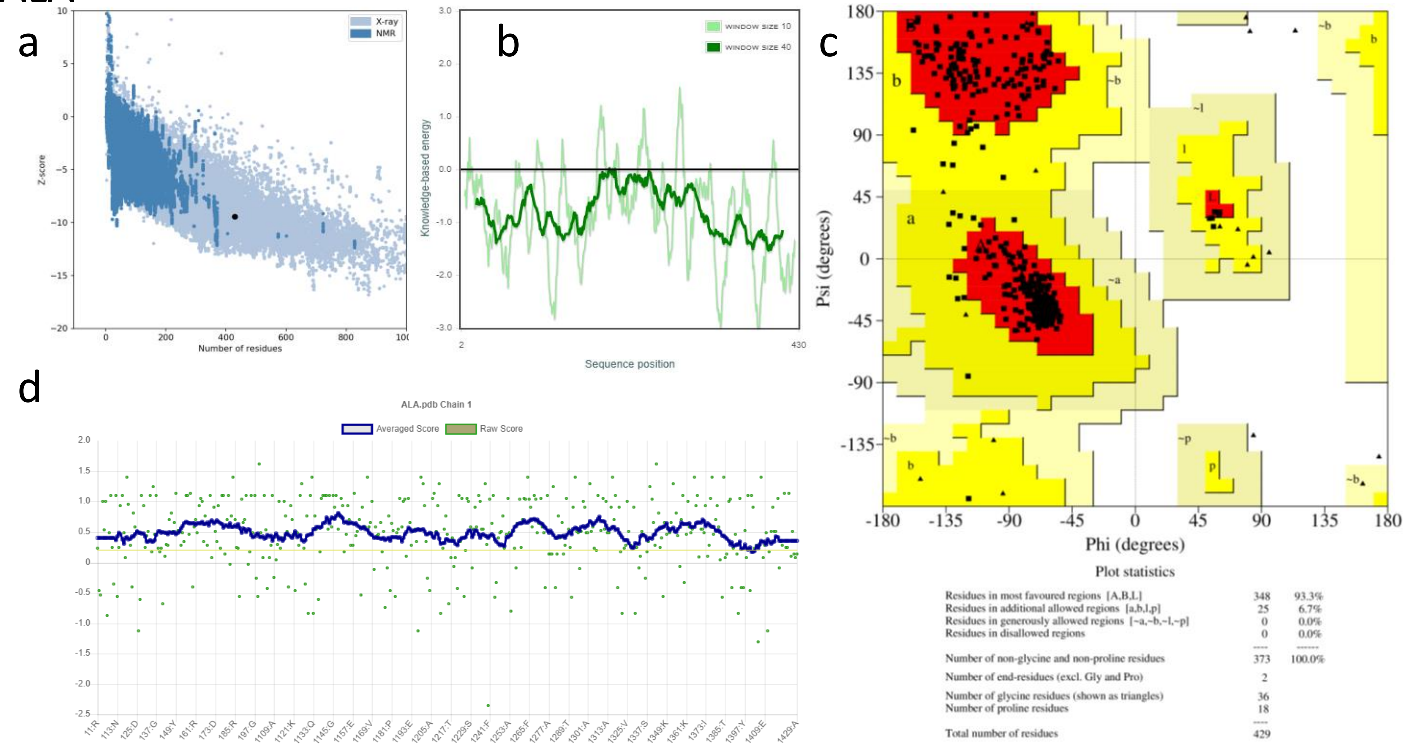

Supplementary Figure S12 – Quality checks on *Ascaris lumbricoides* isotype A minimized protein structure. a) ProSA-web global Z-score compared to all other proteins in the PDB database. The black dot shows the Z-score of the modelled protein. Proteins with Z-scores within the range of those from the PDB database show a protein with realistic structure. b) ProSA-web local model energies averaged over sliding windows of 10 and 40 residues. Positive values indicate potential erroneous regions. c) PROCHECK Ramachandran plot shows the torsion angles of each residue predicting whether they are in possible combinations. d) Verify3D results which compares the 3D structure to the amino acid sequence. Results more than 0.2 indicate that the structure of the protein matches what would be predicted from the amino acid sequence. Proteins with more than 80% of residues above the 0.2 threshold pass the criteria. All tests suggest that the minimized homology model is suitable for analysis.

ALA_F167Y

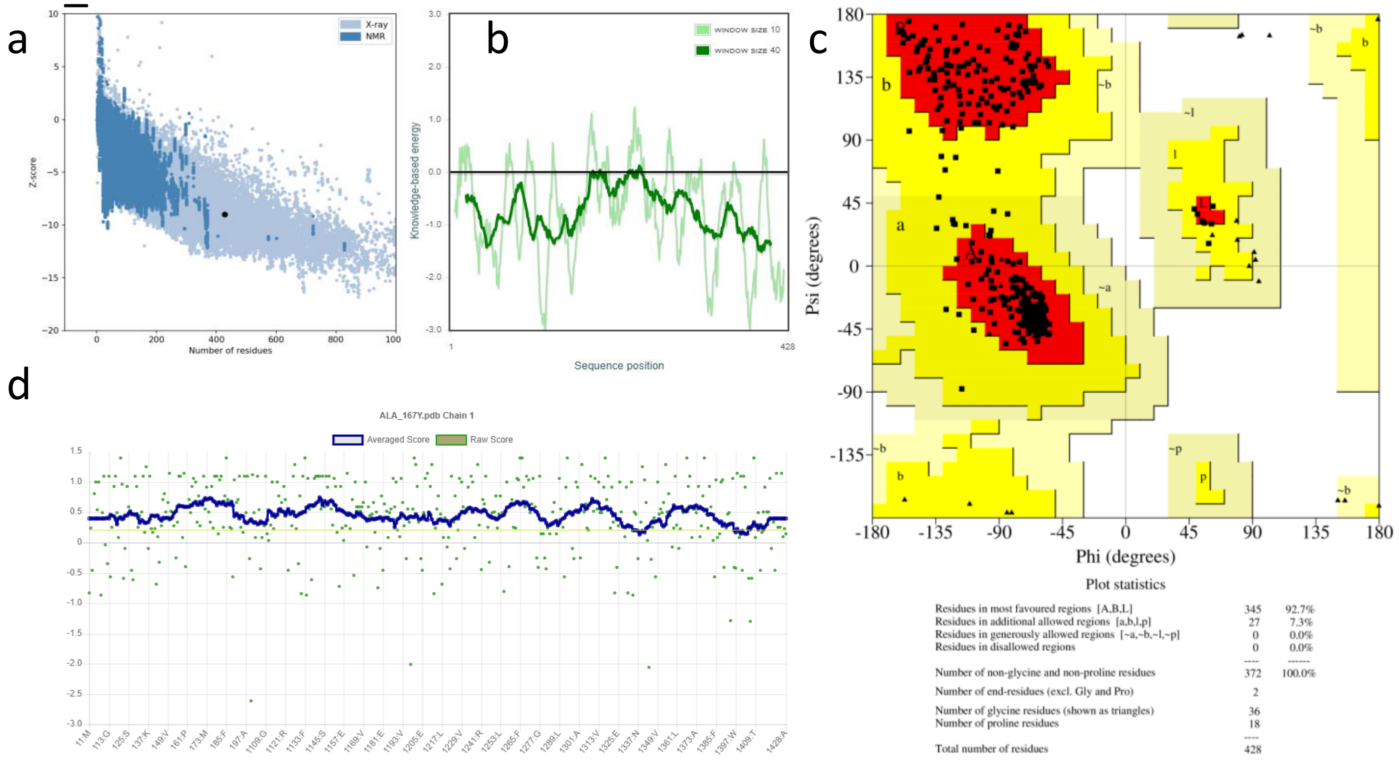

Supplementary Figure S13 – Quality checks on mutated *Ascaris lumbricoides* F167Y isotype A minimized protein structure. a) ProSA-web global Z-score compared to all other proteins in the PDB database. The black dot shows the Z-score of the modelled protein. Proteins with Z-scores within the range of those from the PDB database show a protein with realistic structure. b) ProSA-web local model energies averaged over sliding windows of 10 and 40 residues. Positive values indicate potential erroneous regions. c) PROCHECK Ramachandran plot shows the torsion angles of each residue predicting whether they are in possible combinations. d) Verify3D results which compares the 3D structure to the amino acid sequence. Results more than 0.2 indicate that the structure of the protein matches what would be predicted from the amino acid sequence. Proteins with more than 80% of residues above the 0.2 threshold pass the criteria. All tests suggest that the minimized homology model is suitable for analysis.

ALA_E198A

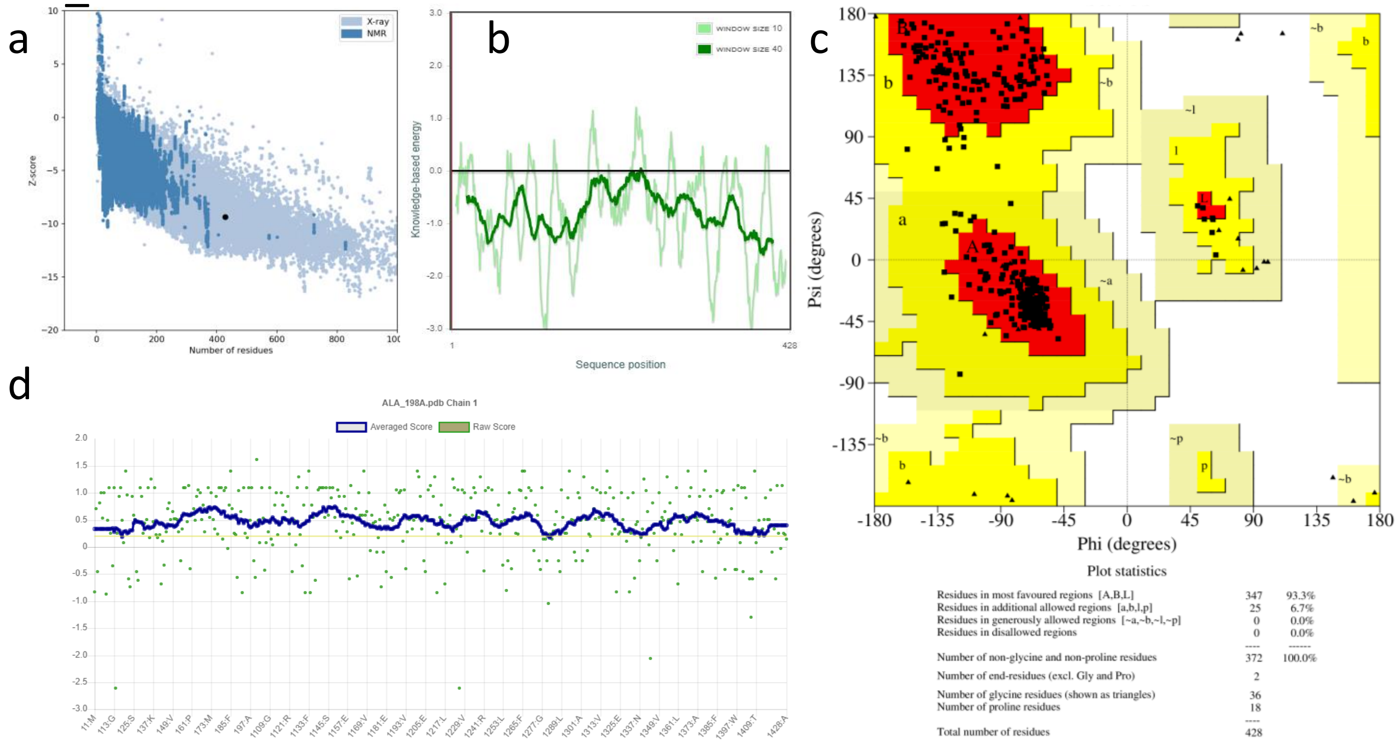

Supplementary Figure S14 – Quality checks on mutated *Ascaris lumbricoides* E198A isotype A minimized protein structure. a) ProSA-web global Z-score compared to all other proteins in the PDB database. The black dot shows the Z-score of the modelled protein. Proteins with Z-scores within the range of those from the PDB database show a protein with realistic structure. b) ProSA-web local model energies averaged over sliding windows of 10 and 40 residues. Positive values indicate potential erroneous regions. c) PROCHECK Ramachandran plot shows the torsion angles of each residue predicting whether they are in possible combinations. d) Verify3D results which compares the 3D structure to the amino acid sequence. Results more than 0.2 indicate that the structure of the protein matches what would be predicted from the amino acid sequence. Proteins with more than 80% of residues above the 0.2 threshold pass the criteria. All tests suggest that the minimized homology model is suitable for analysis.

ALA_F200Y

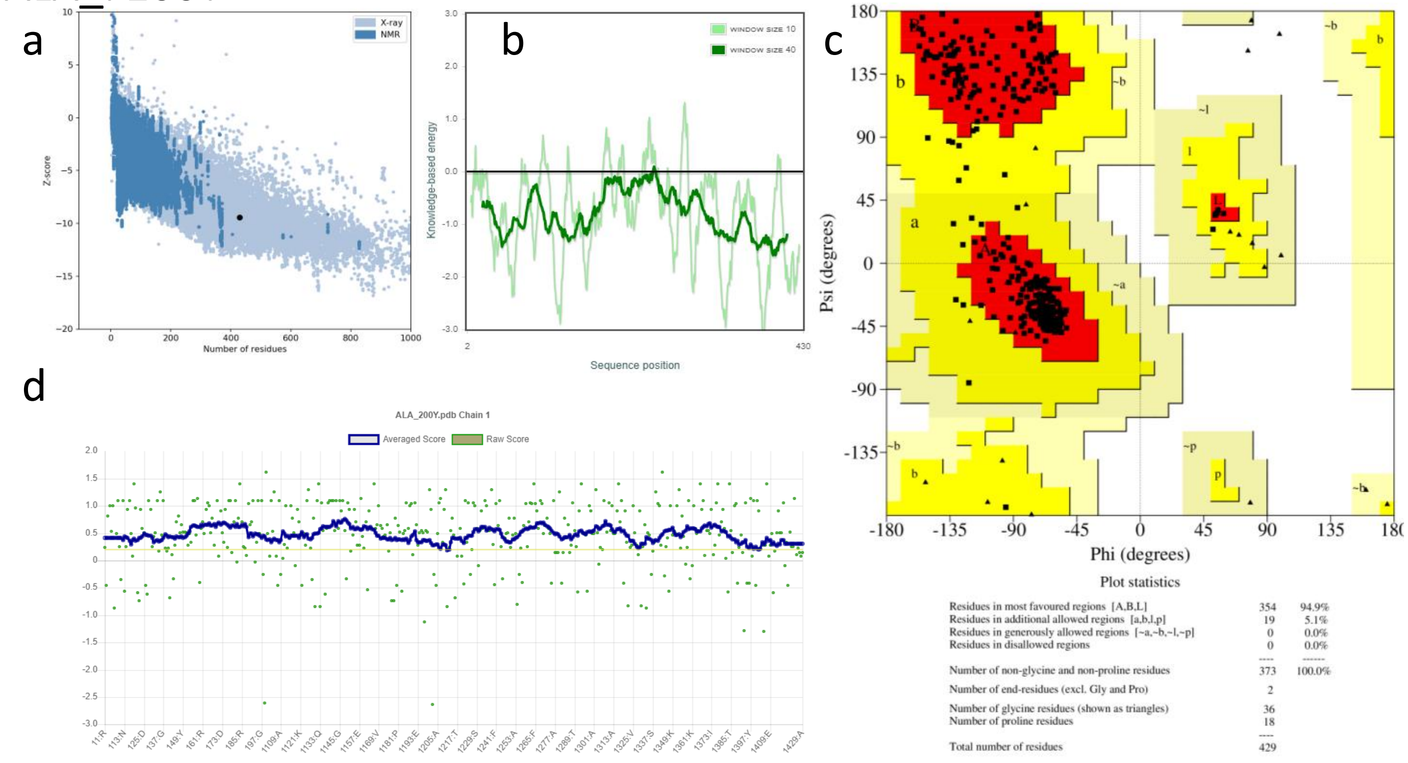

Supplementary Figure S15 – Quality checks on mutated *Ascaris lumbricoides* F200Y isotype A minimized protein structure. a) ProSA-web global Z-score compared to all other proteins in the PDB database. The black dot shows the Z-score of the modelled protein. Proteins with Z-scores within the range of those from the PDB database show a protein with realistic structure. b) ProSA-web local model energies averaged over sliding windows of 10 and 40 residues. Positive values indicate potential erroneous regions. c) PROCHECK Ramachandran plot shows the torsion angles of each residue predicting whether they are in possible combinations. d) Verify3D results which compares the 3D structure to the amino acid sequence. Results more than 0.2 indicate that the structure of the protein matches what would be predicted from the amino acid sequence. Proteins with more than 80% of residues above the 0.2 threshold pass the criteria. All tests suggest that the minimized homology model is suitable for analysis.

ALB

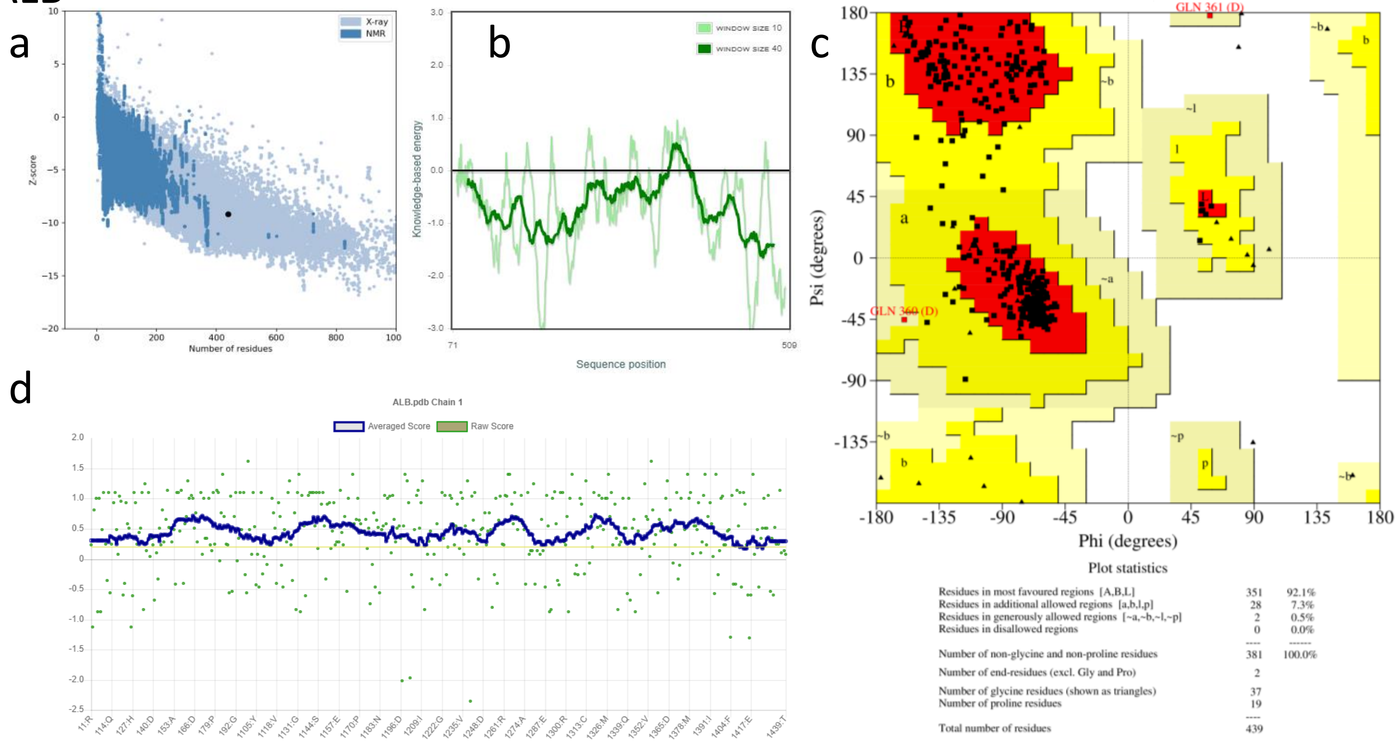

Supplementary Figure S16 – Quality checks on *Ascaris lumbricoides* isotype B minimized protein structure. a) ProSA-web global Z-score compared to all other proteins in the PDB database. The black dot shows the Z-score of the modelled protein. Proteins with Z-scores within the range of those from the PDB database show a protein with realistic structure. b) ProSA-web local model energies averaged over sliding windows of 10 and 40 residues. Positive values indicate potential erroneous regions. c) PROCHECK Ramachandran plot shows the torsion angles of each residue predicting whether they are in possible combinations. d) Verify3D results which compares the 3D structure to the amino acid sequence. Results more than 0.2 indicate that the structure of the protein matches what would be predicted from the amino acid sequence. Proteins with more than 80% of residues above the 0.2 threshold pass the criteria. All tests suggest that the minimized homology model is suitable for analysis.

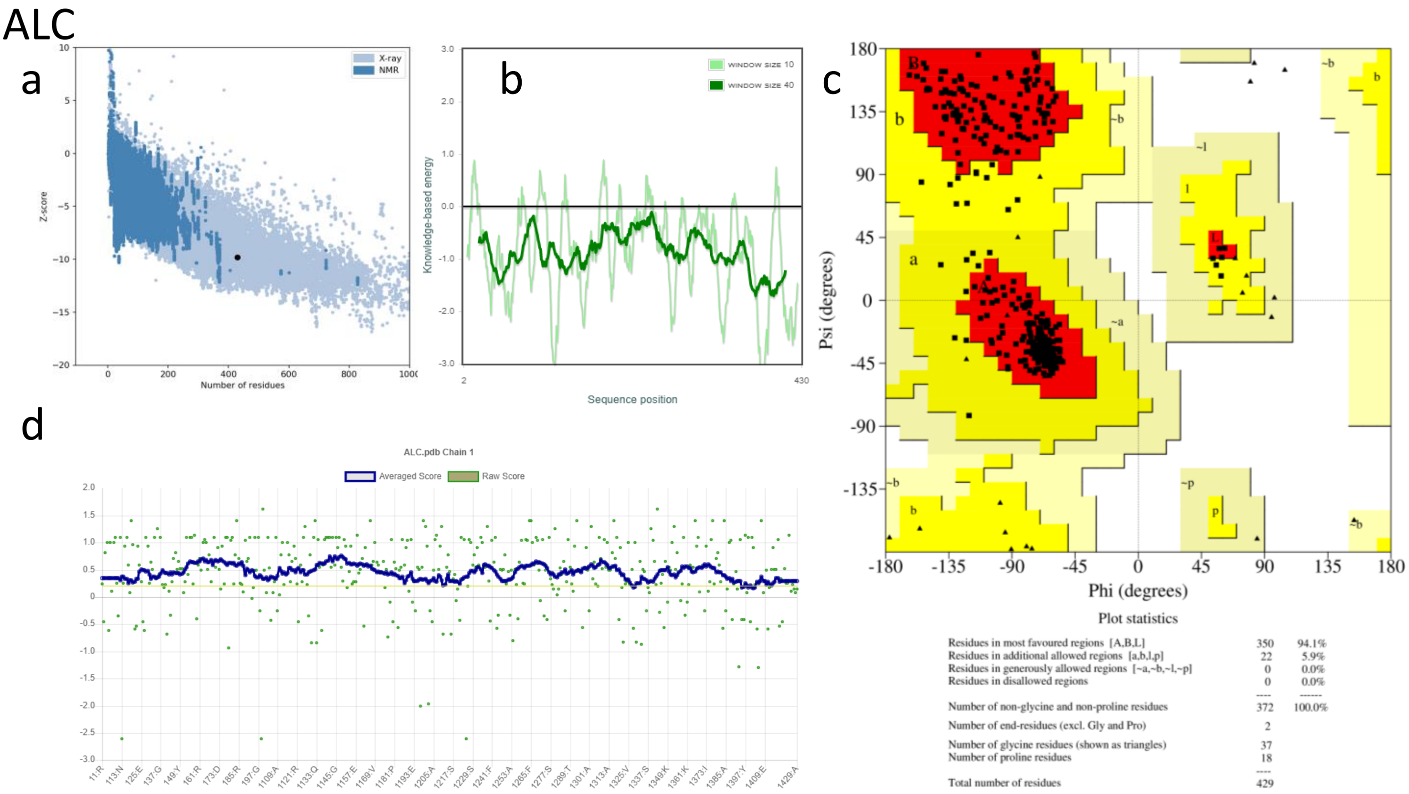

Supplementary Figure S17 – Quality checks on *Ascaris lumbricoides* isotype C minimized protein structure. a) ProSA-web global Z-score compared to all other proteins in the PDB database. The black dot shows the Z-score of the modelled protein. Proteins with Z-scores within the range of those from the PDB database show a protein with realistic structure. b) ProSA-web local model energies averaged over sliding windows of 10 and 40 residues. Positive values indicate potential erroneous regions. c) PROCHECK Ramachandran plot shows the torsion angles of each residue predicting whether they are in possible combinations. d) Verify3D results which compares the 3D structure to the amino acid sequence. Results more than 0.2 indicate that the structure of the protein matches what would be predicted from the amino acid sequence. Proteins with more than 80% of residues above the 0.2 threshold pass the criteria. All tests suggest that the minimized homology model is suitable for analysis.

ALD

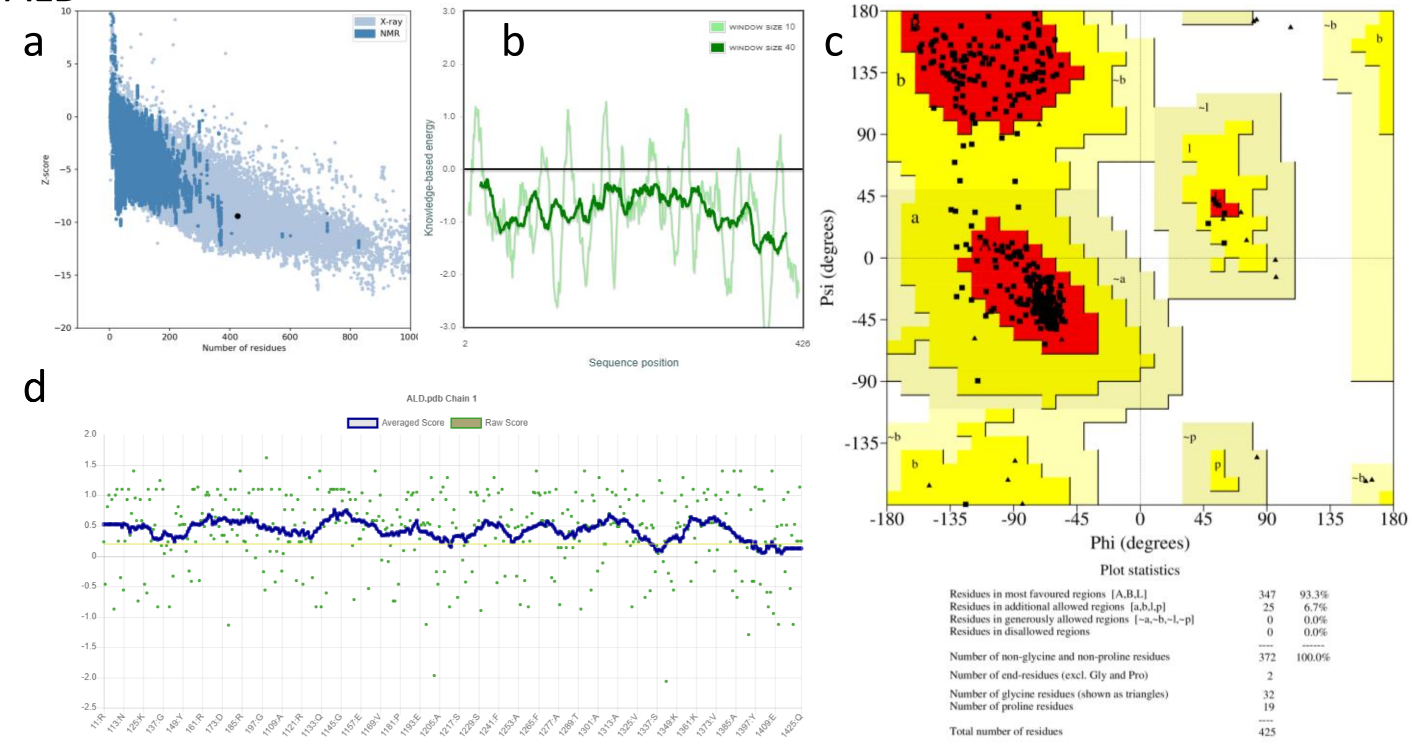

Supplementary Figure S18 – Quality checks on *Ascaris lumbricoides* isotype D minimized protein structure. a) ProSA-web global Z-score compared to all other proteins in the PDB database. The black dot shows the Z-score of the modelled protein. Proteins with Z-scores within the range of those from the PDB database show a protein with realistic structure. b) ProSA-web local model energies averaged over sliding windows of 10 and 40 residues. Positive values indicate potential erroneous regions. c) PROCHECK Ramachandran plot shows the torsion angles of each residue predicting whether they are in possible combinations. d) Verify3D results which compares the 3D structure to the amino acid sequence. Results more than 0.2 indicate that the structure of the protein matches what would be predicted from the amino acid sequence. Proteins with more than 80% of residues above the 0.2 threshold pass the criteria. All tests suggest that the minimized homology model is suitable for analysis.

ALE

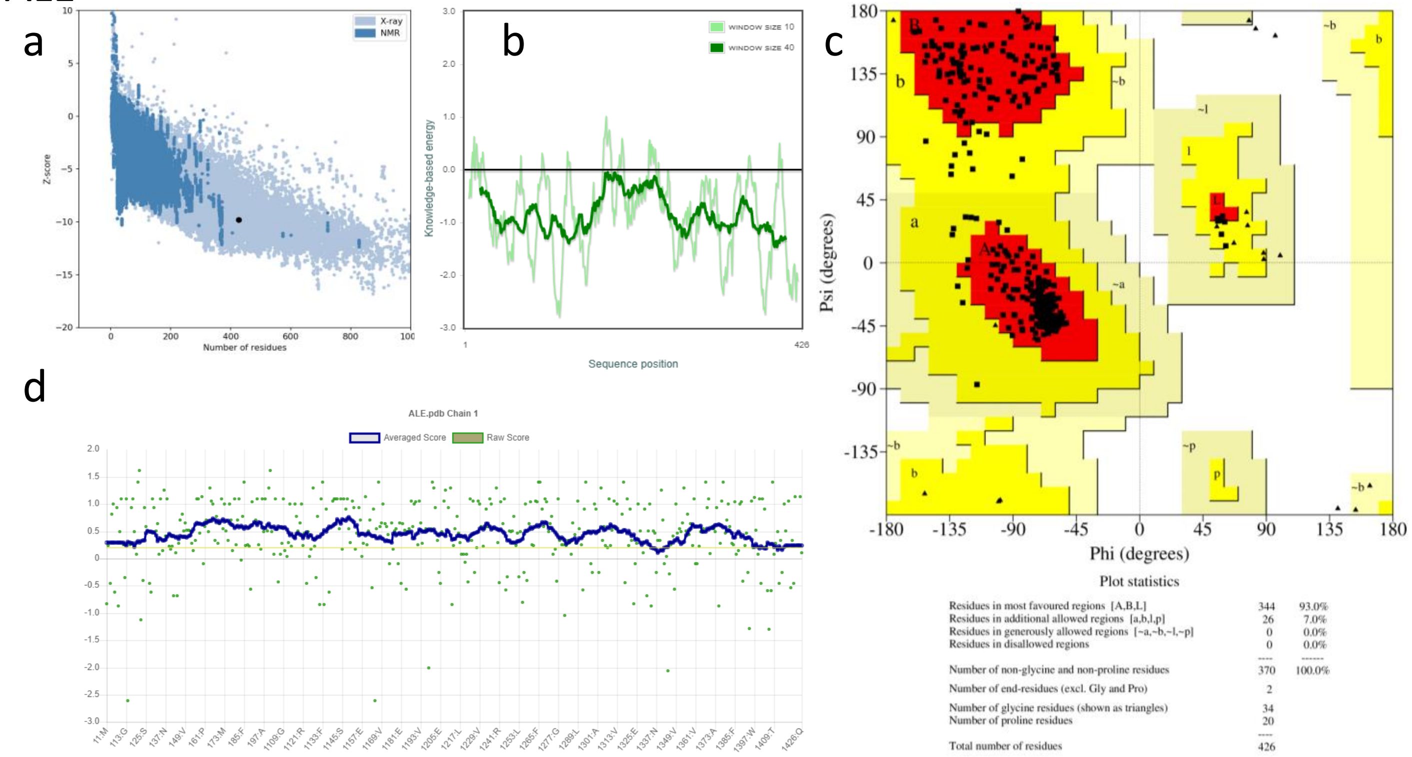

Supplementary Figure S19 – Quality checks on *Ascaris lumbricoides* isotype E minimized protein structure. a) ProSA-web global Z-score compared to all other proteins in the PDB database. The black dot shows the Z-score of the modelled protein. Proteins with Z-scores within the range of those from the PDB database show a protein with realistic structure. b) ProSA-web local model energies averaged over sliding windows of 10 and 40 residues. Positive values indicate potential erroneous regions. c) PROCHECK Ramachandran plot shows the torsion angles of each residue predicting whether they are in possible combinations. d) Verify3D results which compares the 3D structure to the amino acid sequence. Results more than 0.2 indicate that the structure of the protein matches what would be predicted from the amino acid sequence. Proteins with more than 80% of residues above the 0.2 threshold pass the criteria. All tests suggest that the minimized homology model is suitable for analysis.

ALF

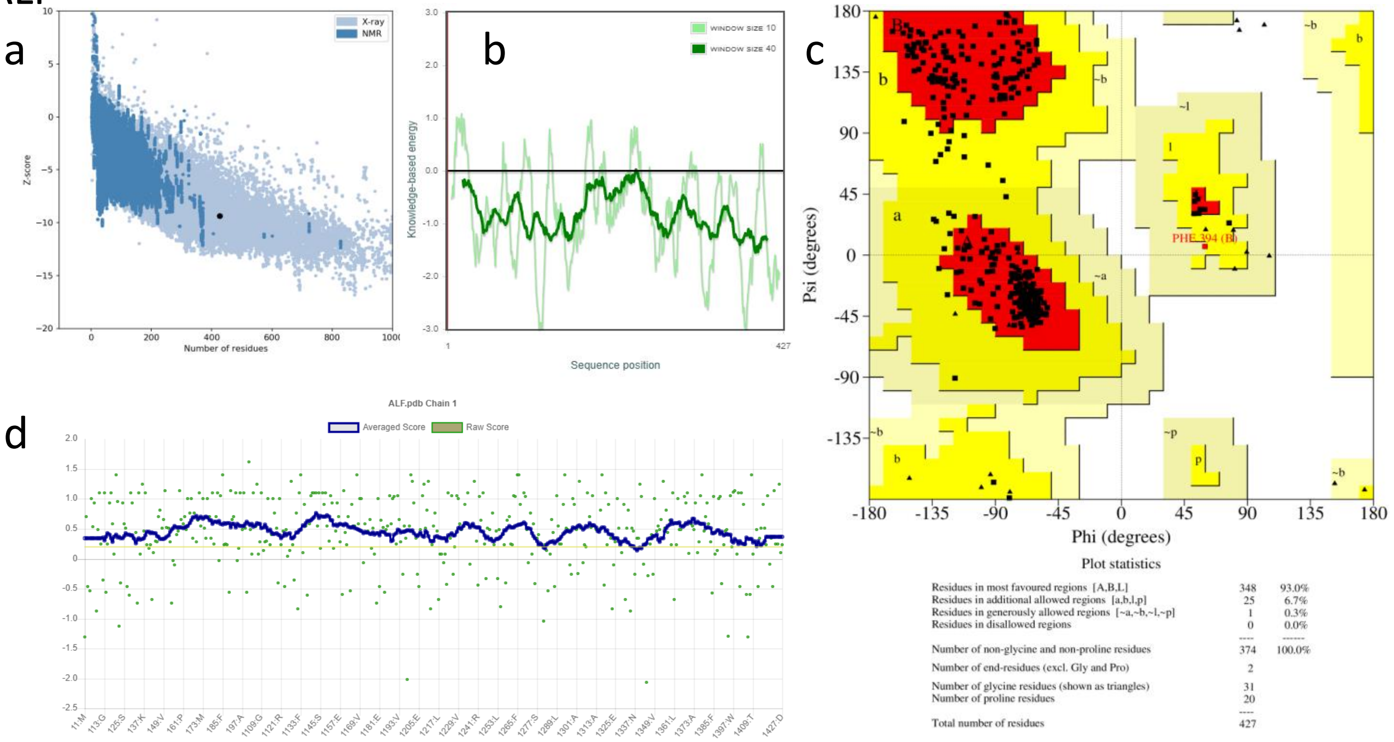

Supplementary Figure S20 – Quality checks on *Ascaris lumbricoides* isotype F minimized protein structure. a) ProSA-web global Z-score compared to all other proteins in the PDB database. The black dot shows the Z-score of the modelled protein. Proteins with Z-scores within the range of those from the PDB database show a protein with realistic structure. b) ProSA-web local model energies averaged over sliding windows of 10 and 40 residues. Positive values indicate potential erroneous regions. c) PROCHECK Ramachandran plot shows the torsion angles of each residue predicting whether they are in possible combinations. d) Verify3D results which compares the 3D structure to the amino acid sequence. Results more than 0.2 indicate that the structure of the protein matches what would be predicted from the amino acid sequence. Proteins with more than 80% of residues above the 0.2 threshold pass the criteria. All tests suggest that the minimized homology model is suitable for analysis.

ALG

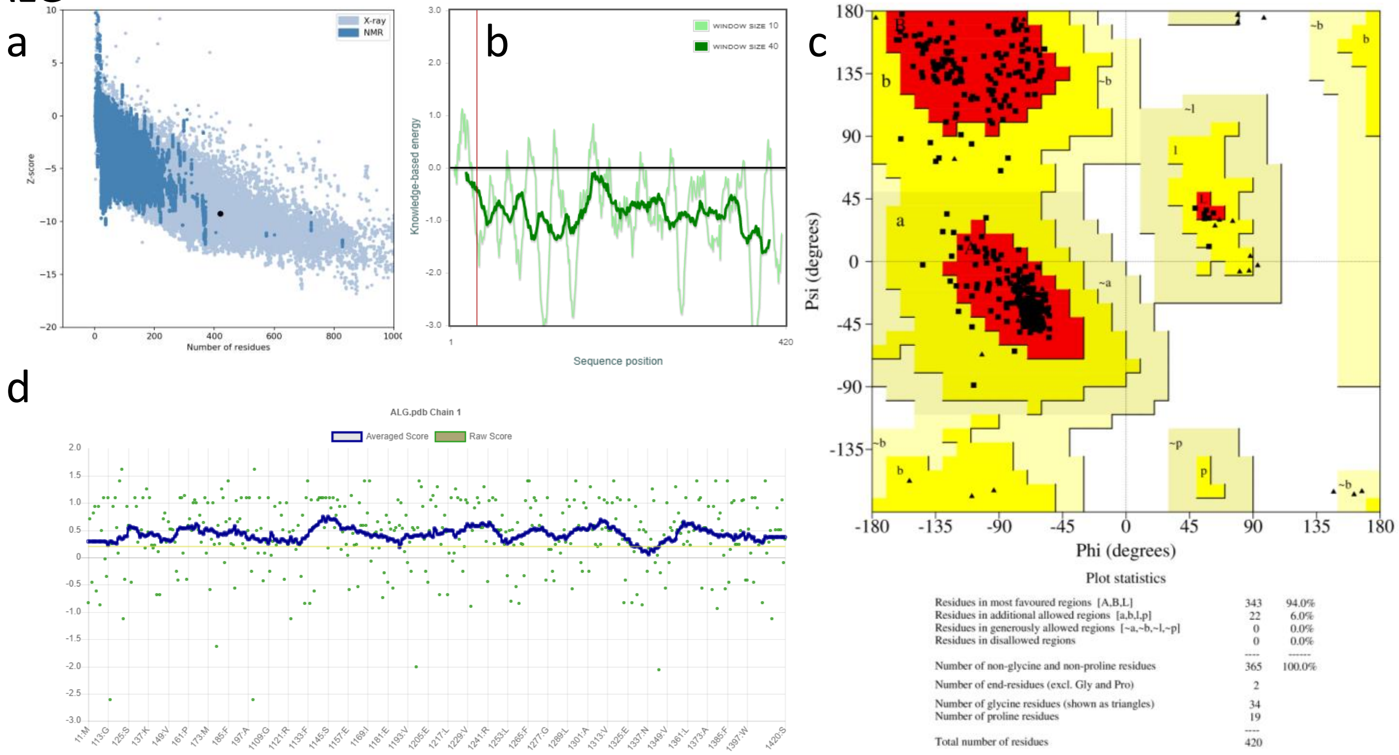

Supplementary Figure S21 – Quality checks on *Ascaris lumbricoides* isotype G minimized protein structure. a) ProSA-web global Z-score compared to all other proteins in the PDB database. The black dot shows the Z-score of the modelled protein. Proteins with Z-scores within the range of those from the PDB database show a protein with realistic structure. b) ProSA-web local model energies averaged over sliding windows of 10 and 40 residues. Positive values indicate potential erroneous regions. c) PROCHECK Ramachandran plot shows the torsion angles of each residue predicting whether they are in possible combinations. d) Verify3D results which compares the 3D structure to the amino acid sequence. Results more than 0.2 indicate that the structure of the protein matches what would be predicted from the amino acid sequence. Proteins with more than 80% of residues above the 0.2 threshold pass the criteria. All tests suggest that the minimized homology model is suitable for analysis.

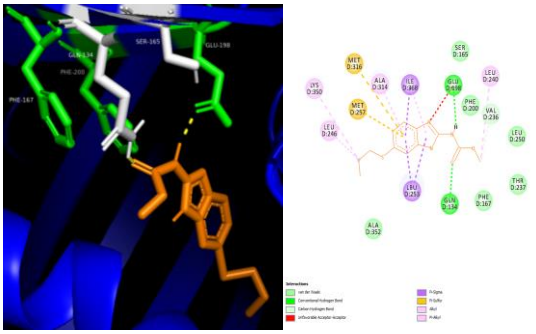

ASA-ABZ -5.8 kcal/mol

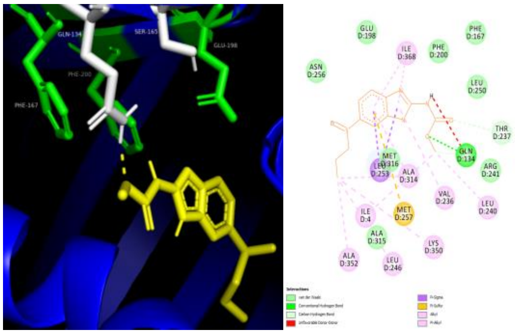

ASA-ABZSO -5.7 kcal/mol

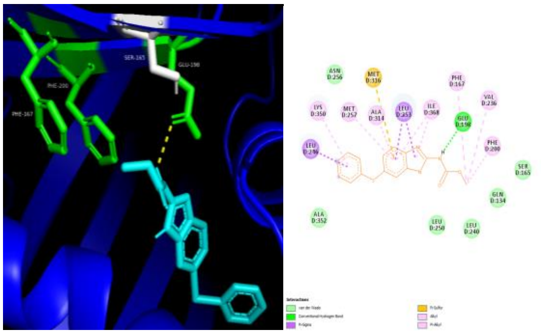

ASA-FBZ -6 kcal/mol

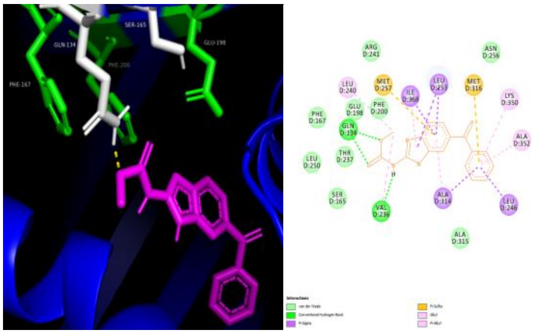

ASA-MBZ -6.2 kcal/mol

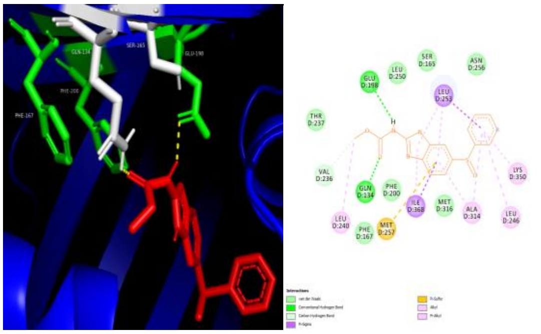

ASA-OXBZ -6.2 kcal/mol

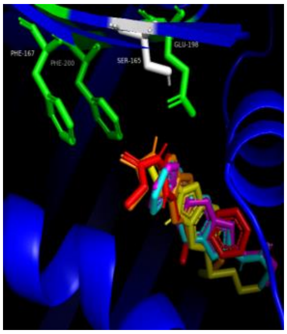

ASA-all drugs

Supplementary Figure S22: Autodock vina docking results for *A. suum* isotype A and several benzimidazole drugs. The drugs used are albendazole (ABZ), albendazole sulfoxide (ABZSO), fenbendazole (FBZ), mebendazole (MBZ) and oxfendazole (OXBZ). 3D and 2D models are shown for each docking result. Binding affinities are shown underneath each model. In ABZ docking H-bonds form with Q134 and E198 and an additional unfavourable acceptor-acceptor bond with E198 is also seen in the 2D models. For ABZSO the only H-bond seen is with Q134 in both models, and again an unfavourable bond is seen in the 2D model, this time a donor-donor bond is made with Q134. In both FBZ models the only bond found is with E198. For MBZ the 3D model shows a single H-bond with Q134 whereas in the 2D model two bonds are made with Q134 and one is made with V236. Finally, in both OXBZ models one H-bond is formed with Q134 and E198

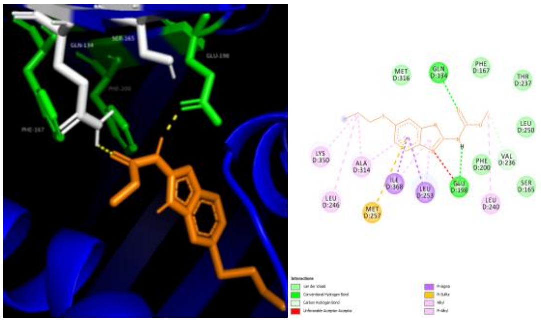

ALA- ABZ -5.7 kcal/mol

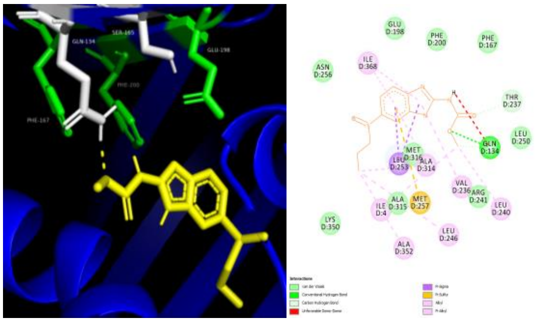

ALA- ABZSO -5.7 kcal/mol

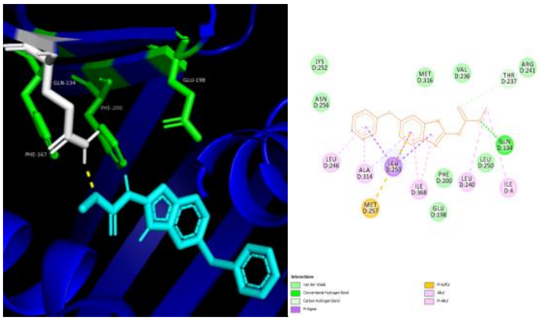

ALA- FBZ -6.1 kcal/mol

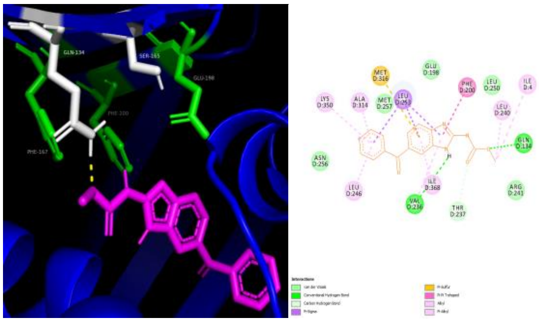

ALA- MBZ -5.8 kcal/mol

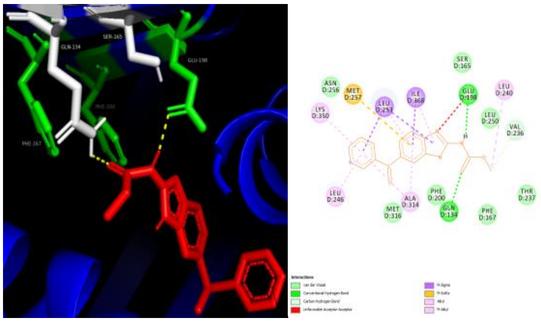

ALA- OXBZ -6.3 kcal/mol

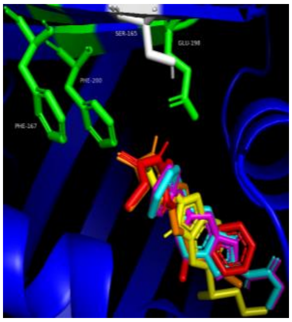

ALA- all drugs

Supplementary Figure S23: Autodock vina docking results for *A. lumbricoides* isotype A and several benzimidazole drugs. The drugs used are albendazole (ABZ), albendazole sulfoxide (ABZSO), fenbendazole (FBZ), mebendazole (MBZ) and oxfendazole (OXBZ). 3D and 2D models are shown for each docking result. Binding affinities are shown underneath each model. In ABZ docking H-bonds form with Q134 and E198 and an additional unfavourable acceptor-acceptor bond with E198 is also seen in the 2D models. For ABZSO the only H-bond seen is with Q134 in both models, and again an unfavourable bond is seen in the 2D model, this time a donor-donor bond is made with Q134. In the FBZ binding models single H-bonds are predicted with L253 and N256. For MBZ the 3D model shows a single H-bond with Q134 whereas the 2D model shows an extra bond is made with V236. Both models for OXBZ show one H-bond with Q134 and E198, yet in the 2D model and additional unfavourable acceptor-acceptor bond with E198.

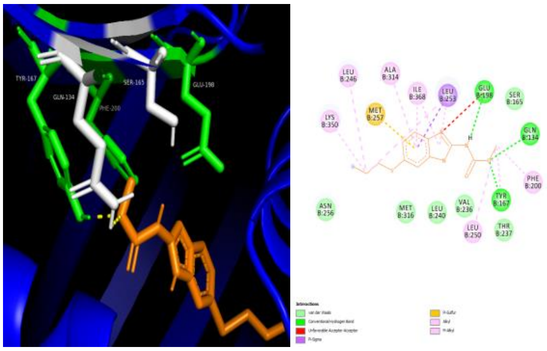

ASA_167Y-ABZ -4.4 kcal/mol

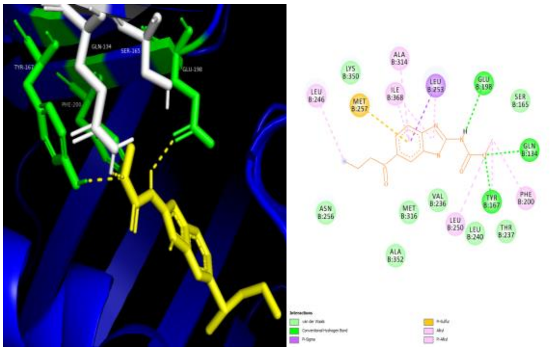

ASA_167Y-ABZSO -4.6 [illegible]

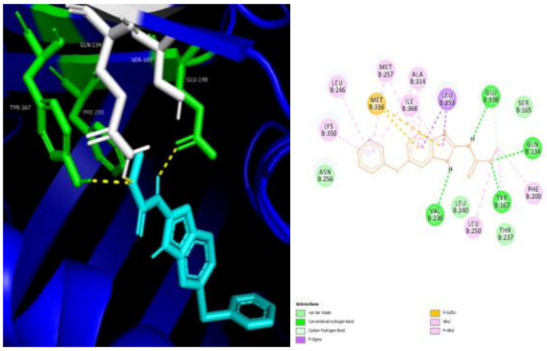

ASA_167Y-FBZ -5 kcal/mol

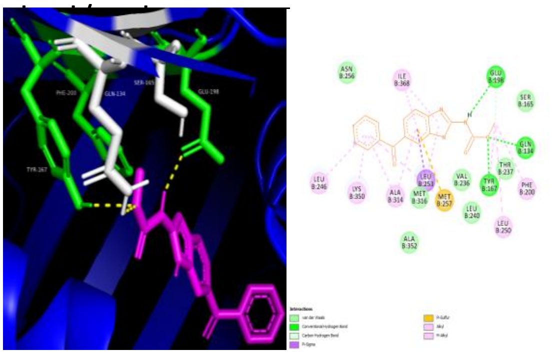

ASA_167Y-MBZ -6 kcal/mol

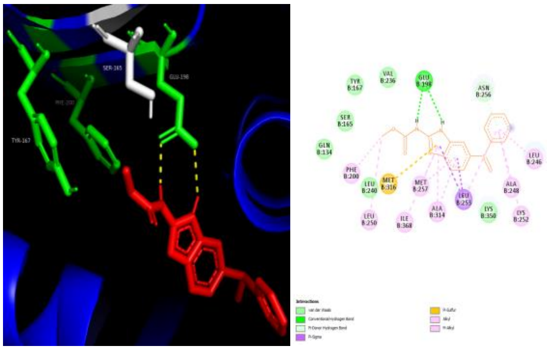

ASA_167Y-OXBZ -4.1 kcal/mol

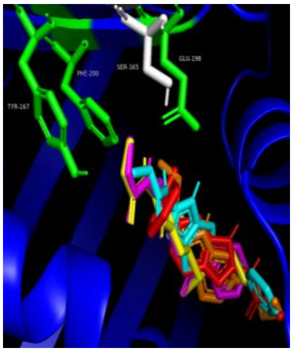

ASA_167Y-all drugs

Supplementary Figure S24: Autodock vina docking results for *A. suum* 167Y mutated isotype A and several benzimidazole drugs. The drugs used are albendazole (ABZ), albendazole sulfoxide (ABZSO), fenbendazole (FBZ), mebendazole (MBZ) and oxfendazole (OXBZ). 3D and 2D models are shown for each docking result. Binding affinities are shown underneath each model. In these mutated 167Y models we see the larger tyrosine amino acid forming bonds with the drugs whereas the natural phenylalanine amino acid does not interact in any other amino acids. In ABZ 3D model we find H-bond formation with Q134 and 167Y. In the 2D model, however, we also see an additional H-bond and unfavourable acceptor-acceptor bond with E198. For ABZSO and MBZ both 2D and 3D models show one H-bond with Q134, 167Y and E198. For FBZ we again see H-bond formation with Q134, 167Y and E198, but an additional bond with V236 is also seen in the 2D model. In both models for OXBZ two H-bonds are made with E198 only.

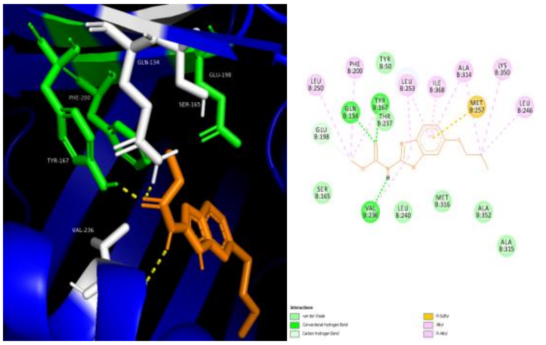

ALA_167Y- ABZ -4.4 kcal/mol

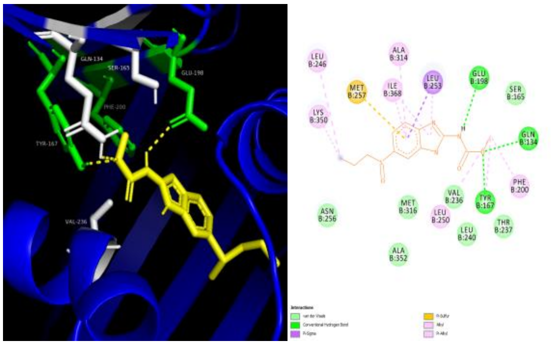

ALA_167Y- ABZSO -4.6 [illegible]

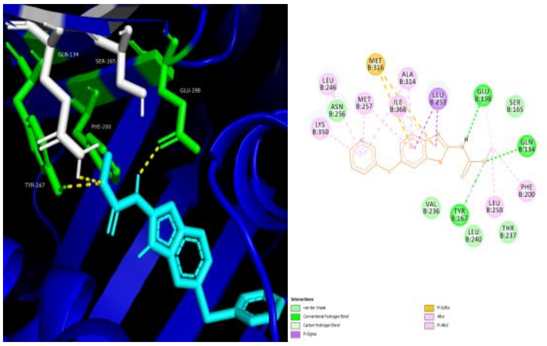

ALA_167Y- FBZ -5 kcal/mol

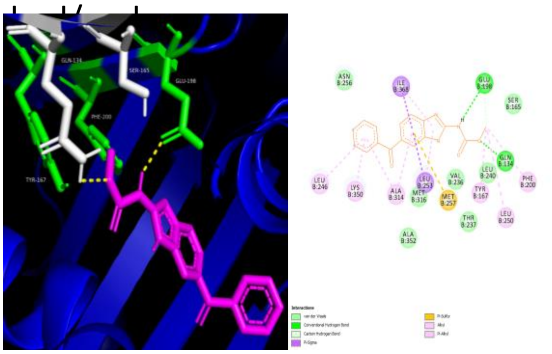

ALA_167Y- MBZ -5.9 [illegible]

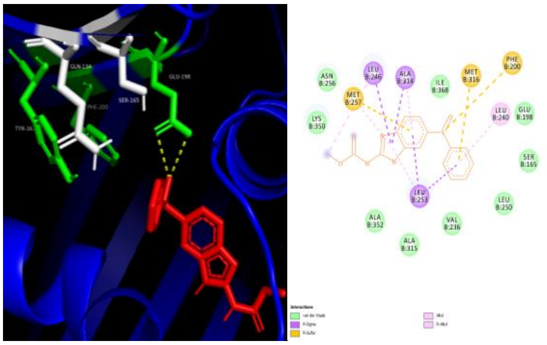

ALA_167Y- OXBZ -3.3 kcal/mol

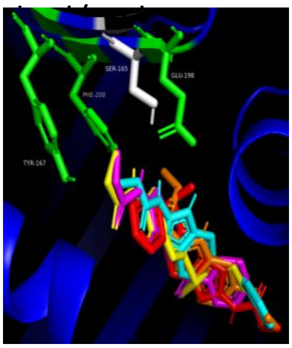

ALA_167Y- all drugs

Supplementary Figure S25: Autodock vina docking results for *A. lumbricoides* 167Y mutated isotype A and several benzimidazole drugs. The drugs used are albendazole (ABZ), albendazole sulfoxide (ABZSO), fenbendazole (FBZ), mebendazole (MBZ) and oxfendazole (OXBZ). 3D and 2D models are shown for each docking result. Binding affinities are shown underneath each model. In these mutated 167Y models we see the larger tyrosine amino acid forming bonds with the drugs whereas the natural phenylalanine amino acid does not interact in any other amino acids. In ABZ H-bonds are formed with Q134, 167Y and V236 in both models. For both ABZSO and FBZ H-bonds are made with Q134, 167Y and E198 in 2D and 3D models. In MBZ both models show H-bond formation with Q134 and E198. Finally, in OXBZ two H-bonds are made with E198 in the 3D model but none are predicted in the 2D model.

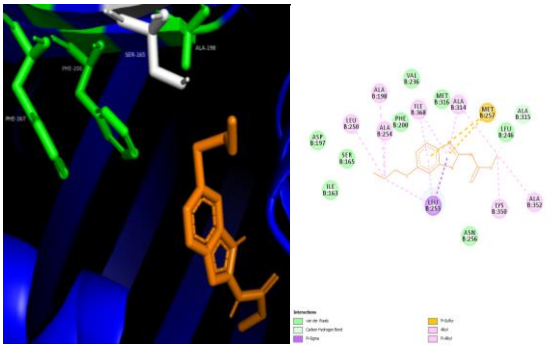

ASA_198A-ABZ -5.7 kcal/mol

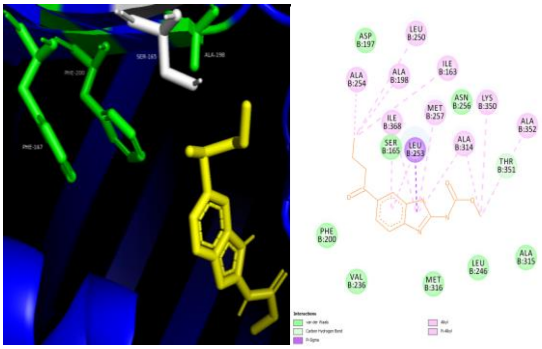

ASA_198A-ABZSO -6.2kcal/mol

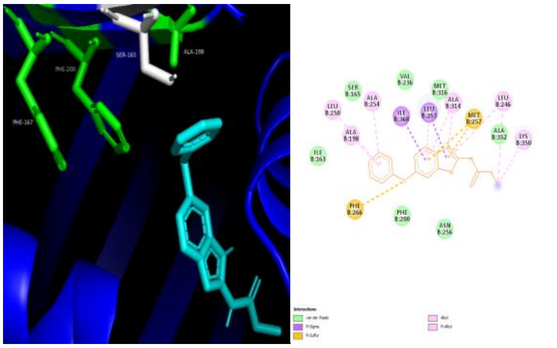

ASA_198A-FBZ -6.2 kcal/mol

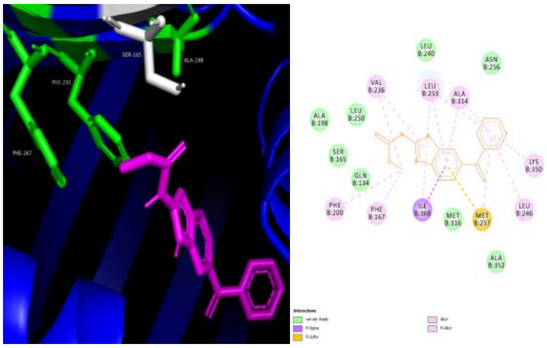

ASA_198A-MBZ -7.6 kcal/mol

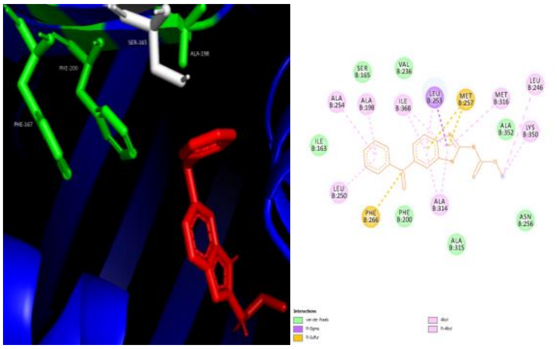

ASA_198A-OXBZ -6.1 kcal/mol

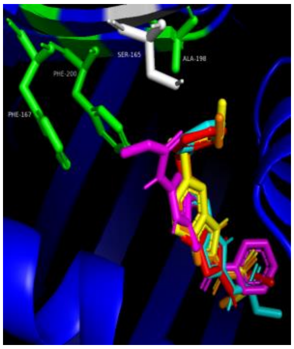

ASA_198A-all drugs

Supplementary Figure S26: Autodock vina docking results for *A. suum* 198A mutated isotype A and several benzimidazole drugs. The drugs used are albendazole (ABZ), albendazole sulfoxide (ABZSO), fenbendazole (FBZ), mebendazole (MBZ) and oxfendazole (OXBZ). 3D and 2D models are shown for each docking result. Binding affinities are shown underneath each model. When the mutated 198A amino acid is present, it affects the binding and no H-bond formation is seen with any drug.

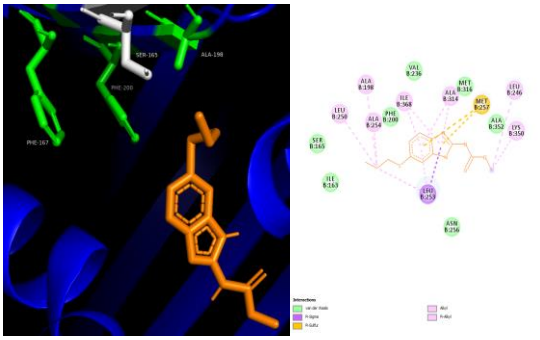

ALA_198A- ABZ -5.7 kcal/mol

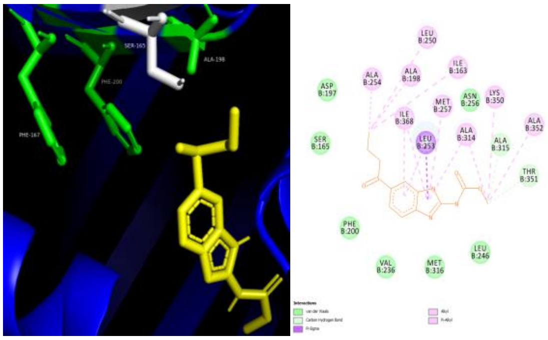

ALA_198A- ABZSO -6.2 kcal/mol

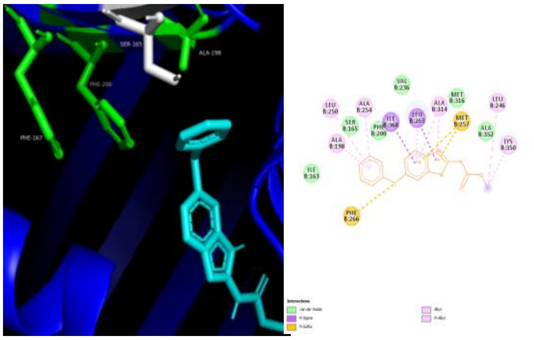

ALA_198A- FBZ -6.2 kcal/mol

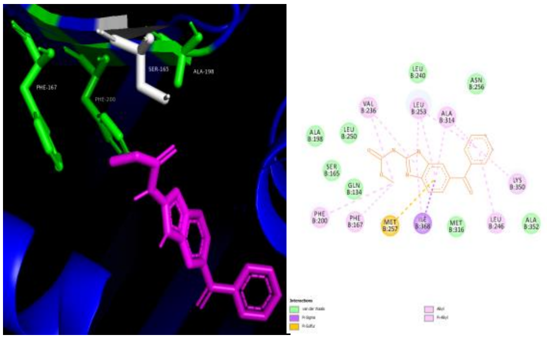

ALA_198A- MBZ -7.6 kcal/mol

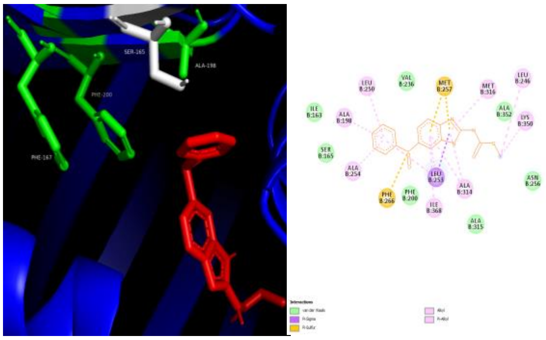

ALA_198A- OXBZ -6.1 kcal/mol

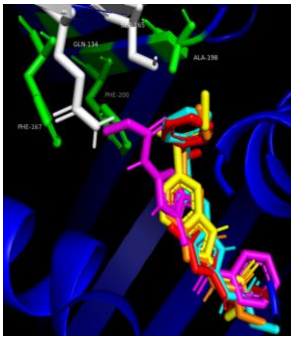

ALA_198A- all drugs

Supplementary Figure S27: Autodock vina docking results for *A. lumbricoides* 198A mutated isotype A and several benzimidazole drugs. The drugs used are albendazole (ABZ), albendazole sulfoxide (ABZSO), fenbendazole (FBZ), mebendazole (MBZ) and oxfendazole (OXBZ). 3D and 2D models are shown for each docking result. Binding affinities are shown underneath each model. When the mutated 198A amino acid is present, it affects the binding and no H-bond formation is seen with any drug.

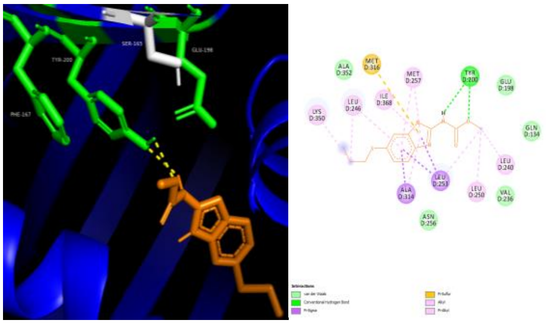

ASA_200Y-ABZ -5.5 kcal/mol

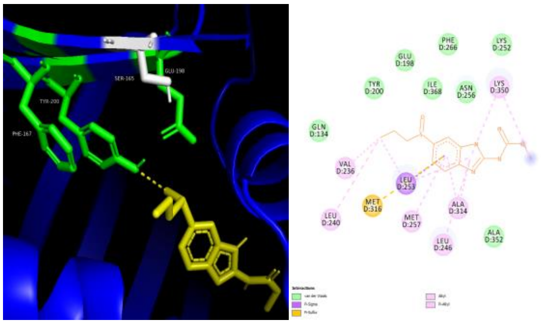

ASA_200Y-ABZSO -5.2 [illegible]

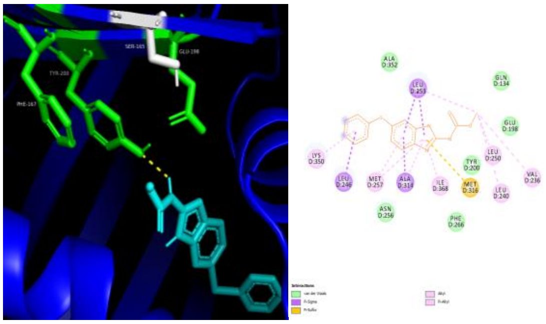

ASA_200Y-FBZ -6 kcal/mol

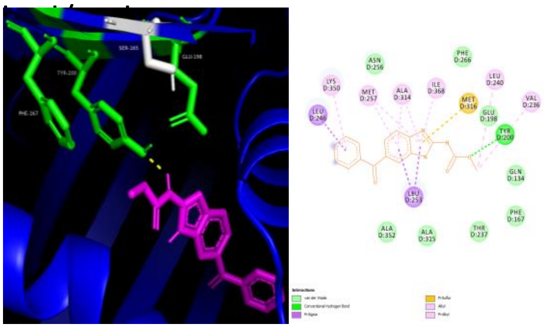

ASA_200Y-MBZ -6.4 kcal/mol

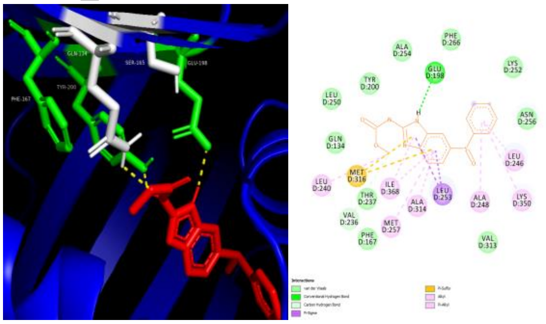

ASA_200Y-OXBZ -4.4 kcal/mol

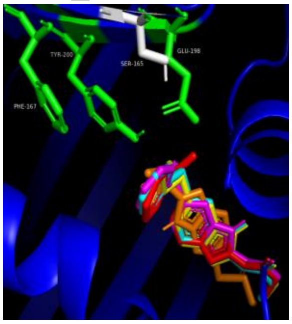

ASA_200Y-all drugs

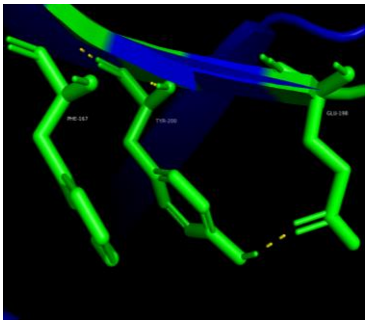

ASA_200Y- Self binding

Supplementary Figure S28: Autodock vina docking results for *A. suum* 200Y mutated isotype A and several benzimidazole drugs. The drugs used are albendazole (ABZ), albendazole sulfoxide (ABZSO), fenbendazole (FBZ), mebendazole (MBZ) and oxfendazole (OXBZ). 3D and 2D models are shown for each docking result. Binding affinities are shown underneath each model. When amino acid 200 is mutated to tyrosine the close proximity between the 200Y and E198 amino acids allows H-bonds to form between them. In ABZ both models show two H-bonds with 200Y. For ABZSO and FBZ one H-bond is seen with 200Y in the 3D models but no bonds are seen in the 2D models. In MBZ a H-bond is formed with 200Y in both models. The 3D models for OXBZ show H-bonds with Q134, E198 and 200Y but only the E198 bond is seen in the 2D model.

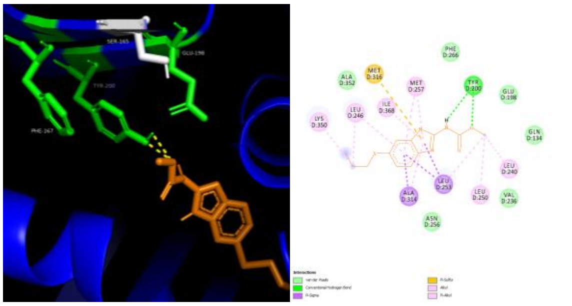

ALA_200Y- ABZ -5.4 kcal/mol

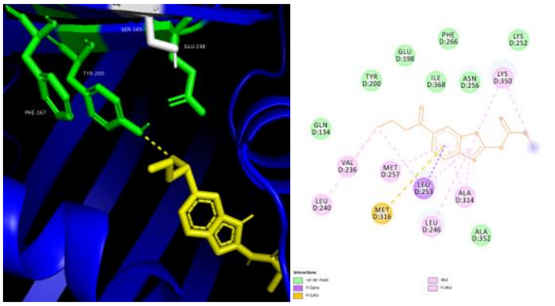

ALA_200Y- ABZSO -5.3 kcal/mol

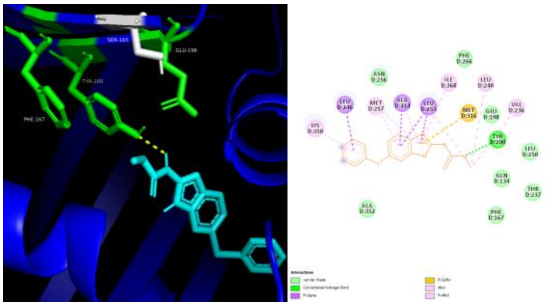

ALA_200Y- FBZ -6 kcal/mol

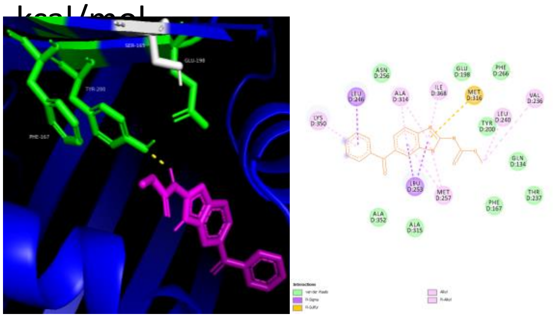

ALA_200Y- MBZ -6.6 kcal/mol

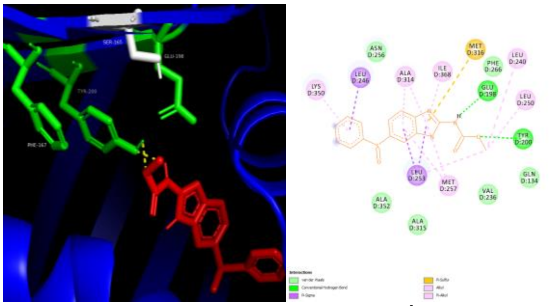

ALA_200Y- OXBZ -4.7 kcal/mol

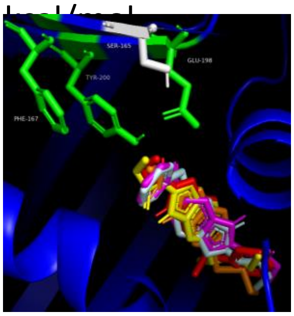

ALA_200Y- all drugs

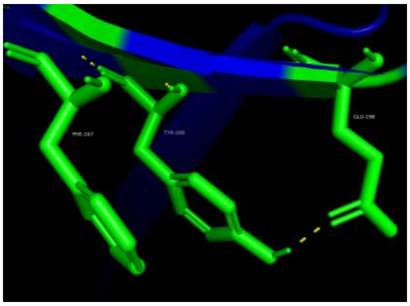

ALA_200Y- self binding

Supplementary Figure S29: Autodock vina docking results for *A. lumbricoides* 200Y mutated isotype A and several benzimidazole drugs. The drugs used are albendazole (ABZ), albendazole sulfoxide (ABZSO), fenbendazole (FBZ), mebendazole (MBZ) and oxfendazole (OXBZ). 3D and 2D models are shown for each docking result. Binding affinities are shown underneath each model. When amino acid 200 is mutated to tyrosine the close proximity between the 200Y and E198 amino acids allows H-bonds to form between them. In ABZ both models show two H-bonds with 200Y. For ABZSO and MBZ one H-bond is seen with 200Y in the 3D models but no bonds are seen in the 2D models. In FBZ a H-bond is formed with 200Y in both models. The OXBZ 3D model shows two H-bonds with 200Y but the 2D model shows only one with 200Y and E198.

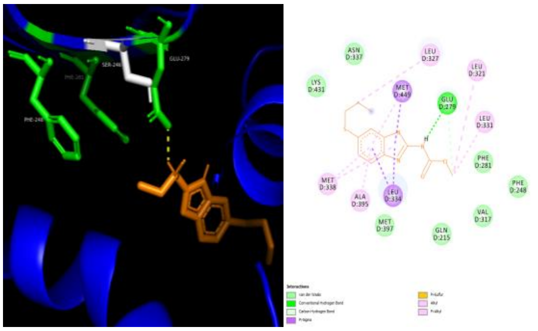

ASB-ABZ -4.3 kcal/mol

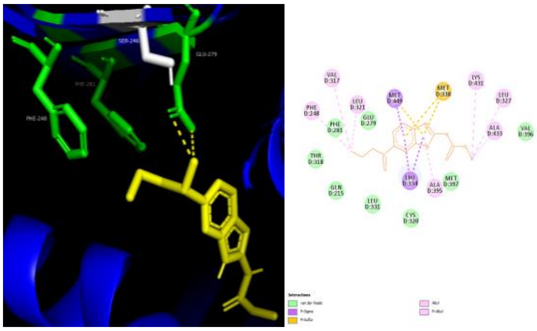

ASB-ABZSO -4.6 kcal/mol

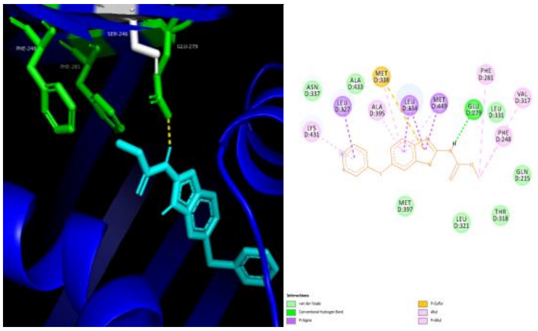

ASB-FBZ -5.1 kcal/mol

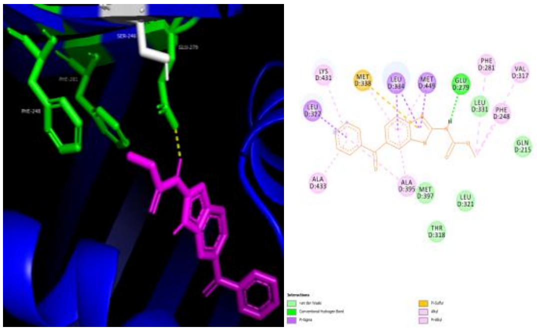

ASB-MBZ -6.5 kcal/mol

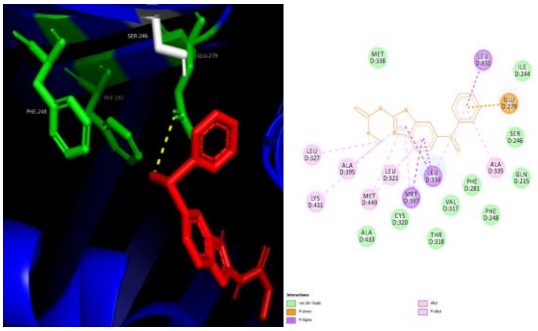

ASB-OXBZ -3.3 kcal/mol

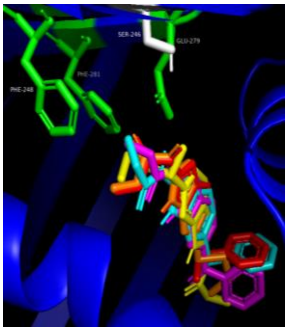

ASB-all drugs

Supplementary Figure S30: Autodock vina docking results for *A. suum* isotype B and several benzimidazole drugs. The drugs used are albendazole (ABZ), albendazole sulfoxide (ABZSO), fenbendazole (FBZ), mebendazole (MBZ) and oxfendazole (OXBZ). 3D and 2D models are shown for each docking result. Binding affinities are shown underneath each model. A single H-bond with E198 (labelled as E279 due to isotype B being a longer protein) is seen in all models for each drug, the exception is that for the ABZSO and OXBZ 2D models no H-bonds are found.

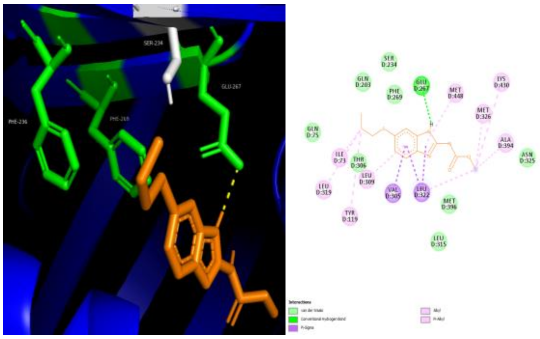

ALB- ABZ -5.9 kcal/mol

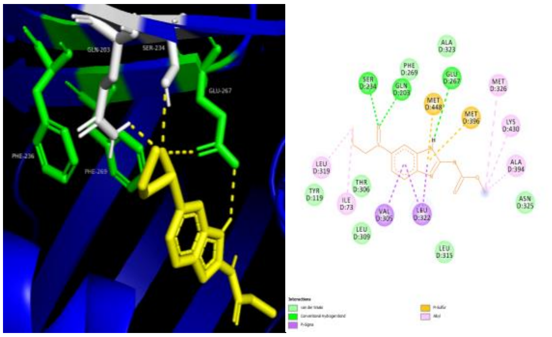

ALB- ABZSO -6.1 kcal/mol

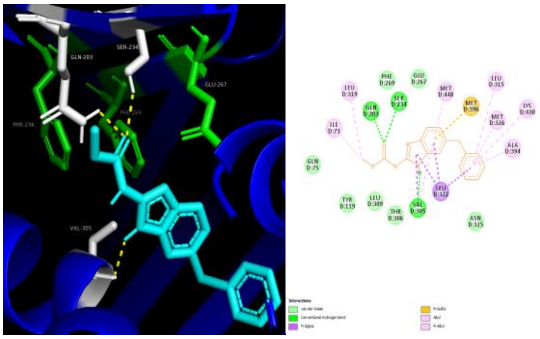

ALB- FBZ -7.3 kcal/mol

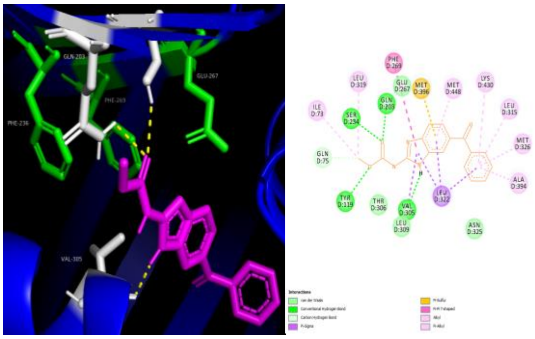

ALB- MBZ -8.5 kcal/mol

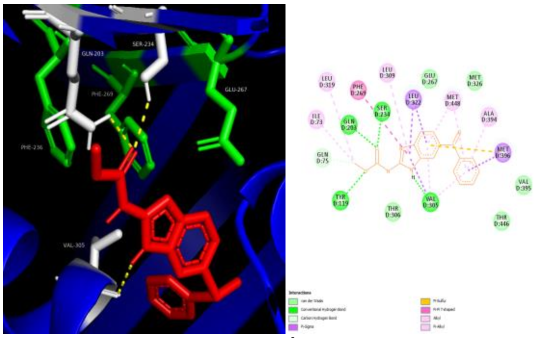

ALB- OXBZ -6 kcal/mol

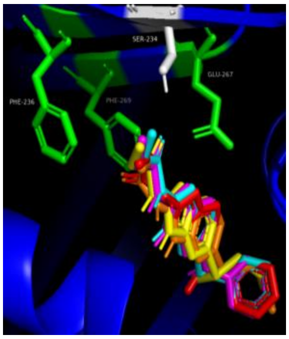

ALB- all drugs

Supplementary Figure S31: Autodock vina docking results for *A. lumbricoides* isotype B and several benzimidazole drugs. The drugs used are albendazole (ABZ), albendazole sulfoxide (ABZSO), fenbendazole (FBZ), mebendazole (MBZ) and oxfendazole (OXBZ). 3D and 2D models are shown for each docking result. Binding affinities are shown underneath each model. For ABZ a single H-bond with E198 (labelled as E267 due to isotype B being a longer protein) is seen in both 2D and 3D models. For ABZSO we find two H-bonds with E198, one with Q134 (Q203) and one with S165 (S234) in the 3D model, with only one bond seen to each in the 2D model. For FBZ, MBZ and OXBZ the models show H-bonds to Q134, S165 and V236 (V305) in all models, with the addition of Y50 (Y119) in the 2D models for MBZ and OXBZ.

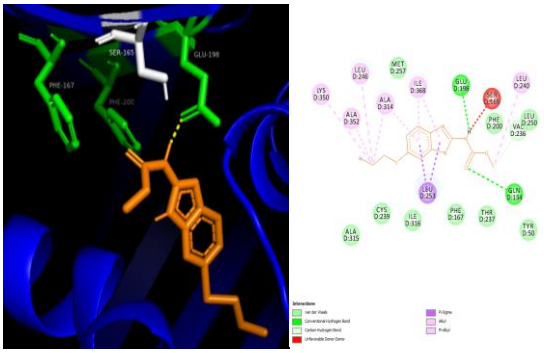

ASC-ABZ -6.6 kcal/mol

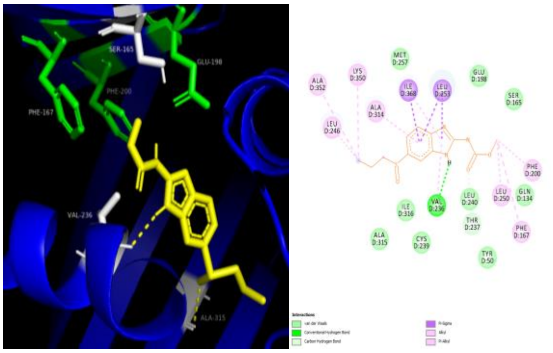

ASC-ABZSO -6.6 kcal/mol

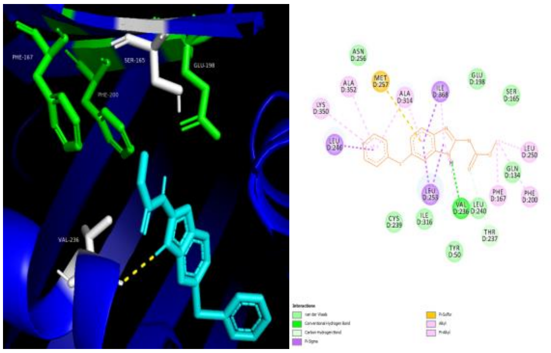

ASC-FBZ -7.8 kcal/mol

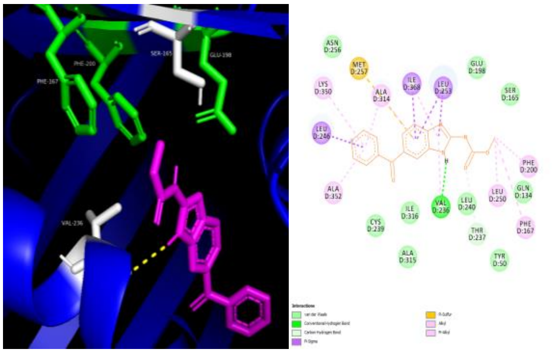

ASC-MBZ -8.4 kcal/mol

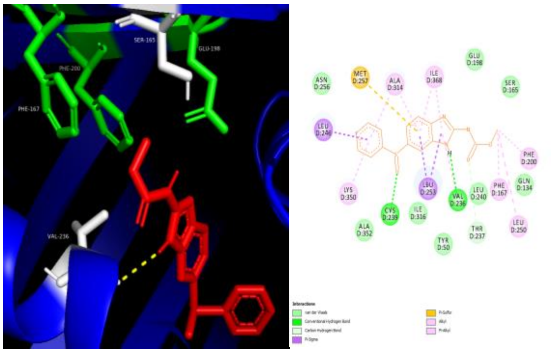

ASC-OXBZ -7.2 kcal/mol

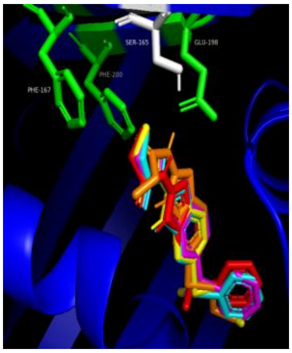

ASC-all drugs

Supplementary Figure S32: Autodock vina docking results for *A. suum* isotype C and several benzimidazole drugs. The drugs used are albendazole (ABZ), albendazole sulfoxide (ABZSO), fenbendazole (FBZ), mebendazole (MBZ) and oxfendazole (OXBZ). 3D and 2D models are shown for each docking result. Binding affinities are shown underneath each model. In ABZ 3D docking models we find a H-bond formed with E198, yet in the 2D models we see that on top of this there is also a bond with Q134 and although unfavourable there is a donor-donor bond with S165. For all other drugs we see a single H-bond formed with V236 in both models, with an extra bond to A315 in ABZSO 3D model and C239 in the OXBZ 2D model.

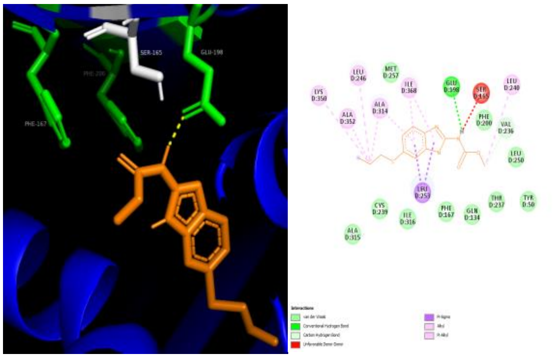

ALC- ABZ -6.6 kcal/mol

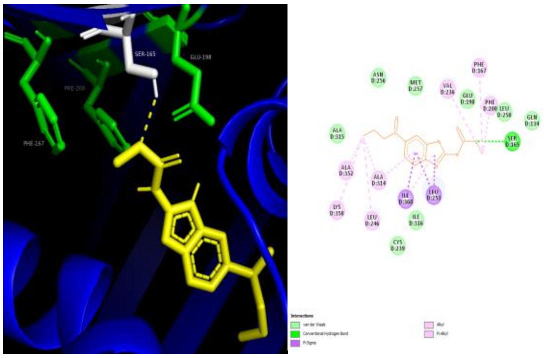

ALC- ABZSO -6.1 kcal/mol

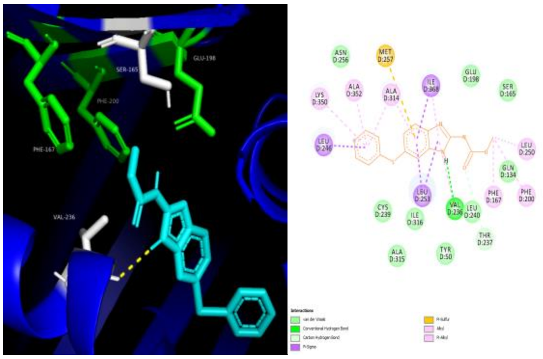

ALC- FBZ -7.8 kcal/mol

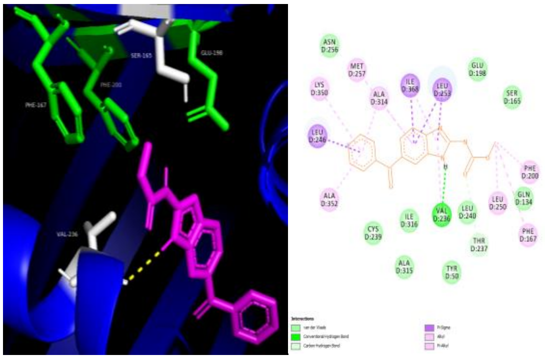

ALC- MBZ -8.5 kcal/mol

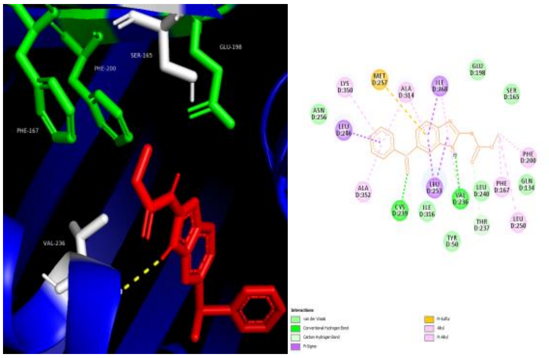

ALC- OXBZ -7.2 kcal/mol

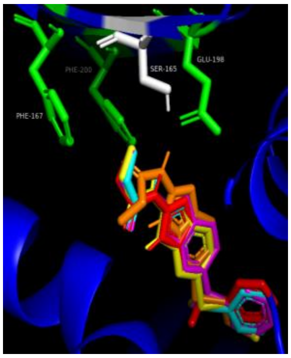

ALC- all drugs

Supplementary Figure S33: Autodock vina docking results for *A. lumbricoides* isotype C and several benzimidazole drugs. The drugs used are albendazole (ABZ), albendazole sulfoxide (ABZSO), fenbendazole (FBZ), mebendazole (MBZ) and oxfendazole (OXBZ). 3D and 2D models are shown for each docking result. Binding affinities are shown underneath each model. In ABZ 3D docking models we find a H-bond formed with E198, yet in the 2D models we see that on top of this there is also a bond with Q134 and although unfavourable there is a donor-donor bond with S165. In the ABZSO 3D and 2D docking models one H-bond is formed with S165 only. For all other drugs we see a single H-bond formed with V236 in both models, with an extra bond to A315 in ABZSO 3D model and C239 in the OXBZ 2D model.

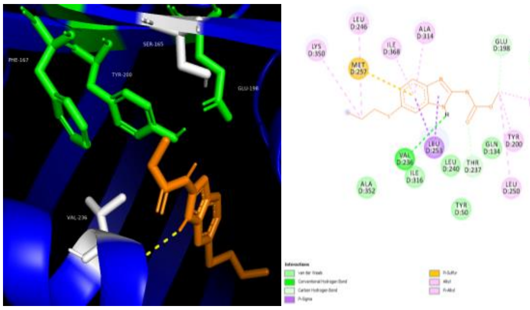

ASD-ABZ -6.2 kcal/mol

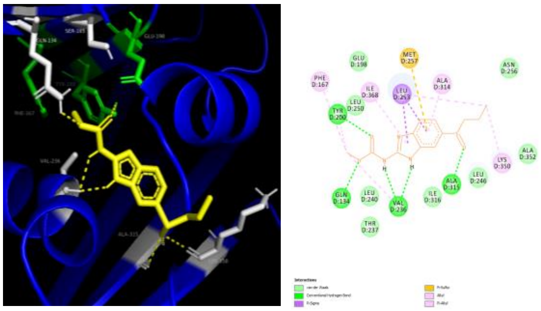

ASD-ABZSO -6.8 kcal/mol

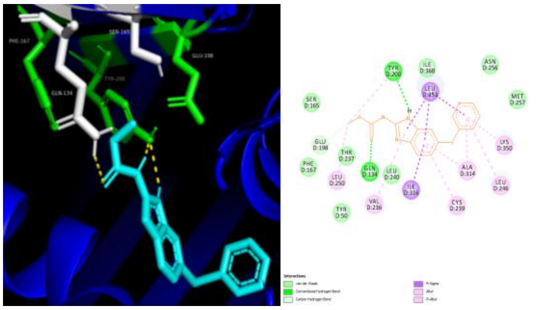

ASD-FBZ -7.1 kcal/mol

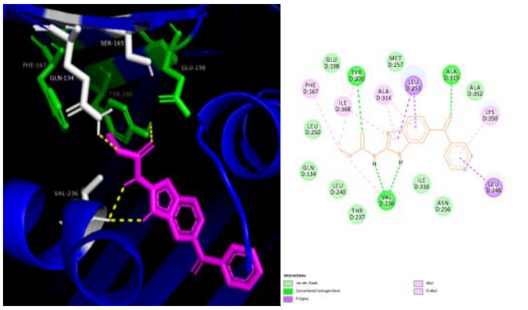

ASD-MBZ -8.6 kcal/mol

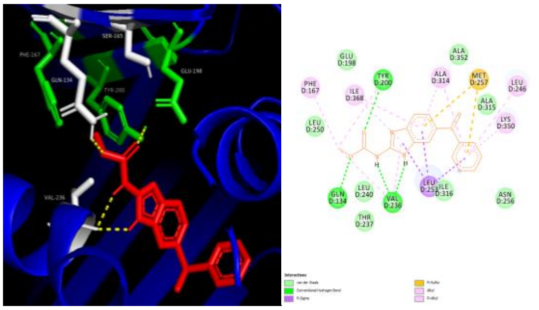

ASD-OXBZ -6.3 kcal/mol

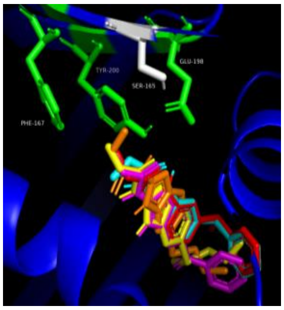

ASD-all drugs

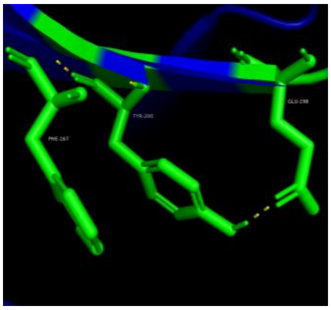

ASD-self binding

Supplementary Figure S34: Autodock vina docking results for *A. suum* isotype D and several benzimidazole drugs. The drugs used are albendazole (ABZ), albendazole sulfoxide (ABZSO), fenbendazole (FBZ), mebendazole (MBZ) and oxfendazole (OXBZ). 3D and 2D models are shown for each docking result. Binding affinities are shown underneath each model. A single H-bond is made with V236 and ABZ in both 3D and 2D models. For ABZSO we see two H-bonds with amino acids V236 and A315 and one H-bond with Q134, Y200 and K350 in the 3D model. In the 2D model we again find two H-bonds with V236, but only a single bond with A315, along with Q134 and Y200. In the FBZ models we see one H-bond made with Q134 in both 3D and 2D model, H-bonds are also formed with Y200 in both models with two seen in the 3D and one in the 2D models. For MBZ we see the formation of two H-bonds to V236 and one with Y200 in both models, in addition to this the 3D model shows an extra bond with Q134 and the 2D model shows a bond with A315. Finally, in the OXBZ models we again find the formation of two H-bonds with V236, along with single bonds to Q134 and Y200 in both models.

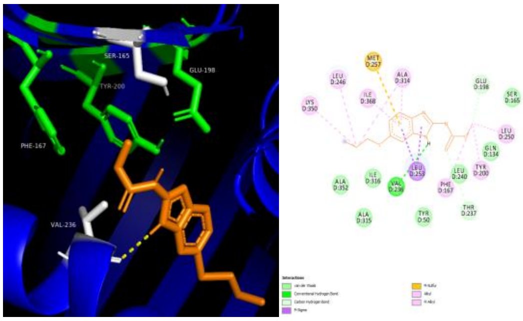

ALD- ABZ -6.2 kcal/mol

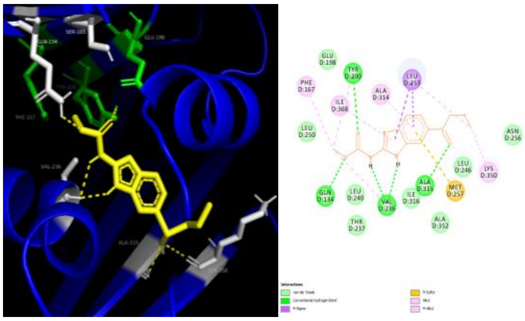

ALD- ABZSO -6.8 kcal/mol

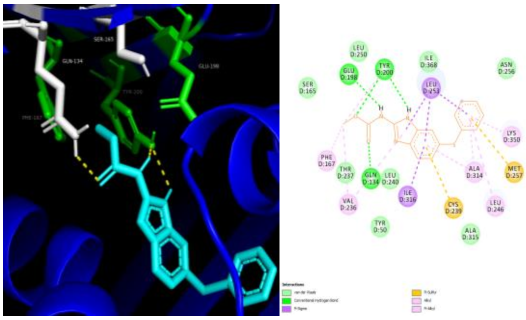

ALD- FBZ -7 kcal/mol

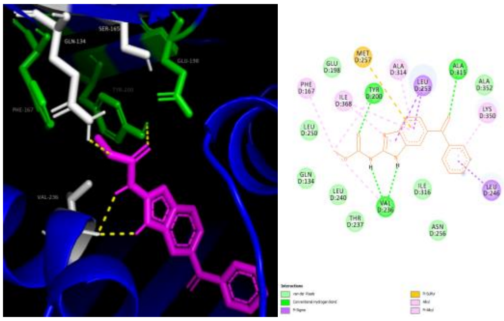

ALD- MBZ -8.5 kcal/mol

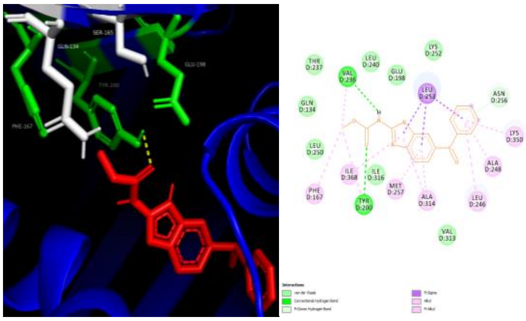

ALD- OXBZ -7.1 kcal/mol

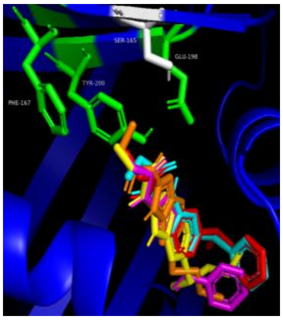

ALD- all drugs

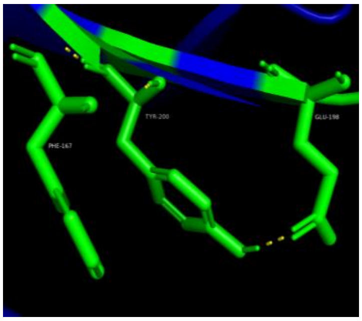

ALD- self binding

Supplementary Figure S35: Autodock vina docking results for *A. lumbricoides* isotype D and several benzimidazole drugs. The drugs used are albendazole (ABZ), albendazole sulfoxide (ABZSO), fenbendazole (FBZ), mebendazole (MBZ) and oxfendazole (OXBZ). 3D and 2D models are shown for each docking result. Binding affinities are shown underneath each model. For ABZ models one H-bond is formed with Q134 in both models. The 3D model then shows a H-bond with E198, whilst the 2D models show two bonds between the drug and E198. For ABZSO we see two H-bonds with amino acids V236 and A315 and one H-bond with Q134, Y200 and K350 in the 3D model. In the 2D model we again find two H-bonds with V236, but only a single bond with A315, along with Q134 and Y200. With FBZ two H-bonds form with Y200 and one forms with Q134 in both models, there is also an additional bond with E198 seen in the 2D model. For MBZ we see the formation of two H-bonds to V236 and one with Y200 in both models, in addition to this the 3D model shows an extra bond with Q134 and the 2D model shows a bond with A315. The OXBZ models both show a single H-bond formation with Y200, however the 2D models show an additional bond with V236.

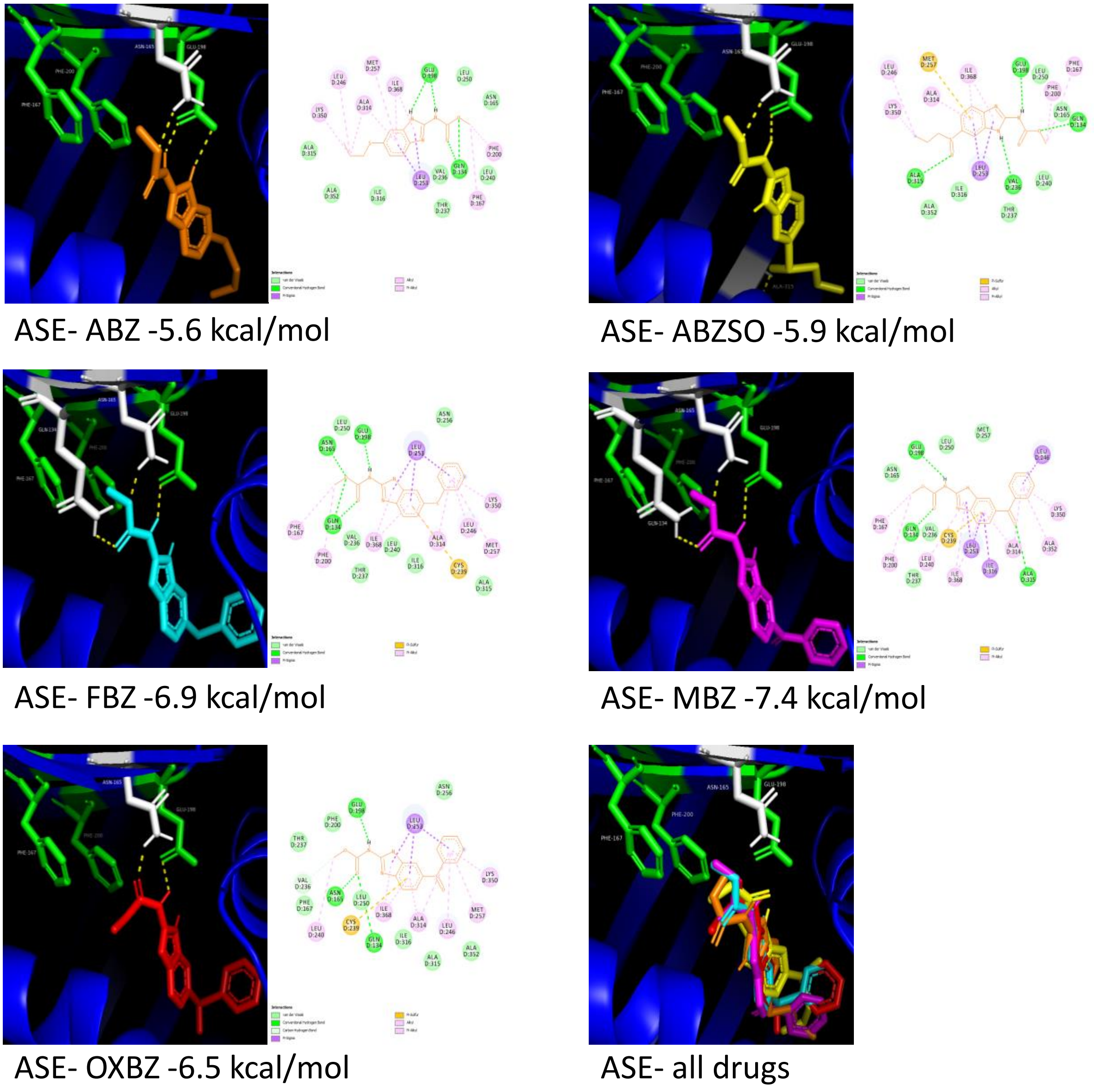

Supplementary Figure S36: Autodock vina docking results for *A. suum* isotype E and several benzimidazole drugs. The drugs used are albendazole (ABZ), albendazole sulfoxide (ABZSO), fenbendazole (FBZ), mebendazole (MBZ) and oxfendazole (OXBZ). 3D and 2D models are shown for each docking result. Binding affinities are shown underneath each model. In ABZ, docking shows two H-bonds with E198 and one with N165 in the 3D model but two H-bonds with both Q134 and E198 in the 2D model. With ABZSO the 3D models show one H-bond with N165 and E198, however the 2D model predicts H-bonds with Q134, E198, V236 and A315. Both models for FBZ find single H-bonds with Q134, N165 and E198. In the MBZ 3D model one H-bond is predicted to form with Q134, N165 and E198 whereas in the 2D model the H-bonds are seen with Q134, E198 and A315. Finally, the OXBZ 3D model predicts H-bonds with N165 and E198, whilst the 2D model shows an additional bond with Q134.

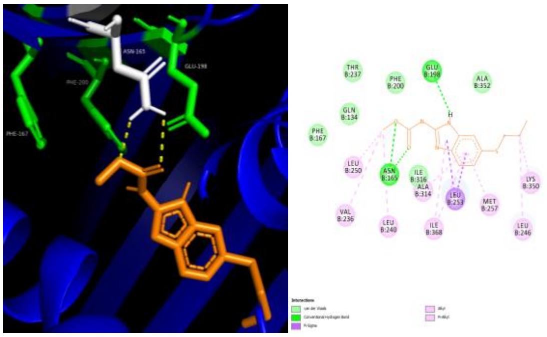

ALE- ABZ -6.1 kcal/mol

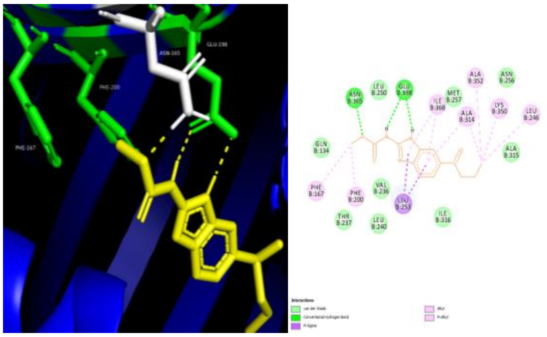

ALE- ABZSO -6.8 kcal/mol

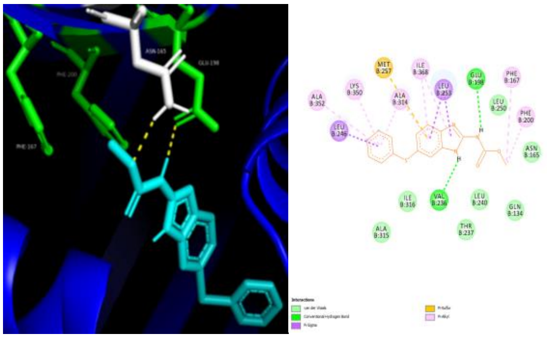

ALE- FBZ -7.5 kcal/mol

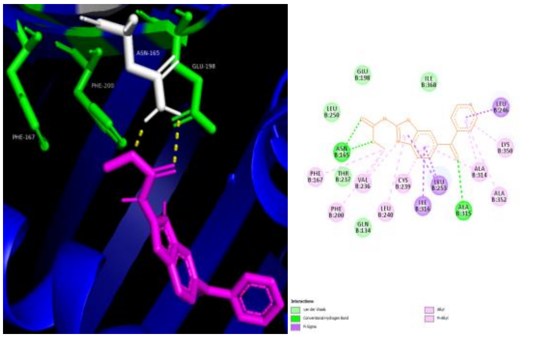

ALE- MBZ -8.3 kcal/mol

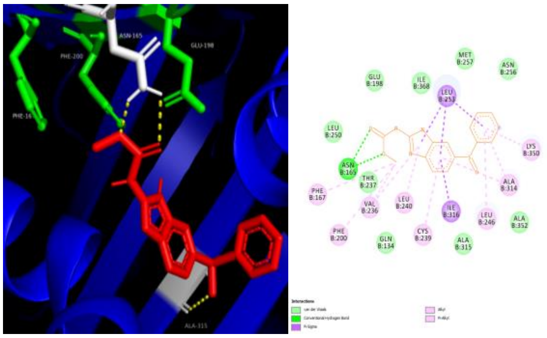

ALE- OXBZ -7.3 kcal/mol

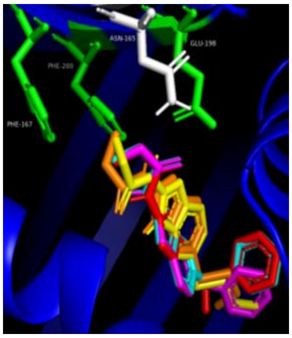

ALE- all drugs

Supplementary Figure S37: Autodock vina docking results for *A. lumbricoides* isotype E and several benzimidazole drugs. The drugs used are albendazole (ABZ), albendazole sulfoxide (ABZSO), fenbendazole (FBZ), mebendazole (MBZ) and oxfendazole (OXBZ). 3D and 2D models are shown for each docking result. Binding affinities are shown underneath each model. In ABZ docking two H-bonds are seen with N165 in the 3D model with an additional bond with E198 shown in the 2D model. for ABZSO both models predict the formation of one H-bond to N165 and two H-bonds to E198. For FBZ H-bond formation with N165 and E198 is seen in the 3D model, whereas H-bonds are made with E198 and V236 in the 2D model. In the 3D model for MBZ two H-bonds are made with N165 and in the 2D model an additional bond with A315 is seen. Finally, for OXBZ the 3D model shows two H-bonds with N165 and one with A315, however the 2D model only finds the two bonds with N165.

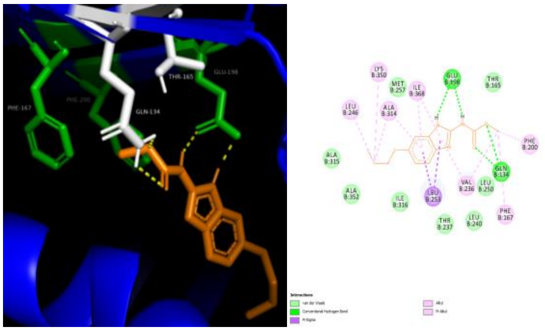

ASF- ABZ -7.2 kcal/mol

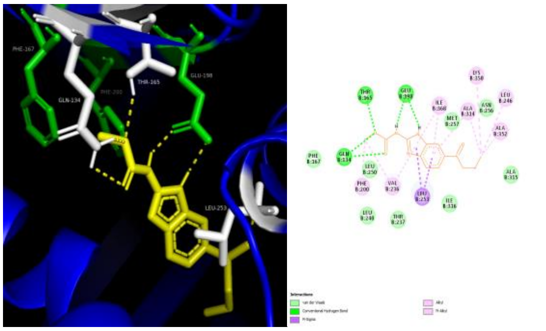

ASF- ABZSO -7.7 kcal/mol

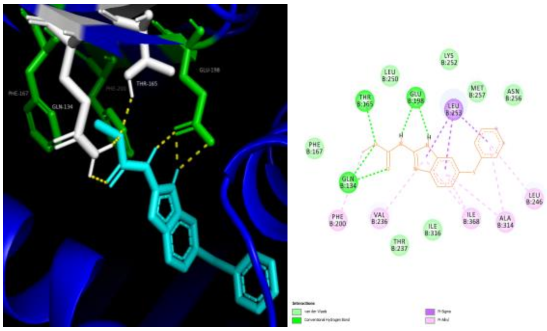

ASF- FBZ -7.5 kcal/mol

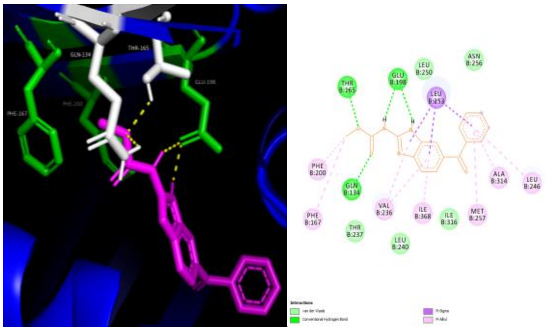

ASF- MBZ -8.4 kcal/mol

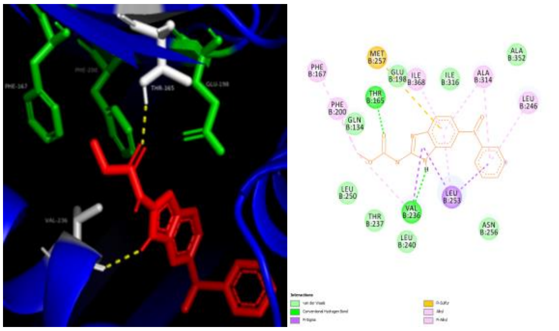

ASF- OXBZ -7.1 kcal/mol

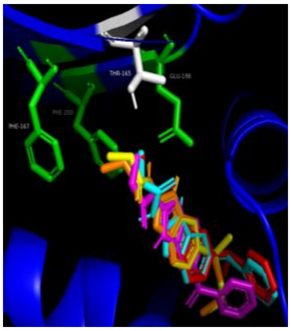

ASF- all drugs

Supplementary Figure S38: Autodock vina docking results for *A. suum* isotype F and several benzimidazole drugs. The drugs used are albendazole (ABZ), albendazole sulfoxide (ABZSO), fenbendazole (FBZ), mebendazole (MBZ) and oxfendazole (OXBZ). 3D and 2D models are shown for each docking result. Binding affinities are shown underneath each model. In ABZ both docking models predict two H-bonds with both Q134 and E198. For ABZSO two H-bonds are made with Q134 and E198 and a single bond is formed with T165 in both 3D and 2D models. The FBZ 3D model predicts three H-bonds with E198, two H-bonds with Q 134 and one bond with T165, the 2D model is similar but only shows two H-bonds with E198. In both models for MBZ two H-bonds are seen with E198 and one is seen with Q134 and T165. The final drug, OXBZ has H-bonds with T165 and V236 in both models.

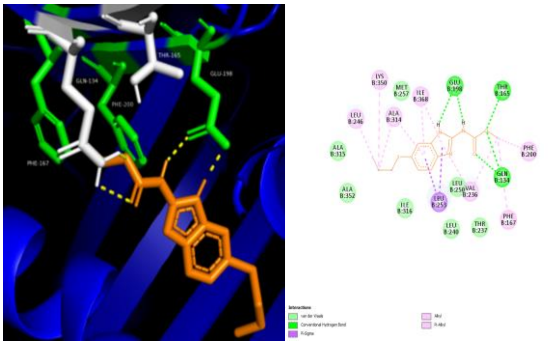

ALF- ABZ -7.2 kcal/mol

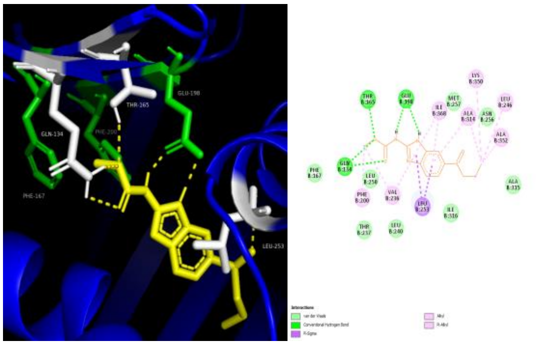

ALF- ABZSO -7.8 kcal/mol

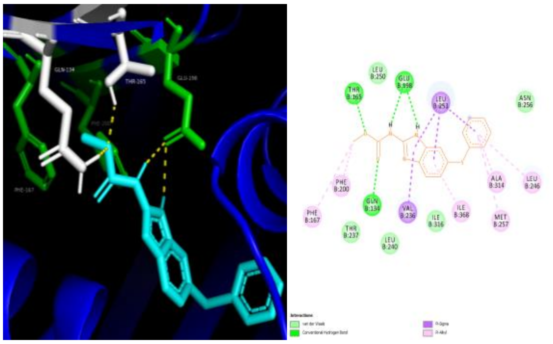

ALF- FBZ -7.4 kcal/mol

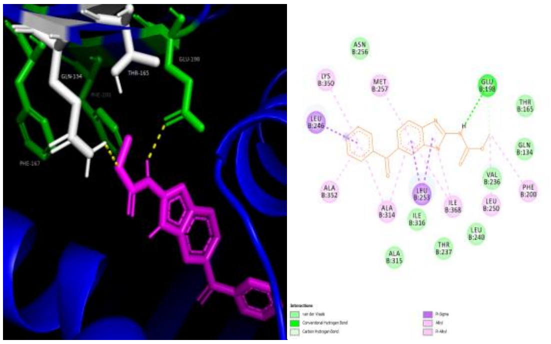

ALF- MBZ -8.7 kcal/mol

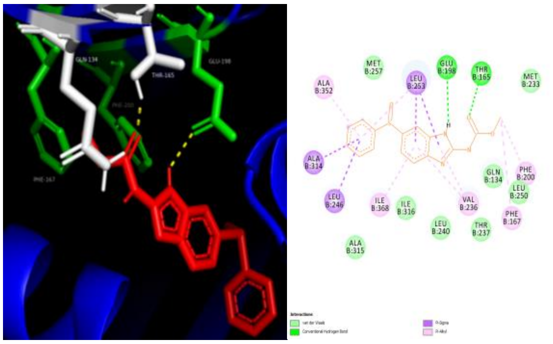

ALF- OXBZ -6 kcal/mol

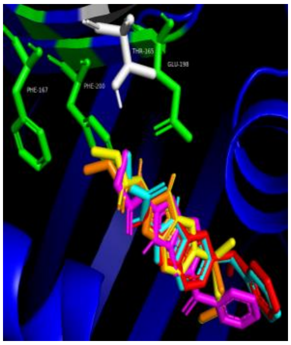

ALF- all drugs

Supplementary Figure S39: Autodock vina docking results for *A. lumbricoides* isotype F and several benzimidazole drugs. The drugs used are albendazole (ABZ), albendazole sulfoxide (ABZSO), fenbendazole (FBZ), mebendazole (MBZ) and oxfendazole (OXBZ). 3D and 2D models are shown for each docking result. Binding affinities are shown underneath each model. In ABZ both models show two H-bonds with both Q134 and E198 but in the 2D model an extra H-bond with T165 is also shown. For ABZSO two H-bonds are shown with Q134 and E198 and one H-bond is formed with T165 and L253 in the 3D model, however in the 2D model the bond with L253 is not found. In FBZ two H-bonds are formed with E198 and one bond is formed with Q143 and T165 in both models. The 3D model for MBZ shows single H-bonds with Q134 and E198 however the 2D model shows only the bond with E198. Finally, both models for OXBZ predict H-bond formation with T165 and E198.

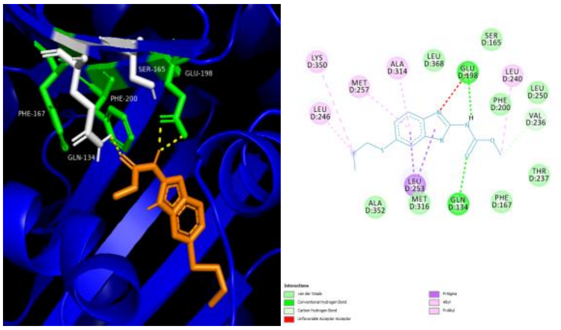

ASG- ABZ-4.1 kcal/mol

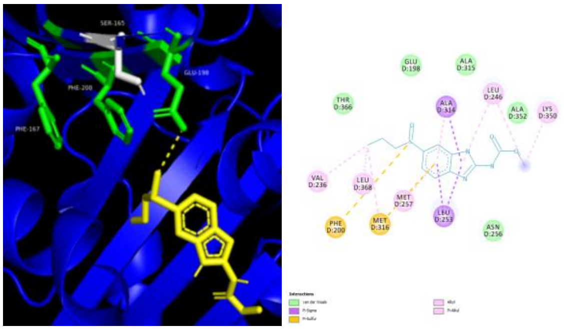

ASG- ABZSO -4.1 kcal/mol

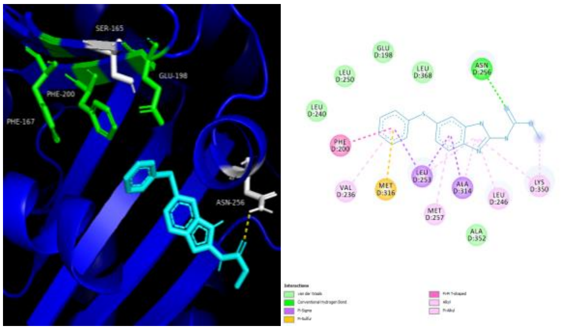

ASG- FBZ -6.2 kcal/mol

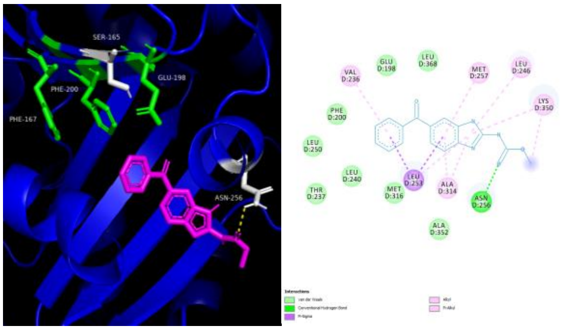

ASG- MBZ -6.9 kcal/mol

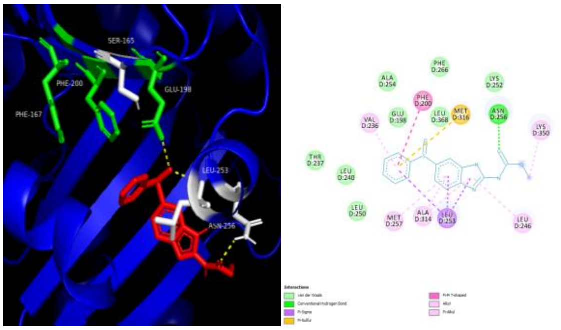

ASG- OXBZ -5.2 kcal/mol

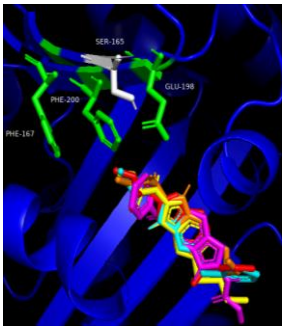

ASG- all drugs

Supplementary Figure S40: Autodock vina docking results for A. suum isotype G and several benzimidazole drugs. The drugs used are albendazole (ABZ), albendazole sulfoxide (ABZSO), fenbendazole (FBZ), mebendazole (MBZ) and oxfendazole (OXBZ). 3D and 2D models are shown for each docking result. Binding affinities are shown underneath each model. The interactions between ASG and ABZ form 2 H-bonds with E198 and 1 with Q134 in both 2D and 3D models, however one of the H-bonds with E198 is predicted to be an unfavourable acceptor to acceptor bond in the 2D model. For ABZSO a single H-bond is formed with E198 in the 3D model but no bonds are seen in the 2D model. In the cases of FBZ and MBZ a single H-bond to N265 is seen in both 3D and 2D models. Finally for OXBZ 1 H-bond is formed with E198, L253 and N256 in the 3D model but only a single H-bond is predicted with N256 in the 2D model.

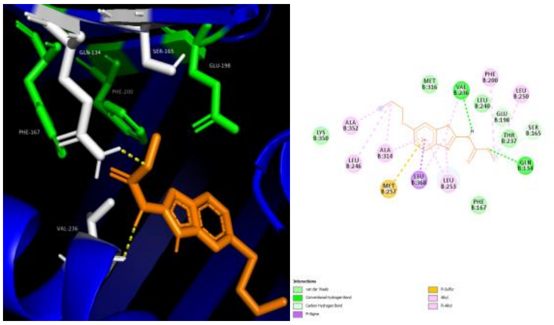

ALG- ABZ -6.2 kcal/mol

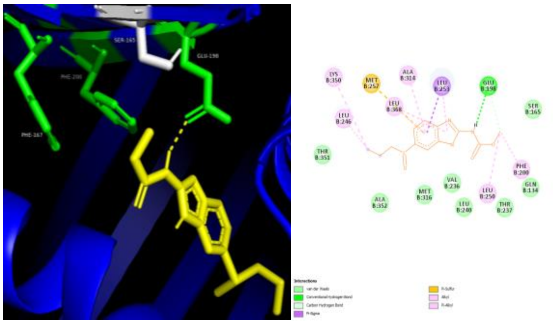

ALG- ABZSO -6.1 kcal/mol

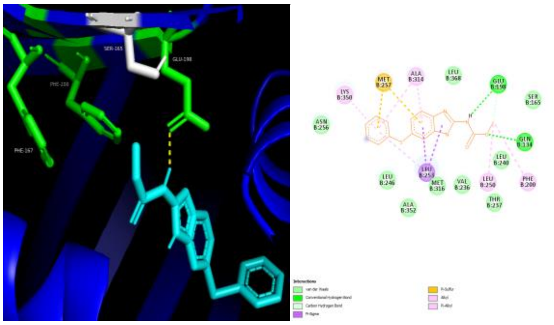

ALG- FBZ -7 kcal/mol

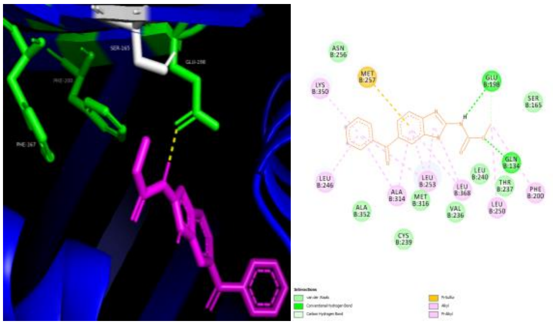

ALG- MBZ -7.8 kcal/mol

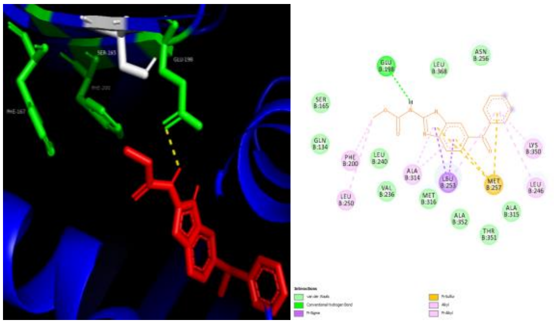

ALG- OXBZ -5.9 kcal/mol

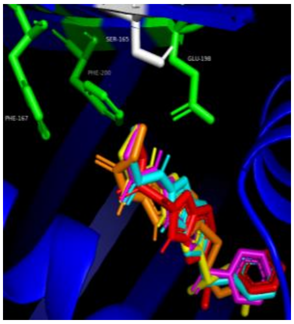

ALG- all drugs

Supplementary Figure S41: Autodock vina docking results for *A. lumbricoides* isotype L and several benzimidazole drugs. The drugs used are albendazole (ABZ), albendazole sulfoxide (ABZSO), fenbendazole (FBZ), mebendazole (MBZ) and oxfendazole (OXBZ). 3D and 2D models are shown for each docking result. Binding affinities are shown underneath each model. In ABZ both models find H-bonds with Q134 and V236. Both models for ABZSO and OXBZ show H-bonding with only E198. For FBZ and MBZ the 3D models show H-bonds with E198, whereas the 2D models find H-bond formation with Q134 and E198.
